# Construction of mannosylated glycol-nanoparticles for near-infrared bioimaging and photodynamic therapy targeting breast cancer cells

**DOI:** 10.1039/d6ra05981c

**Published:** 2026-08-21

**Authors:** Yu Tang, Zhu-Ting Song, Lu-Lu Sun, Meng Sun, Ming-Yu Wang, Hai-Hao Han, Jin Gong, Lei Dong, Tan Ke

**Affiliations:** a School of Pharmacy, Anhui University of Chinese Medicine Hefei 230031 China; b School of Life Science and Technology, Shandong Second Medical University Weifang 261053 P.R. China; c Shandong Laboratory of Yantai Drug Discovery, Bohai Rim Advanced Research Institute for Drug Discovery Yantai 264117 China; d Molecular Imaging Center, Shanghai Institute of Materia Medica, Chinese Academy of Sciences Shanghai 201203 China; e School of Pharmacy, Shandong Second Medical University Weifang 261053 P. R. China; f Educational Technology Center, The PLA General Hospital Beijing 100853 China

## Abstract

Photodynamic therapy (PDT) offers a promising approach for cancer treatment, but developing photosensitizers (PSs) with tumor targeting and near-infrared (NIR) imaging capabilities remains challenging. Herein, we designed two donor–π–acceptor (D–π–A) type photosensitizers, TC1 and TC2, by bridging electron-rich carbazole with electron-withdrawing tricyanofuran (TCF) *via* thiophene or EDOT moieties. Both PSs exhibited broad absorption, NIR fluorescence emission (>650 nm), and efficient type-I/II reactive oxygen species (ROS) generation under light irradiation. To improve aqueous dispersibility and breast cancer targeting, we conjugated PEGylated mannose to TC1 and TC2, yielding glycol-nanoparticles TC1M and TC2M. These nanoparticles maintained desirable NIR optical properties and ROS generation capacity while exhibiting excellent biocompatibility. Notably, mannose receptor-mediated endocytosis enabled selective uptake by MDA-MB-231 breast cancer cells over normal bEnd.3 cells, allowing targeted fluorescence imaging. Furthermore, TC2M demonstrated potent photodynamic activity, reducing cell viability to 23.15% at 20 µM under light irradiation through efficient intracellular ROS generation. This study presents mannose-functionalized glycol-nanoparticles as promising targeted theranostic agents for NIR imaging and PDT of breast cancer.

## Abstract 2

Introduction of mannose in photosensitizer facilitates the water dispersity and biocompatibility, endowing the cell targeting through “carbohydrate-receptor” recognition, and thereby improving bioimaging and photodynamic therapy against cancer cells.

## Graphical abstract

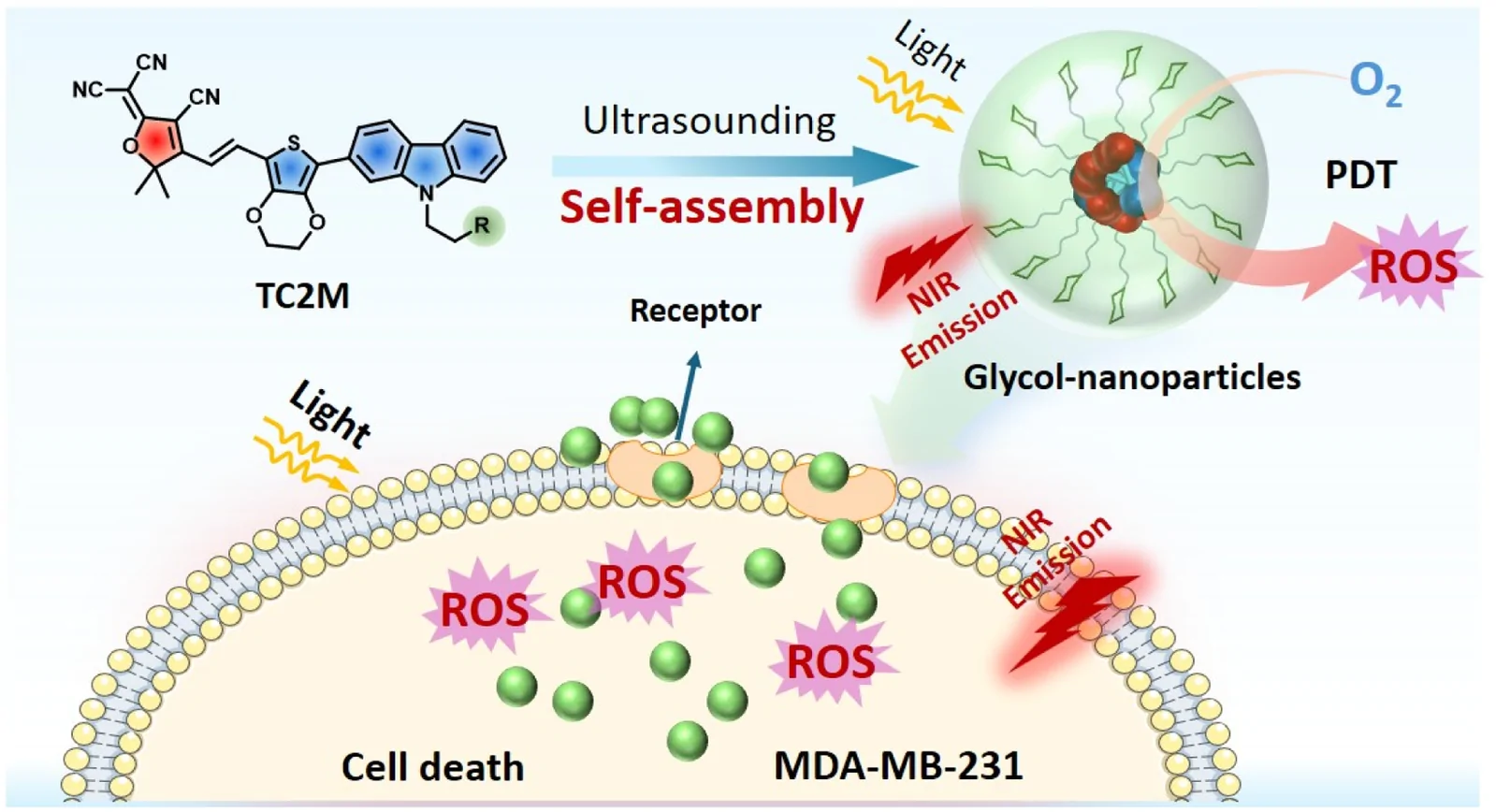

## Introduction

Breast cancer remains one of the most prevalent malignancies worldwide, with triple-negative breast cancer (TNBC) representing a particularly aggressive subtype characterized by poor prognosis due to the lack of effective therapeutic targets.^1^ Precise diagnosis and targeted therapy are critical for improving patient outcomes, yet current clinical imaging modalities, such as magnetic resonance imaging (MRI) and computed tomography (CT), lack real-time intraoperative guidance and cellular-level resolution.^2^ Fluorescence imaging offers high sensitivity, but conventional visible-light probes (400–650 nm) suffer from poor penetration and strong autofluorescence, limiting their application in deep-seated tumor visualization. Moreover, existing targeted imaging agents often fail to integrate therapeutic functions, necessitating separate diagnostic and treatment procedures.^3^ To address these challenges, near-infrared (NIR, >650 nm) fluorescence imaging combined with photodynamic therapy (PDT) has emerged as a promising theranostic strategy, enabling deep visualization and simultaneous non-invasive tumor ablation. However, the development of NIR probes with both breast cancer targeting capability and efficient PDT remains an unmet need.^4,5^

The efficacy of PDT critically depends on photosensitizers (PSs), which convert molecular oxygen into cytotoxic reactive oxygen species (ROS) upon light irradiation, triggering tumor cell death through pathways such as apoptosis, ferroptosis, and pyroptosis.^6–8^ ROS generation by PSs primarily follows two distinct mechanisms: (i) in type-II PDT, energy transfer from excited PSs to molecular oxygen yields singlet oxygen (^1^O_2_). (ii) Alternatively, type-I PDT operates *via* electron transfer, producing radicals such as superoxide ion (O_2_˙^−^) and hydroxyl radicals (˙OH), or directly oxidizing biomolecules like NADH and DNA.^9–11^ Recent advances in PS development extend beyond traditional small molecules (such as BODIPYs, porphyrins, and phthalocyanines) to include supramolecular nanoparticle assemblies. These structures have proven effective in boosting type-I ROS production and integrating additional biological functionalities.^12–14^ Although numerous PSs have been developed, their biological application remains hindered by several challenges. A prominent issue is poor water solubility, which compromises cellular uptake and biocompatibility. Therefore, developing water-soluble nano-PSs is paramount to enhance biocompatibility and cellular internalization for effective PDT.

Introduction of hydrophilic and biological groups into PSs' structures, including water-soluble sulfonic moiety, peptide and polyethylene glycol (PEG) linker, can significantly facilitate the biocompatibility and cellular endocytosis.^15–17^ However, most of the above modification strategies fail to confer targeting capability toward breast cancer cells. This issue can be effectively addressed by modifying the molecular structure with mannose. Mannose not only enhances the water solubility of PS but also enables specific recognition of receptors overexpressed on the surface of breast cancer cells, thereby promoting targeted cellular uptake and precise treatment of tumor cells. At present, some mannose targeted PSs were reported for cancer phototherapy (Table S1).^18,19^ Developing near-infrared (>650 nm) emitting mannosylated nano-PSs holds importance for targeted cell bioimaging and PDT applications.^20^

**Fig. 1:**
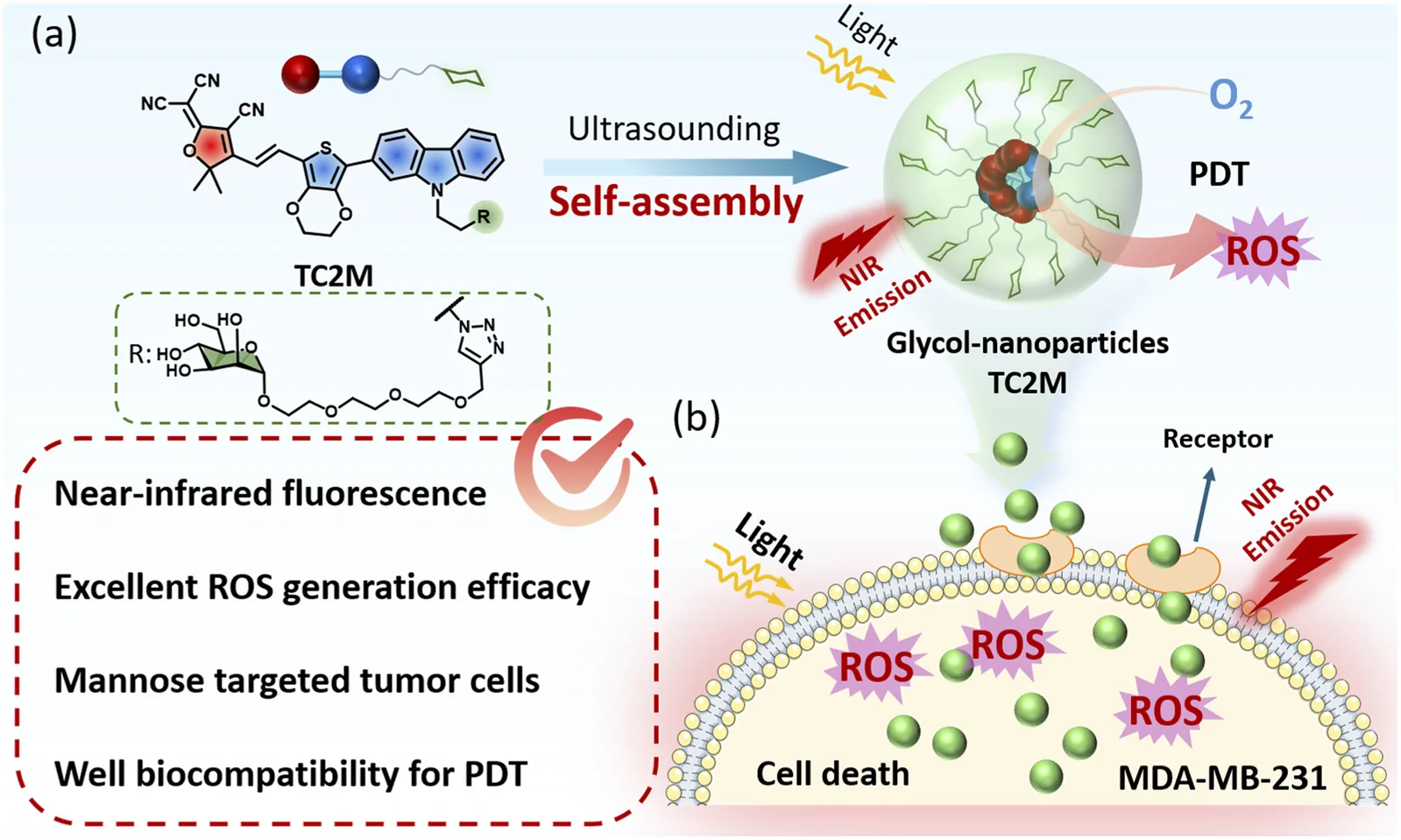
(a) Mannose-modified TCF-based photosensitizer (TC2) self-assembled to form glycol-nanoparticles (TC2M). (b) MDA-MB-231 cell-targeted NIR imaging and PDT through mannose-receptor recognition.

Here, we presented two mannose-decorated glycol-nanoparticles for breast cancer cell-targeted NIR bioimaging and efficient photodynamic therapy (Fig. 1). Electron-withdrawing tricyanofuran (TCF) and electron-rich carbazole were bridged by thiophene or its derivatives to obtain two PSs TC1 and TC2. Two PSs exhibited the broad absorption bands and NIR fluorescence, influencing by intramolecular charge transfer (ICT) effect. In aqueous environment, the aggregated PSs could high-efficiently sensitize surrounding oxygen to ROS under 590 nm light irradiation. Two PSs were then introduced the PEG-modified mannose and formed the glycol-nanoparticles (TC1M and TC2M), facilitating the water-dispersibility and biocompatibility and still remaining ROS generation capability. Mannose receptor-mediated endocytosis of glycol-nanoparticle TC2M enabled selective uptake by MDA-MB-231 breast cancer cells, allowing targeted fluorescence imaging. Notably, TC2M exhibited potent photodynamic activity, reducing cell viability to 23.15% at 20 µM under light irradiation through efficient ROS generation. This work emphasized on the mannose-modified glycol-nanoparticles construction for cancer cell-targeted NIR theranostics strategy.

## Results and discussion

### PS design and photophysical properties

**Fig. 2:**
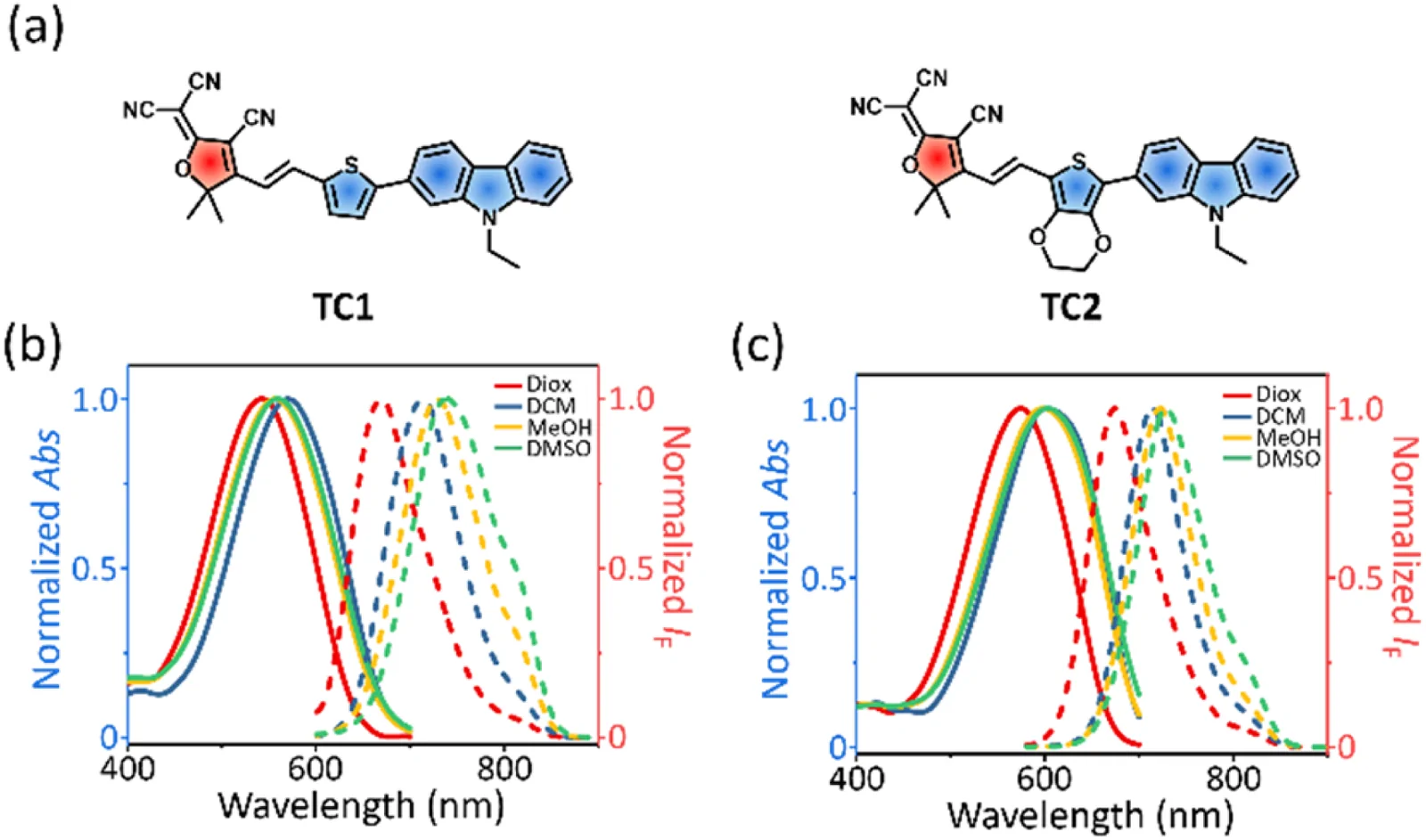
(a) Chemical structures of PS TC1 and TC2. Normalized absorption and fluorescence spectra of (b) TC1 and (c) TC2 in four organic solvents, including 1,4-dioxane (Diox), dichloromethane (DCM), methanol (MeOH) and dimethylsulfoxide (DMSO).

We designed and synthesized two tricyanofuran (TCF) based photosensitizers (TC1 and TC2) with electronic “donor–π–acceptor (D–π–A)” structures. The “D–π–A” structure can improve charge separation state upon photoexcitation, thus facilitating the ROS generation exposed under light irradiation. We selected TCF and *N*-ethylcarbazole as electron-withdraw and electron-rich sides, respectively. Thiophene (TC1) and 3,4-ethoxylene dioxy thiophene (TC2) bridged the electronic donor and acceptor to further expand the intramolecular π-conjugation and red-shifted the fluorescence to near-infrared (NIR) window (Fig. 2a). The synthesis of two PSs experienced two steps: (i) thiophene or its derivatives was combined with 3-(*N*-ethyl-carbazolyl)boronic acid catalyzed by Suzuki–Miyaura cross-coupling reaction, (ii) and then conjugated with TCF moiety through Knoevenagel condensation (Scheme S1). The corresponding characteristics including ^1^H NMR, ^13^C NMR and high-resolution mass spectrometry (HRMS) have been provided in SI.

With two PSs in hand, we initially explored the photophysical properties of TC1 and TC2 in different organic solvents, including dichloromethane (DCM), 1,2-dichloroethane (DCE), tetrahydrofuran (THF), 1,4-dioxane (Diox), acetonitrile (CH_3_CN), methanol (MeOH), dimethylsulfoxide (DMSO) and dimethyl formamide (DMF). Both of two PSs showed broad absorption bands and near-infrared fluorescence spectra (>650 nm) in series of organic solvents (Fig. 2b and c). The absorption band of TC1 covers 450–650 nm with absorbance maximum at ∼575 nm (*ε* = 3.5 × 10^4^ M^−1^ cm^−1^) in DCM, whereas the absorption region of TC2 locates on 500–700 nm with maximum at ∼610 nm (*ε* = 6.4 × 10^4^ M^−1^ cm^−1^) in DCM (Fig. S1). TC1 and TC2 exhibited similar fluorescence spectrum with maximal intensity at *ca.* 725 nm with quantum yield (*Φ*_F_) as 0.16 and 0.14 in DCM, respectively (Fig. S2). The large stokes shift and polar influenced fluorescence in solvents might be attributed the intramolecular charge transfer (ICT) effect (Fig. 2b and c).^21^

**Fig. 3:**
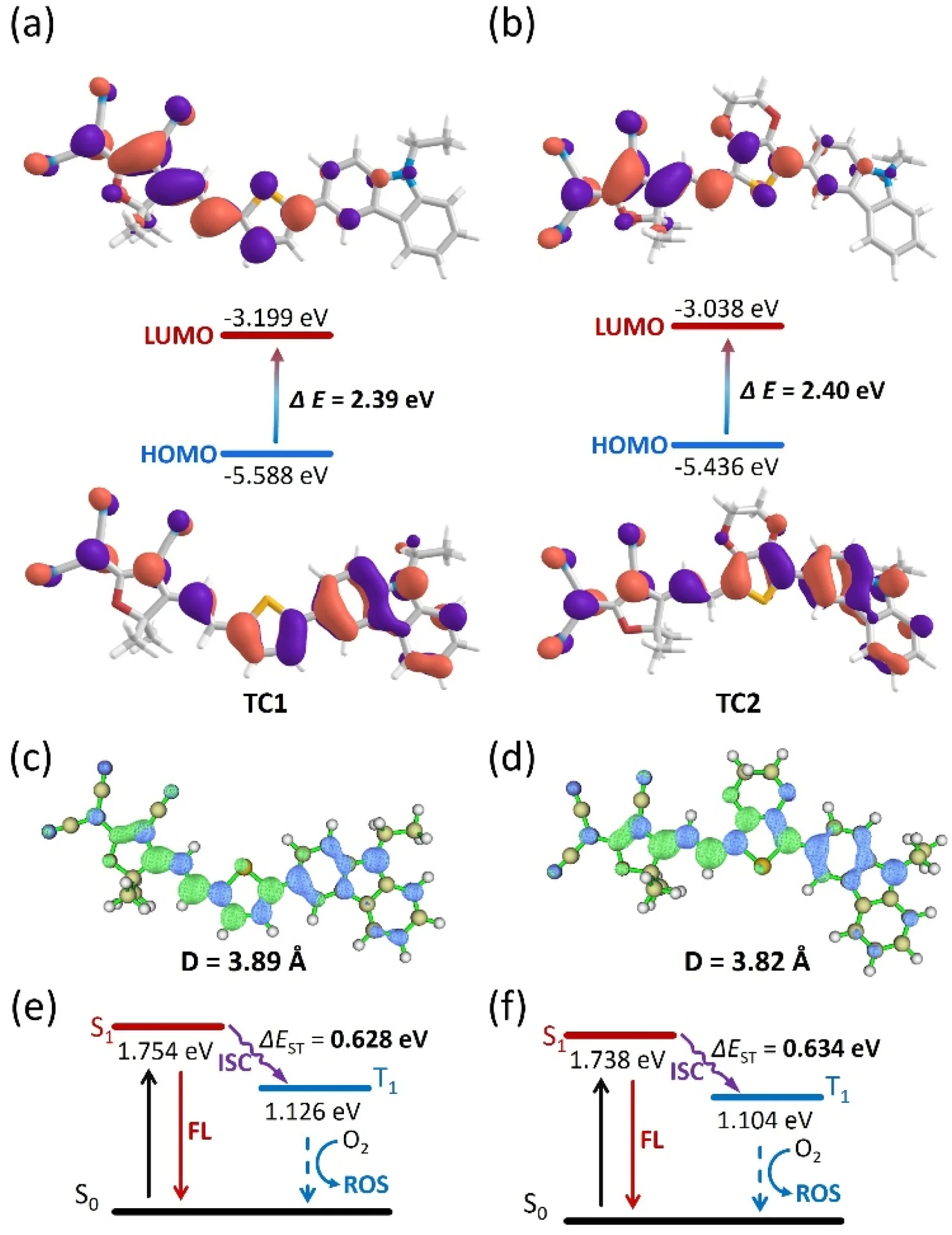
DFT calculated the HOMO–LUMO distribution of (a) TC1 and (b) TC2, the hole–electron distribution of (c) TC1 and (d) TC2 in excited state, the energy gaps in S_1_ state and T_1_ state of (e) TC1 and (f) TC2. DFT calculation was used the theory of B3LYP/6-31G(d,p) level in the gas phase.

To verify our hypothesis, we calculated the optimal spatial conformation, the highest occupied molecular orbitals (HOMOs), the lowest unoccupied molecular orbitals (LUMOs), the excited energy of the S_1_ and T_1_ state, hole–electron distributions of two PSs in excited state by using functional density theory (DFT).^22–24^ Two PSs possess the planar molecular conformations with small dihedral angles between TCF, thiophene and carbazole moieties (Fig. S3), that allowing the intramolecular electron transfer from donor (carbazole) to acceptor (TCF). TC1 and TC2 exhibited significant electron separation, that the HOMOs were mainly localized on the whole molecules, meanwhile the LUMOs were nearly delocalized across the TCF and thiophene bridge backbones (Fig. 3a and b). The similar energy gaps (Δ*E*) of two PSs between HOMOs and LUMOs were in agreement with the measured absorption spectra. The hole–electron distributions of two PSs at excited state revealed that the holes were concentrated on the electron-rich carbazole part, whereas electrons were exclusively located on the TCF and thiophene constituted π-backbone, verifying the ICT effect predominantly influenced the photophysical properties (Fig. 3c and d). Moreover, the excitation energies of S_1_ state were calculated to be 1.754 eV for TC1 and 1.738 eV for TC2, indicating the slightly longer emission wavelength of TC2 than TC1 in organic solvents (Fig. 3e and f).

**Fig. 4:**
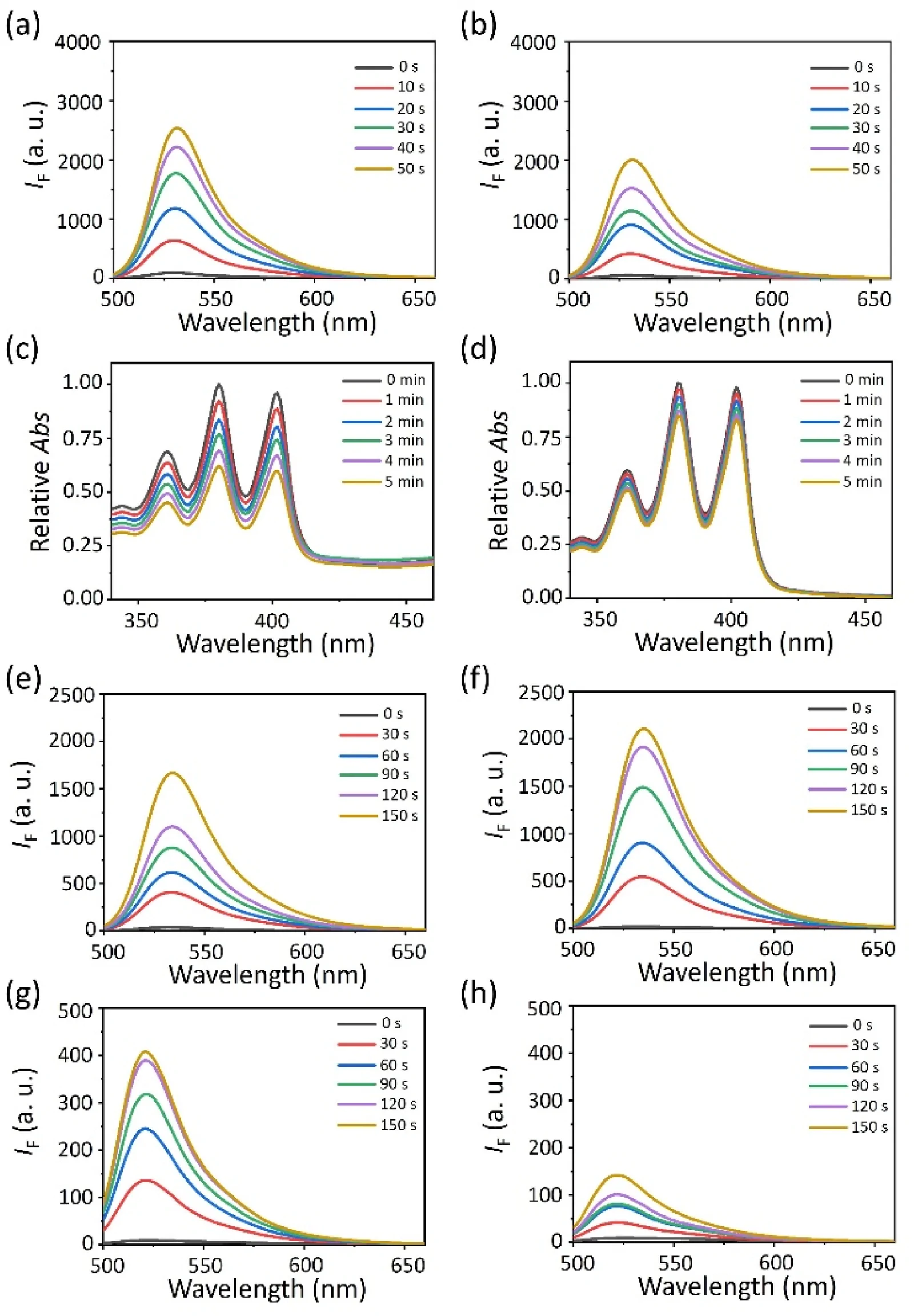
The fluorescence spectra of DCFH containing (a) TC1 and (b) TC2 under light irradiation within 50 s. The absorption spectra of ABDA containing (c) TC1 and (d) TC2 under light irradiation within 5 min. The fluorescence spectra of DHR123 containing (e) TC1 and (f) TC2 under light irradiation within 150 s. The fluorescence spectra of HPF containing (g) TC1 and (h) TC2 under light irradiation within 150 s. (*c* = 10 µM, 1× PBS, pH 7.4, 590 nm light, *P* = 40 mW cm^−2^).

DFT determined the energy level of T_1_ state and calculated the energy gap between S_1_ and T_1_ state as 0.628 eV and 0.634 eV for TC1 and TC2, respectively, allowing to sensitize surrounding oxygen to ROS through PDT process (Fig. 3e and f). Therefore, we assessed the ROS generation efficacy of two PSs in PBS buffer. We explored 2′,7′-dichloroflurescein (DCFH), a commercial indicator, to monitor the total ROS generation of TC1 and TC2 in PBS (Fig. S4a).^25^ Under 590 nm LED light irradiation (*P* = 40 mW cm^−2^), both two PSs could trigger the fluorescence enhancement within 50 s, in contrast to the feeble emission intensity from the negative control only containing DCFH (Fig. 4a, b and S5a). These results probably indicated that two PSs might possess PDT capability in PBS buffer. To further investigated the generated ROS species, 9,10-anthracenediyl-bis(methylene)dimalonic acid (ABDA) was enlisted to monitor the singlet oxygen generation (Fig. S4b). Upon increasing irradiation time, the classic triplet absorption peaks of ABDA were gradually disappeared due to structural change of ABDA after capturing ^1^O_2_ generated by TC1 and TC2 (Fig. 4c, d and S5b). Meanwhile, dihydrorhodamine 123 (DHR123) and hydroxyphenyl fluorescein (HPF) was utilized to determine the O_2_˙^−^ and ˙OH radicals (Fig. S4c and d). We observed the characteristic fluorescence emission at 535 nm and 525 nm for DHR123 and HPF, respectively, when two PSs were exposed under light irradiation for 150 s (Fig. 4e–h). However, the blank samples without PSs existence could not cause the spectral variations of ROS indicators (Fig. S5c and d). These data manifested that TC1 and TC2 could generate multiple ROS species through type-I and type-II pathways. In addition, no distinct change of absorbance for two PSs was observed whether existing in PBS buffer for 7 days, or exposed under light irradiation for 50 min, exhibiting outstanding stability and photostability in PBS buffer (Fig. S6).

### Mannosylation and glycol-nanoparticles formation

Even though two PSs possess the satisfied NIR emission and ROS generation capability, the poor compatibility and lacking cell recognition still restricted their further bioapplications in bioimaging and PDT against breast cancer. Utilization of the recognition between mannose and specific receptor protein on surface of MDA-MB-231 cells (triplet negative breast cancer), we introduced mannosides into two PSs to obtain the glycol-PSs TC1M and TC2M. In consideration to the modifiability on *N*-ethyl site of carbazole, we decorated the azido groups into ethyl part, that could further combine with alkynyl-polyethyleneglycol (PEG) modified mannose through Cu(i)-catalyzed cycloaddition. After hydrolysis in basic aqueous environment, we obtained the glycol-PSs TC1M and TC2M with high water-dispersity (Scheme S2). The synthetic procedures and corresponding characteristics were shown in SI.

**Fig. 5:**
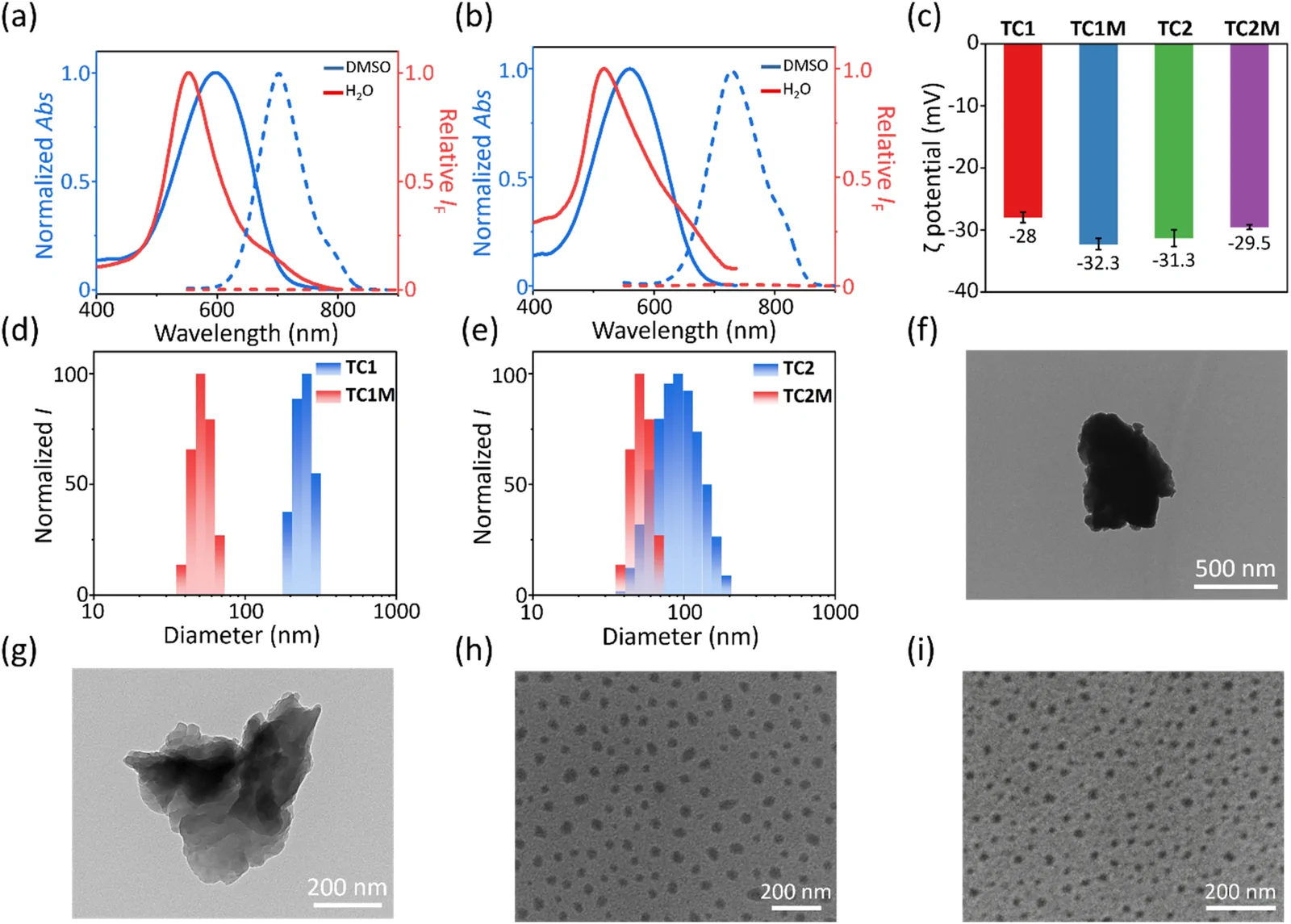
Normalized absorption (full line) and fluorescence spectra (dash line) of (a) TC1M and (b) TC2M dissolved in DMSO (blue) or dispersed in PBS buffer (red). (c) *ζ* Potential of four PSs dispersed in PBS buffer. DLS size distributions of (d) TC1 and TC1M and (e) TC2 and TC2M (blue: TC1/TC2; red: TC1M/TC2M). TEM morphologies of (f) TC1, (g) TC2, (h) TC1M and (i) TC2M.

The photophysical properties of two glycol-PSs were firstly investigated. Due to the hydrophilic mannoside, TC1M and TC2M were difficult to dispersed in low-polar solvents, but could well dissolved in DMSO or DMF. Two glycol-PSs still maintained the singlet and broad absorption bands with absorbance maxima at 585 nm and 590 nm, and NIR fluorescence spectra with maxima at 715 nm and 730 nm for TC1M and TC2M in DMSO, respectively (Fig. 5a and b). The similar absorption and emission spectra of two glycol-PSs were similar as the molecules without mannoside decoration, revealing that mannosylation might not influence the optical properties. The optimal conformation and HOMO–LUMO distributions of TC1M and TC2M were also calculated by using DFT. We observed that the introduced mannosides was independent of the PS backbone and only connected by flexible PEG chains (Fig. S7). The HOMO–LUMO distributions of two glycol-PSs kept distinct electron separation, indicating the ICT effect still dominate the photophysical properties of TC1M and TC2M (Fig. S8).

Hydrophilic mannoside and PEG chains could not provide water-dispersity to meet PS well-formed nanoparticles in PBS buffer. We speculated TC1M and TC2M might form the glycol-nanoparticles under ultrasounding. The aggregated PS backbone constituted the hydrophobic core, while hydrophilic mannoside and PEG chains might be unfolded to provide sufficient water-solubility. Two resulting glycol-nanoparticles exhibited slightly blue-shifted absorption bands with quenched fluorescence, compared with the glycol-PSs in monomer (Fig. 5a and b). Two glycol-nanoparticles possess negative *ζ* potentials as −32.3 mV and −29.5 mV for TC1M and TC2M, respectively, that have no significant difference from TC1 and TC2 in PBS buffer (Fig. 5c). To further investigate the glycol-nanoparticles, we determined the hydrodynamic diameters of the four PSs using dynamic light scattering (DLS). After sonication, TC1M and TC2M showed the smaller hydrodynamic diameters and narrower dispersion (red) than TC1 and TC2 formed aggregates (Fig. 5d and e). Then, transmission electron microscopy (TEM) was utilized to explore the morphologies of two PSs and their corresponding glycol-nanoparticles. The aggregates formed by TC1 and TC2 exhibited morphologies of multiple irregular sheet-like solids stacked into bulk structures with large volumes (Fig. 5f and g). In contrast, TC1M and TC2M decorated with mannose and PEG chains provided enough water-dispersity, exhibiting smaller volumes and relatively uniform irregular spherical morphologies (Fig. 5h and i). These distinct morphological differences demonstrated that the incorporation of mannose and PEG chains alters the aggregation pattern of PSs. The smaller particle size could also facilitate cellular uptake, thereby enhancing biocompatibility. Next, four kinds of ROS indicators, DCFH, ABDA, DHR123 and HPF, were monitored the ROS generation efficacies of two glycol-nanoparticles in PBS. The remarkable signal changes of indicators manifested that TC1M and TC2M could still sensitize oxygen to ROS under 590 nm light irradiation (*P* = 40 mW cm^−2^, Fig. S9–S11). The ROS generation performance of aggregated TC2M was further verified by electron spin resonance (ESR) spectroscopy, that captured the characteristic signals of ^1^O_2_, O_2_˙^−^ and ˙OH radicals (Fig. S12). However, introduction of mannoside and PEG chains appreciably impaired the ROS generation efficiency of two glycol-nanoparticles in comparison to TC1 and TC2 alone in PBS (Fig. S11). We speculated that modification of hydrophilic moieties might decrease the aggregation of PSs, thus hindering the ROS generation through pathway of aggregation-induced generation of ROS.^25,26^ The water-stability in room temperature and photostability of TC1M and TC2M were measured, that allowed for next evaluation in cells (Fig. S13).

### NIR bioimaging and PDT in cancer cells

Prior to cell imaging and photodynamic therapy (PDT) efficacy evaluations, we first assessed the cytotoxicity of TC1M and TC2M against MDA-MB-231 human breast cancer cells. Cell viability was determined using the CCK-8 assay after 24 h of co-incubation with varying concentrations (0–20 µM, Fig. S14). The results demonstrated that cell survival rates remained above 95% following treatment with both TC1M and TC2M, indicating their excellent cytocompatibility.

**Fig. 6:**
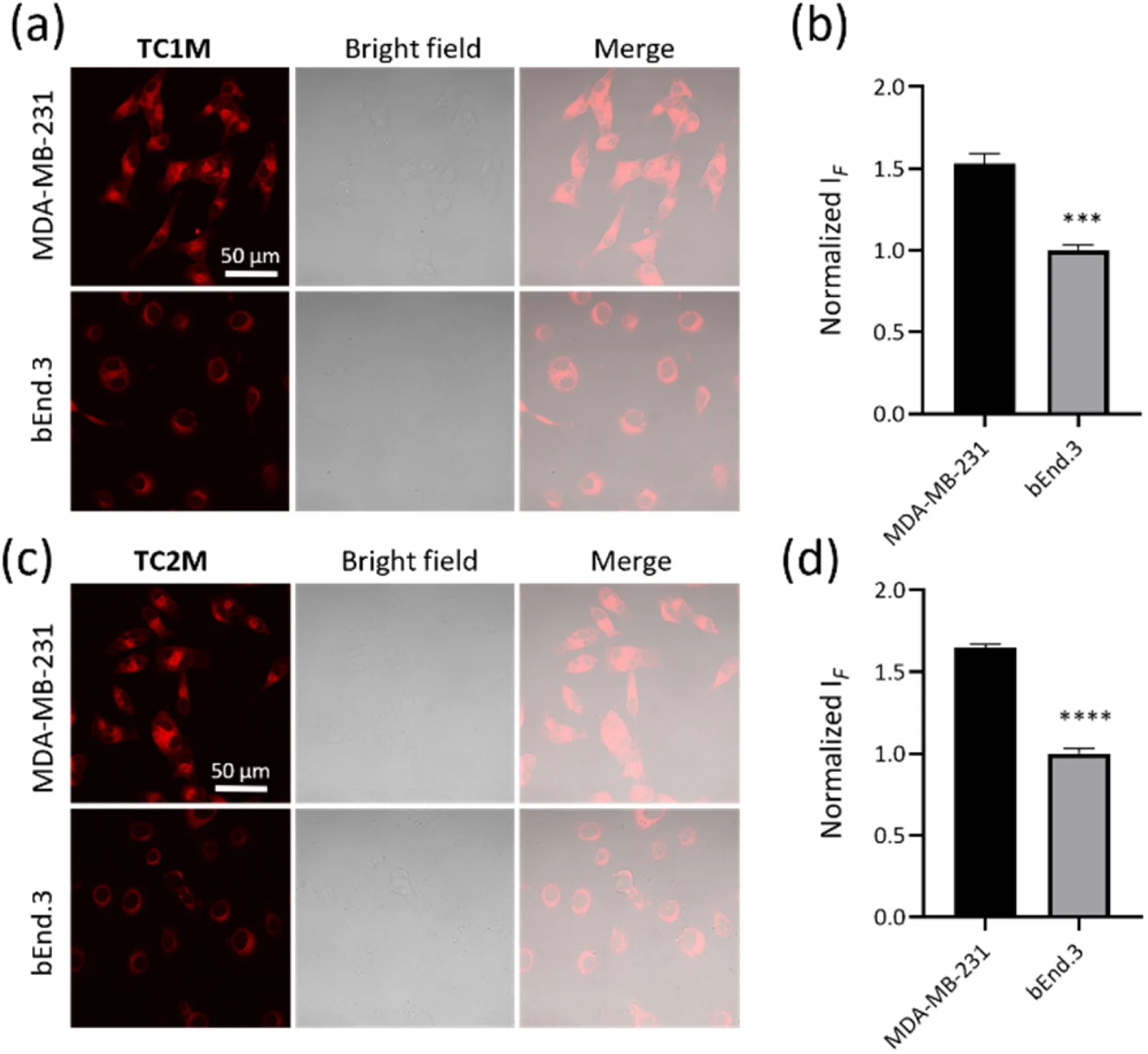
Laser confocal microscope imaging and quantification of MDA-MB-231 and bEnd.3 cells incubating with (a and b) TC1M (1 µM) or (c and d) TC2M (1 µM). *λ*_ex_/*λ*_em_ = 561/650–750 nm for TC1M and TC2M. Data are represented as mean ± SD of three parallel experiments and analyzed by *t*-test. Statistical analysis: ***p < 0.001, ****p < 0.0001. Scale bar = 50 µm.

We next investigated whether the probes TC1M and TC2M could distinguish between tumor cells and normal cells. To this end, MDA-MB-231 human breast cancer cells and bEnd.3 mouse brain endothelial cells were incubated with TC1M or TC2M for 4 h, followed by confocal laser scanning fluorescence imaging. The results revealed that both probes exhibited strong fluorescence signals in MDA-MB-231 cells, whereas only weak fluorescence was observed in bEnd.3 cells (Fig. 6). This difference is likely attributable to the high expression of mannose receptors on the surface of MDA-MB-231 breast cancer cells, which specifically recognize and actively internalize mannose-modified fluorescent molecules.^27^

**Fig. 7:**
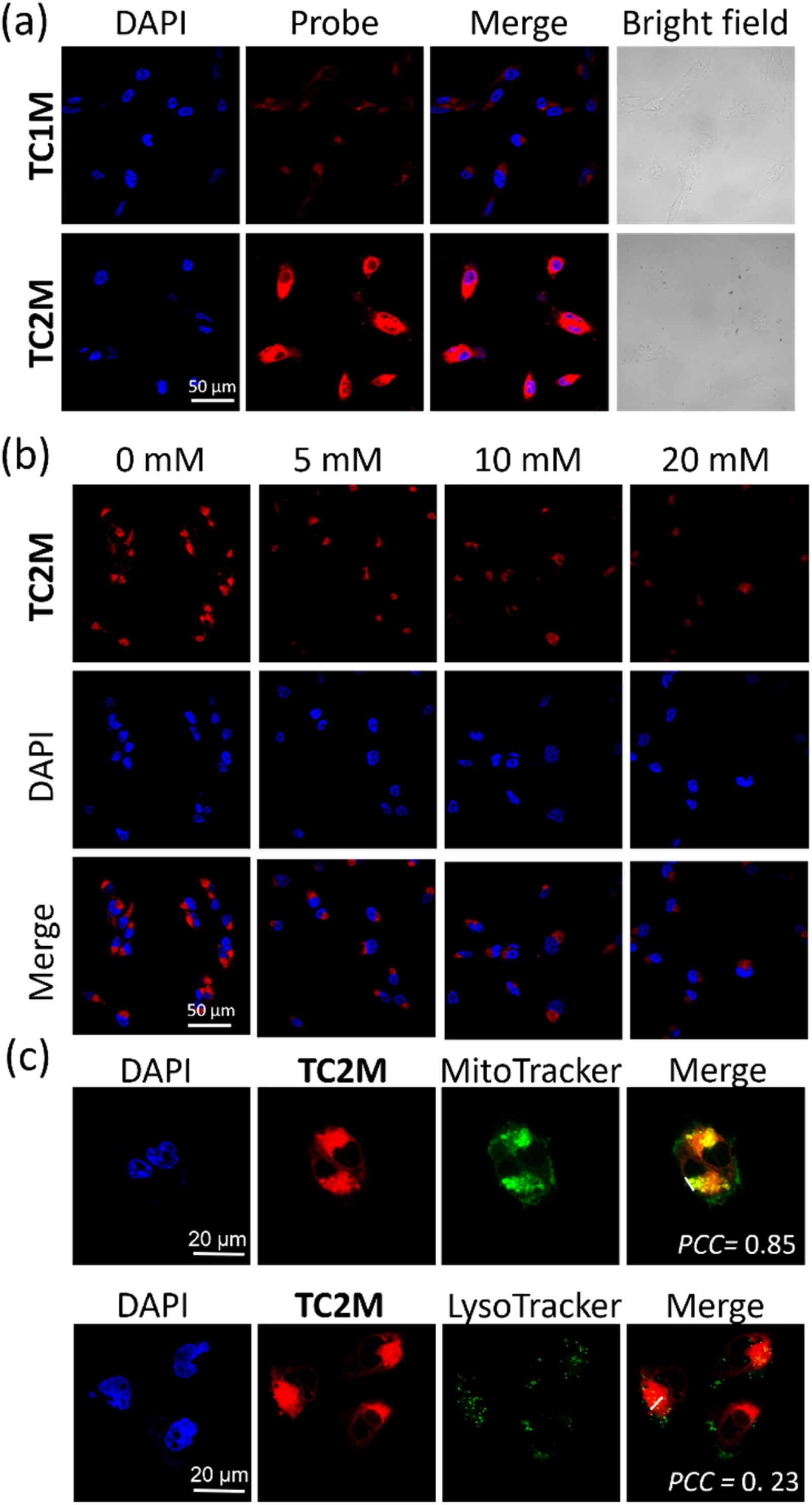
(a) Fluorescence imaging of MDA-MB-231 cells incubating with TC1M (1 µM) or TC2M (1 µM) for 4 h. (b) Fluorescence imaging of MDA-MB-231 cells co-incubating with TC2M (1 µM) and different concentrations (0, 5, 10 and 20 mM) of mannose for 4 h. (c) Fluorescence colocalization of TC2M with MitoTracker (a mitochondria tracker, 100 nM) and LysoTracker (a lysosome tracker, 100 nM) in MDA-MB-231 cells. *λ*_ex_/*λ*_em_ = 561/650–750 nm for TC2M. *λ*_ex_/*λ*_em_ = 488/500–560 nm for trackers.

To further evaluate the cellular uptake of two PSs, we collected the fluorescence images of MDA-MB-231 cells after incubation with TC1M and TC2M (1 µM) for 4 h. Cells incubated with TC2M exhibited approximately 1.8-fold higher fluorescence intensity than those incubated with TC1M (Fig. 7a and S15). To confirm that cellular uptake of the PS is mediated by mannose-receptor recognition, we performed a competition assay by co-incubating cells with the same concentration of TC2M (1 µM) and increasing concentrations (0, 5, 10 and 20 mM) of free mannose. We observed a marked decrease in intracellular fluorescence signals upon increasing mannose concentration, suggesting that the mannose moietieson the PSs compete with free mannose for binding the receptor on cells (Fig. 7b and S16). These findings demonstrate that both PSs enable effective targeted fluorescence imaging of breast cancer cells and hold potential for distinguishing tumor cells from normal cells.

To investigate the subcellular localization, TC1M and TC2M were co-stained with two commercial trackers targeting mitochondria and lysosomes, respectively, in MDA-MB-231 cells. Upon comparing the fluorescence signals of two PSs in the green and red channels with those of the commercial organelle trackers, we observed that the high colocalization between red fluorescence of TC2M and the green signal from the mitochondrial tracker, as reflected by Pearson's correlation coefficients (PCC) of 0.85 (Fig. 7c and S17a), whereas TC1M exhibited poor colocalization on mitochondria with low PCC of 0.45 (Fig. S18a and c). In addition, we found that neither two PSs were appreciably enriched in lysosomes, confirming by the low PCC of 0.30 for TC1M and 0.23 for TC2M (Fig. 7c, S17, S18b and d).

**Fig. 8:**
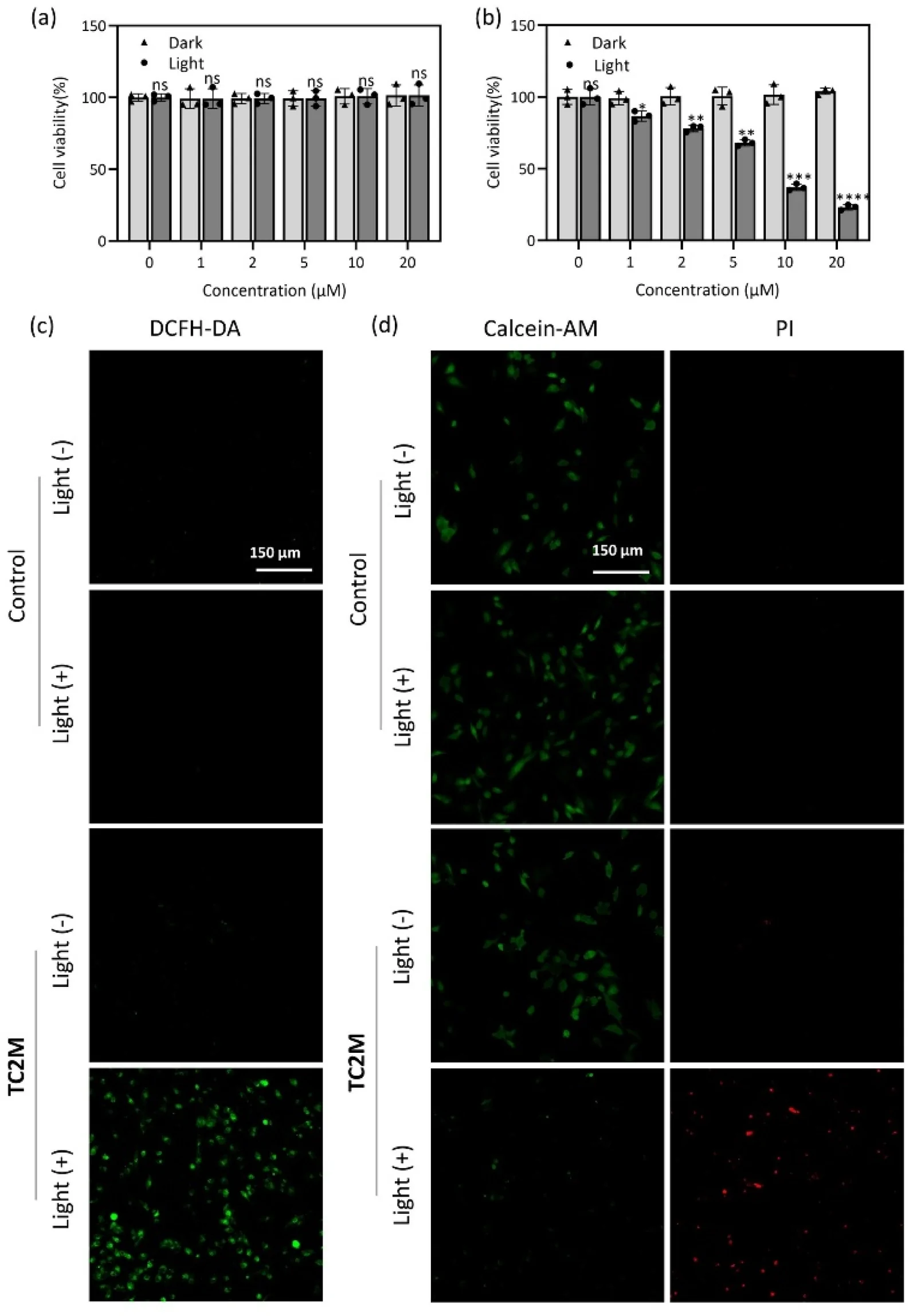
The cell viability of MDA-MB-231 cells stained with different concentration (0–20 µM) of (a) TC1M and (b) TC2M, and then irradiated with or without white LED light (80 mW cm^−2^, 40 min). (c) DCFH-DA sensing the intracellular ROS generation after treating with TC2M (2 µM) and irradiating with or without white LED light (80 mW cm^−2^, 40 min). (d) Calcein-AM (green) and propidium iodide (PI, red) co-staining for live–dead MDA-MB-231 cells imaging after treating with TC2M (2 µM) and irradiating with or without white LED light (80 mW cm^−2^, 40 min). Scale bar = 150 µm. Error bars: mean ± SD (*n* = 3). *p < 0.05,**p < 0.01, ***p < 0.001, ****p < 0.0001, ns = no significant.

We next evaluated whether TC1M and TC2M could achieve targeted tumor cell killing *via* PDT. MDA-MB-231 cells were incubated with various concentrations (0–20 µM) of TC1M or TC2M for 4 h, and then kept in the dark or exposed to white LED light irradiation (80 mW cm^−2^) for 40 min to assess dark cytotoxicity and phototoxicity, respectively. As shown in Fig. 8a and b, under light irradiation, TC2M-treated MDA-MB-231 cells exhibited significant concentration-dependent cytotoxicity, with cell viability decreasing to 23.15% at a concentration of 20 µM. In contrast, TC1M showed no obvious phototoxicity under the same conditions. Under dark conditions, neither TC1M nor TC2M exhibited significant effects on cell viability. These results demonstrate that TC2M holds promise as an effective photosensitizer for targeted tumor cell killing of MDA-MB-231 cells. For the different photodynamic performance of two PSs in cells, we speculate that the possible reasons are as follows: (i) the cellular uptake of TC1M and TC2M differ significantly, leading to a large disparity in phototoxicity. (ii) TC2M can accumulate in mitochondria whereas TC1M cannot, which enables TC2M to generate ROS specifically within mitochondria, thereby inducing cell death.^11^

To elucidate the mechanism underlying the photodynamic activity of TC2M, we monitored intracellular ROS generation using the non-fluorescent probe 2′,7′-dichlorodihydrofluorescein diacetate (DCFH-DA), which can be converted into strongly green-fluorescent 2′,7′-dichlorofluorescein (DCF) in the presence of ROS.^28^ As shown in Fig. 8c, MDA-MB-231 cells stained with TC2M exhibited bright green fluorescence upon white light irradiation, whereas no obvious fluorescence was observed in cells treated with TC2M without irradiation or in control groups with or without light exposure. These results indicate that TC2M can efficiently generate ROS under light irradiation. Furthermore, we performed live/dead cell staining to assess cell death. Live cells were stained green with calcein acetoxymethyl ester (calcein-AM), while dead cells were stained red with propidium iodide (PI).^29,30^ In contrast to the control groups, which showed predominantly green fluorescence, only the TC2M-treated group with white light irradiation exhibited substantial red fluorescence from dead cells (Fig. 8d). These findings collectively demonstrate that TC2M effectively sensitizes oxygen to generate ROS upon light irradiation, thereby inducing cell death.

## Conclusions

In summary, we have successfully constructed two mannose-functionalized glycol-nanoparticles for breast cancer cell-targeted NIR bioimaging and efficient PDT. Two carbazole-based PSs were designed with electronic “D–π–A” structures, exhibiting ICT-dominant photophysical properties, such as broad absorption bands, NIR fluorescence and large stokes-shifts. TC1 and TC2 possess outstanding ROS generation efficacies under light irradiation through type-I and II pathways. To improve the biocompatibility and cell targeting, hydrophilic mannose and PEG chains were introduced into PSs to form two glycol-nanoparticles, exhibiting small hydrodynamic diameters, uniform morphologies and still remarkable ROS generation capability. In MDA-MB-231 cells, mannose-functionalized glycol-nanoparticles TC1M and TC2M exhibited excellent biocompatibility and selective uptake *via* mannose receptor-mediated endocytosis. Notably, TC2M demonstrated potent photodynamic activity, reducing cell viability to 23.15% at 20 µM under light irradiation through efficient intracellular ROS generation. These findings highlight the potential of mannose-decorated glycol-nanoparticles as targeted theranostic agents for breast cancer diagnosis and treatment.

## Conflicts of interest

There are no conflicts to declare.

## Supplementary Material

RA-OLF-D6RA05981C-s001

## Data Availability

The authors declare that the main data supporting the findings of this study are contained within the paper and its associated supplementary information (SI). All other relevant data are available from the corresponding author upon reasonable requirements. Supplementary information is available. See DOI: https://doi.org/10.1039/d6ra05981c.
