# Supplementary material for: Construction of mannosylated glycol-nanoparticles for near-infrared bioimaging and photodynamic therapy targeting breast cancer cells
Source: RSC Adv. 2026 Aug 21;16(41):49286–93. doi: 10.1039/d6ra05981c (PMC13497337; doi:10.1039/d6ra05981c)
Supplement: Supplementary file 1 [file RA-016-D6RA05981C-s001.pdf]

Supplementary Information (SI) for RSC Advances.
This journal is © The Royal Society of Chemistry 2026

Construction of Mannosylated Glycol-Nanoparticles for Near-Infrared Bioimaging and Photodynamic Therapy Targeting Breast Cancer Cell

Yu Tang,†[a] Zhu-Ting Song,†[b] Lu-Lu Sun,†[c], Meng Sun,[b] Ming-Yu Wang,[b] Hai-Hao Han,*[a,c,d] Jin Gong,*[e] Lei Dong*[b,e] and Tan Ke*[f]

[a] School of Pharmacy, Anhui University of Chinese Medicine, Hefei 230031, China

[b] School of Life Science and Technology, Shandong Second Medical University, Weifang, P.R. China

[c] Shandong Laboratory of Yantai Drug Discovery, Bohai Rim Advanced Research Institute for Drug Discovery, Yantai 264117, China

[d] Molecular Imaging Center, Shanghai Institute of Materia Medica, Chinese Academy of Sciences, Shanghai 201203, China

[e] School of Pharmacy, Shandong Second Medical University, Weifang, P.R. China

[f] Educational Technology Center, The PLA General Hospital, Beijing 100853, China

* Corresponding Author

† Co-contribution author

Email: hanhaihao@simm.ac.cn (H.-H. H), leidong@sdsmu.edu.cn (L. D.), gongjin@sdsmu.edu.cn (J. G.), tanke123@sina.com (T. K.)

Table of Contents

1. Additional Figures 3

2. Experimental Section 16

3. Synthesis and characterization of probes 20

4. Original spectra of new compounds 29

5. References 38

1. Additional Figures

Table S1. Summary of reported mannose-based photosensitizers for tumor PDT

No.	Photosensitizer	λ_{abs} / λ_{em} (nm)	Modification strategy	Targeted cells	Reference
**This work**	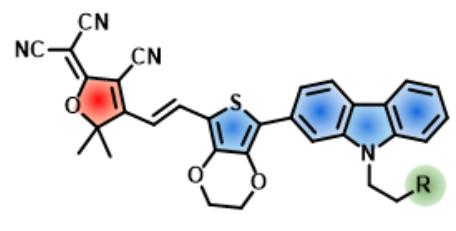 **TC2M**	**610 / 725**	**Chemical decoration**	**MDA-MB-231**	—
**1.**	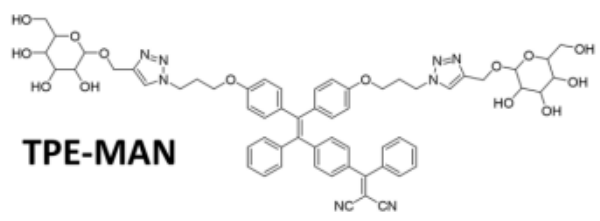	445 / 650	Chemical decoration	Tumor-associated macrophages	*Anal. Chem.* **2019**, *91*, 6836
**2.**	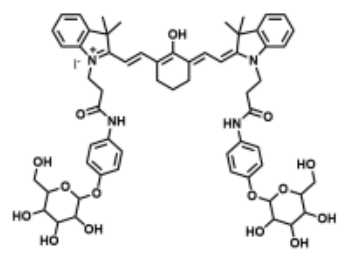	787 / 830	Chemical decoration	CT26 cells	*ACS Appl. Mater. Interfaces* **2026**, *18*, 1003
**3.**	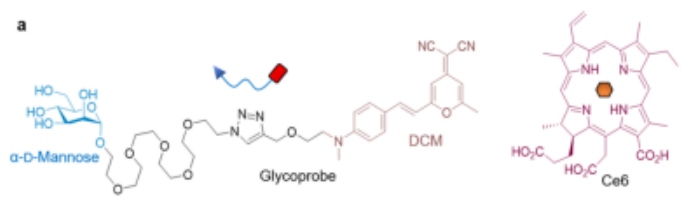	660 / 680	Supramolecular self-assembly	MDA-MB-231	*ACS Appl. Mater. Interfaces* **2019**, *11*, 22181
**4.**	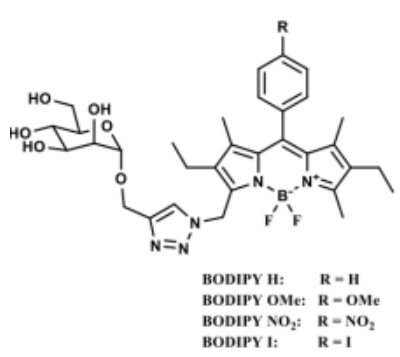	522 / 541	Chemical decoration	HeLa cells	*J. Mol. Struc.* **2025**, *1322*, 140433
**5.**	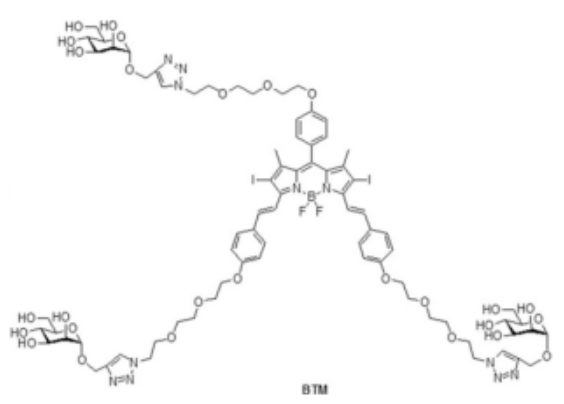	665 / 678	Chemical decoration	MDA-MB-231	*Chem. Eur. J.* **2017**, *23*, 14307
**6.**	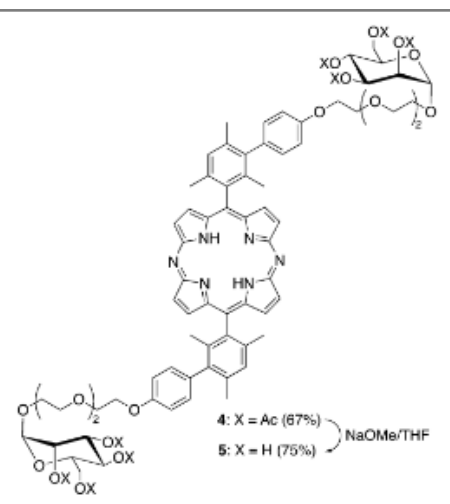	565 / 633	Chemical decoration	MDA-MB-231	*Org. Biomol. Chem.* **2022**, *20*, 8217
**7.**	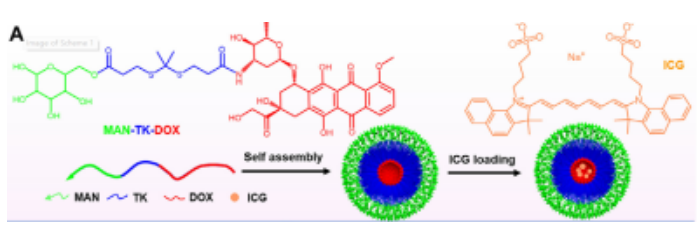	779 / 796	Supramolecular self-assembly	A549 cells	*Acta Biomaterialia* **2020**, *111*, 327
**8.**	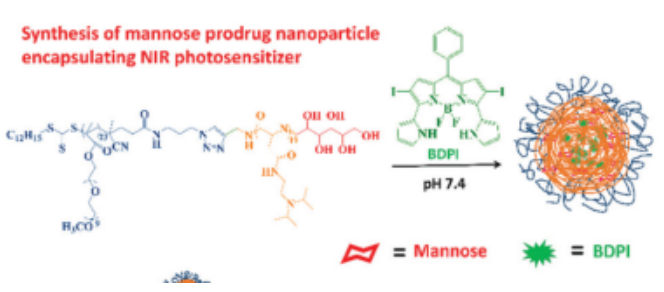	680 / 707	Supramolecular self-assembly	4T1 cells	*J. Mater. Chem. B* **2019**, *7*, 6770

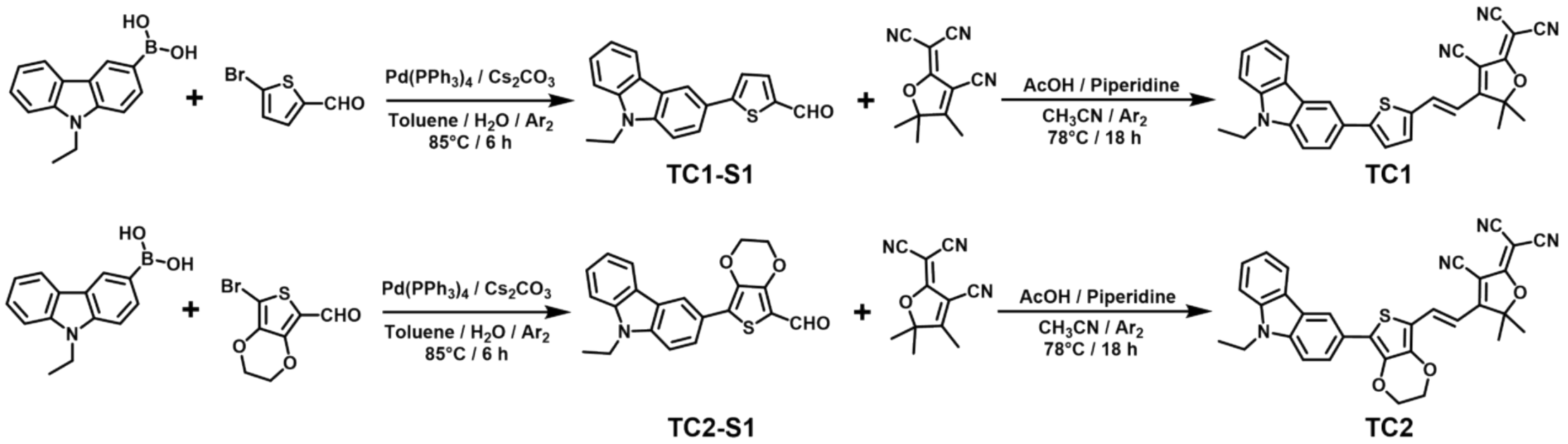

Scheme S1. Synthesis of PS **TC1** and **TC2**.

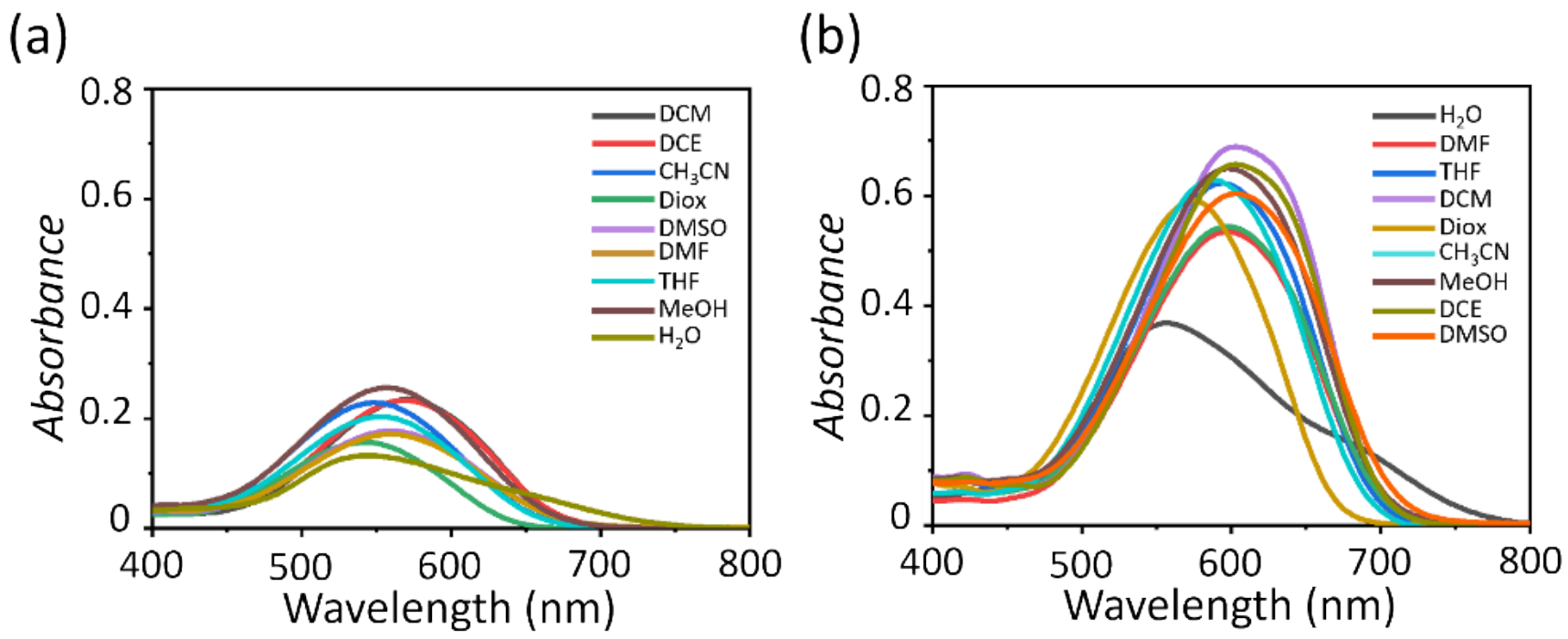

Figure S1. Absorption spectra of (a) **TC1** and (b) **TC2** (c = 10 μM) in different solvents, including dichloromethane (DCM), 1,2-dichloroethane (DCE), tetrahydrofuran (THF), 1,4-dioxane (diox), acetonitrile (CH_3CN), methanol (MeOH), dimethylsulfoxide (DMSO), dimethyl formamide (DMF) and H_2O.

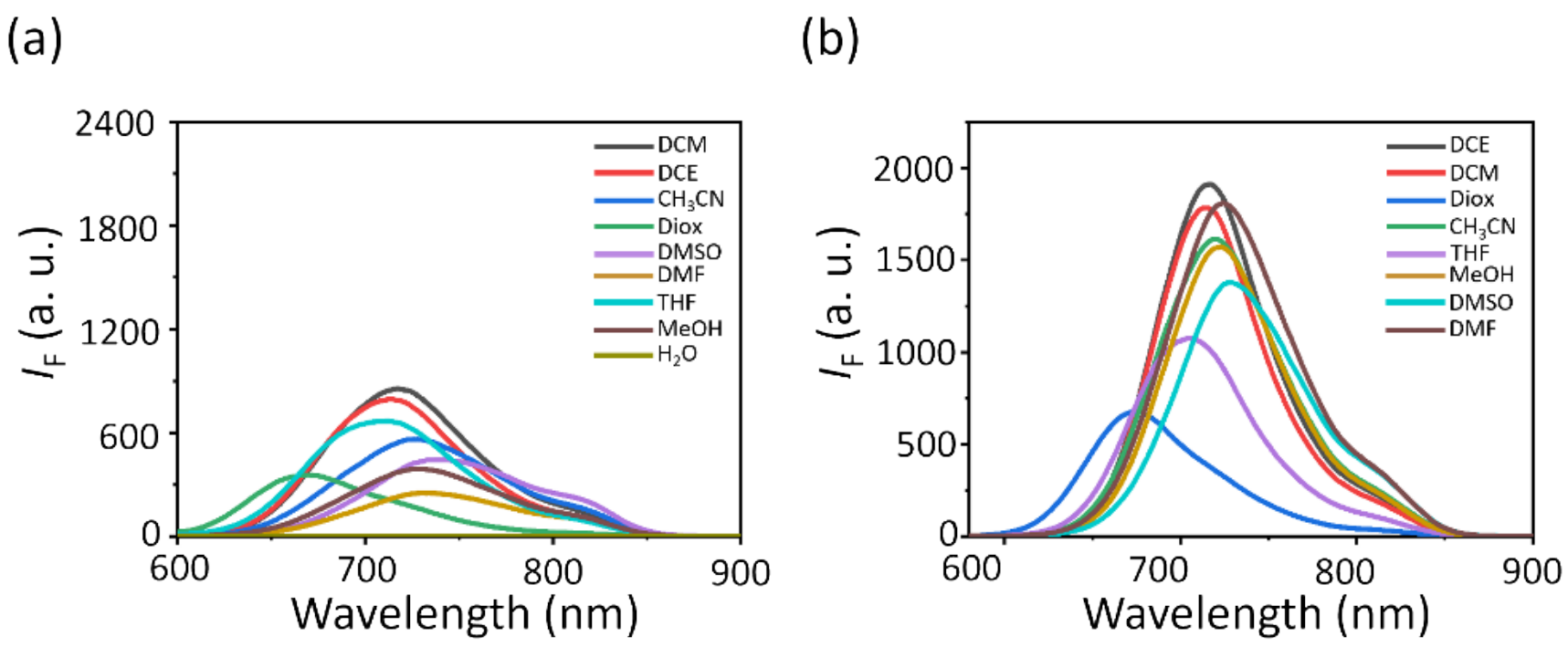

Figure S2. Fluorescence spectra of (a) **TC1** and (b) **TC2** in different solvents, including dichloromethane (DCM), 1,2-dichloroethane (DCE), tetrahydrofuran (THF), 1,4-dioxane (diox), acetonitrile (CH_3CN), methanol (MeOH), dimethylsulfoxide (DMSO), dimethyl formamide (DMF) and H_2O (c = 10 μM, Ex = 530 nm).

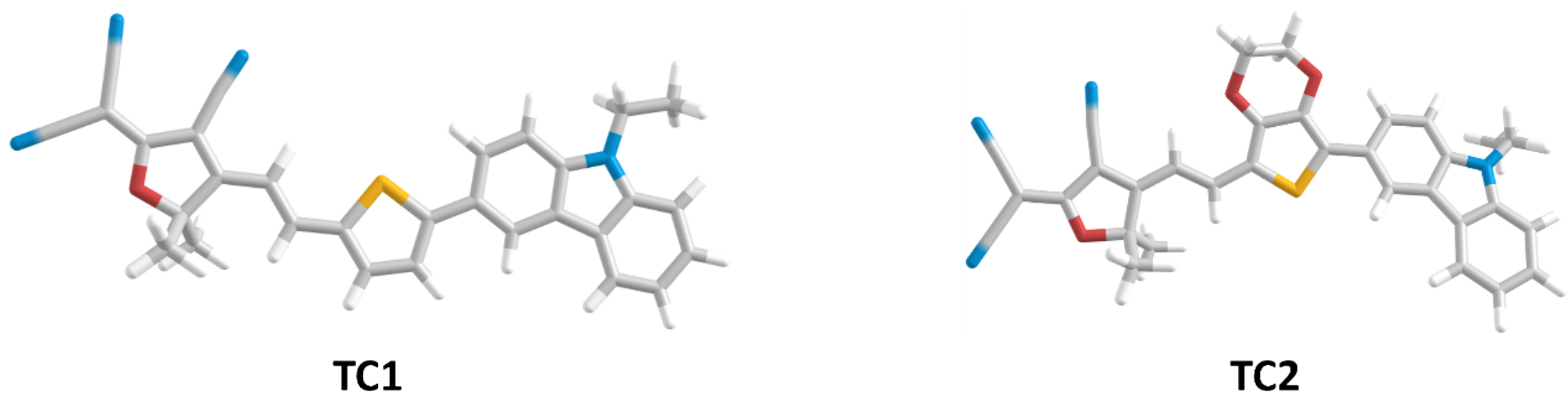

Figure S3. DFT calculated the optimal molecular conformations of **TC1** and **TC2**.

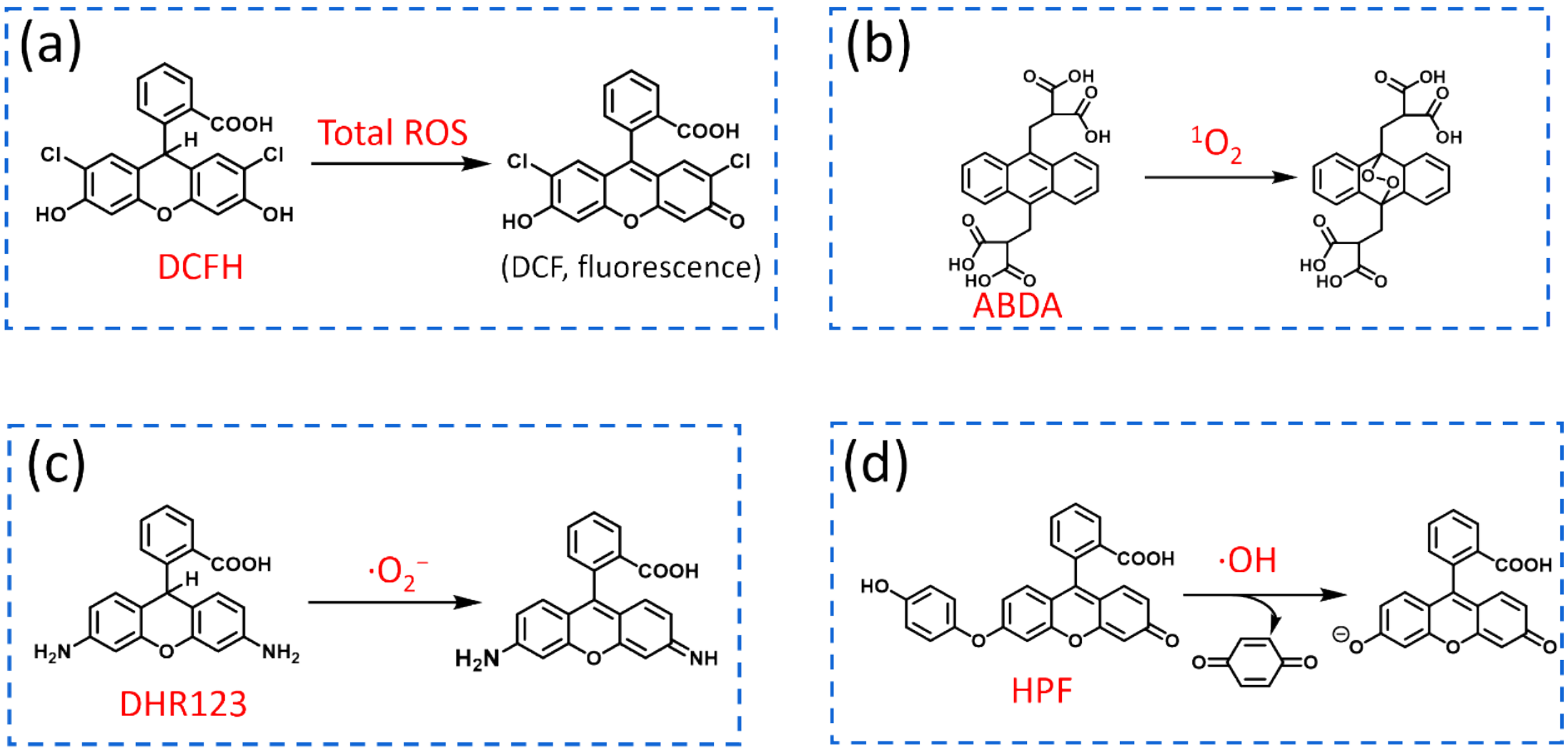

Figure S4. Mechanisms of ROS indicators (a) DCFH, (b) ABDA, (c) DHR123 and (d) HPF responding to the total ROS, singlet oxygen, superoxide radicals and hydroxy radicals, respectively.

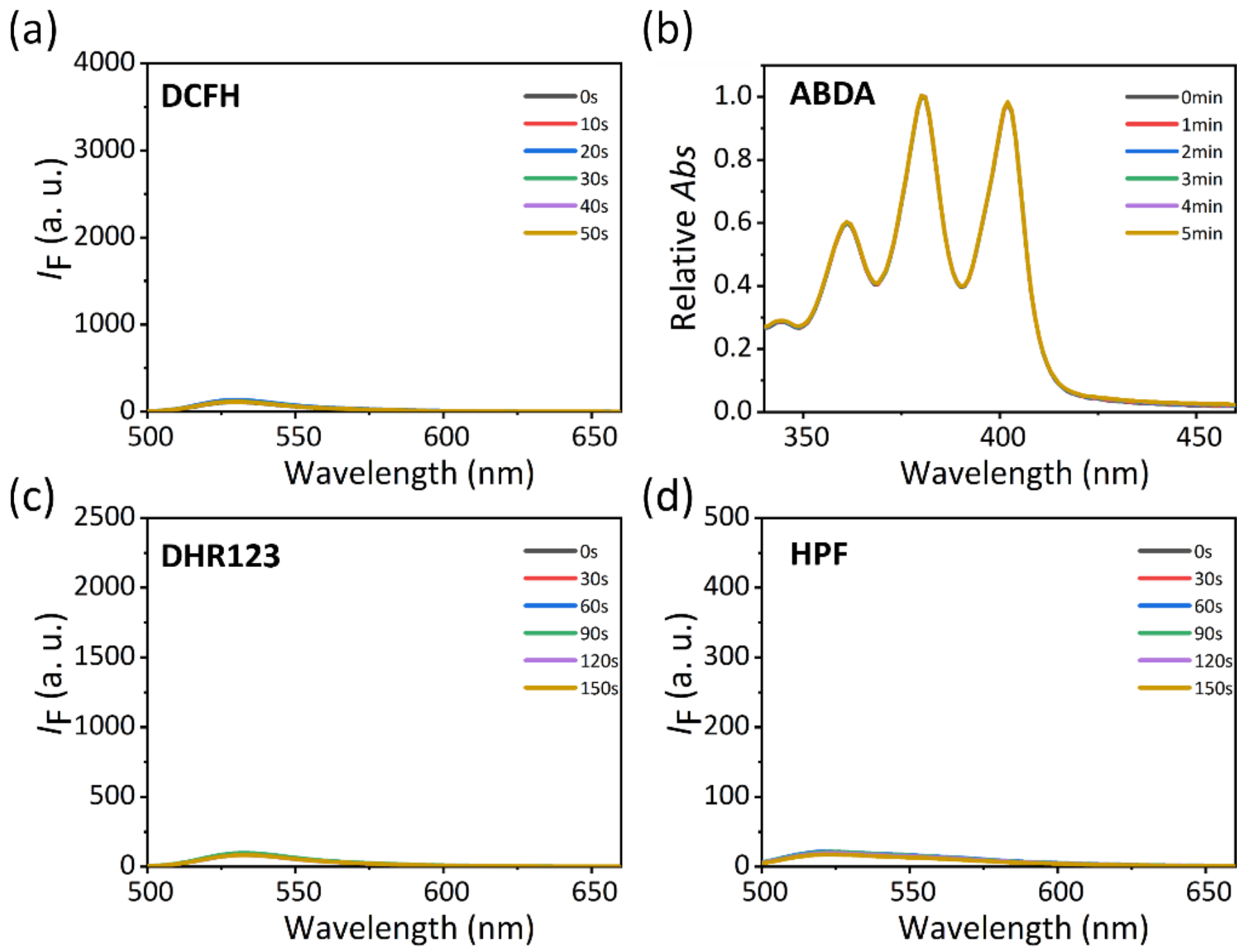

Figure S5. (a) Fluorescence spectra of DCFH in PBS under 590 nm light irradiation for 50 s (interval 10 s). (b) Relative absorption of ABDA in PBS under 590 nm light irradiation for 5 min (interval 1 min). (c) Fluorescence spectra of DHR123 in PBS under 590 nm light irradiation for 150 s (interval 30 s). (d) Fluorescence spectra of HPF in PBS under 590 nm light irradiation for 150 s (interval 30 s, Ex (DCFH, DHR123 and HPF) = 470 nm, P = 40 mW cm^{-2}).

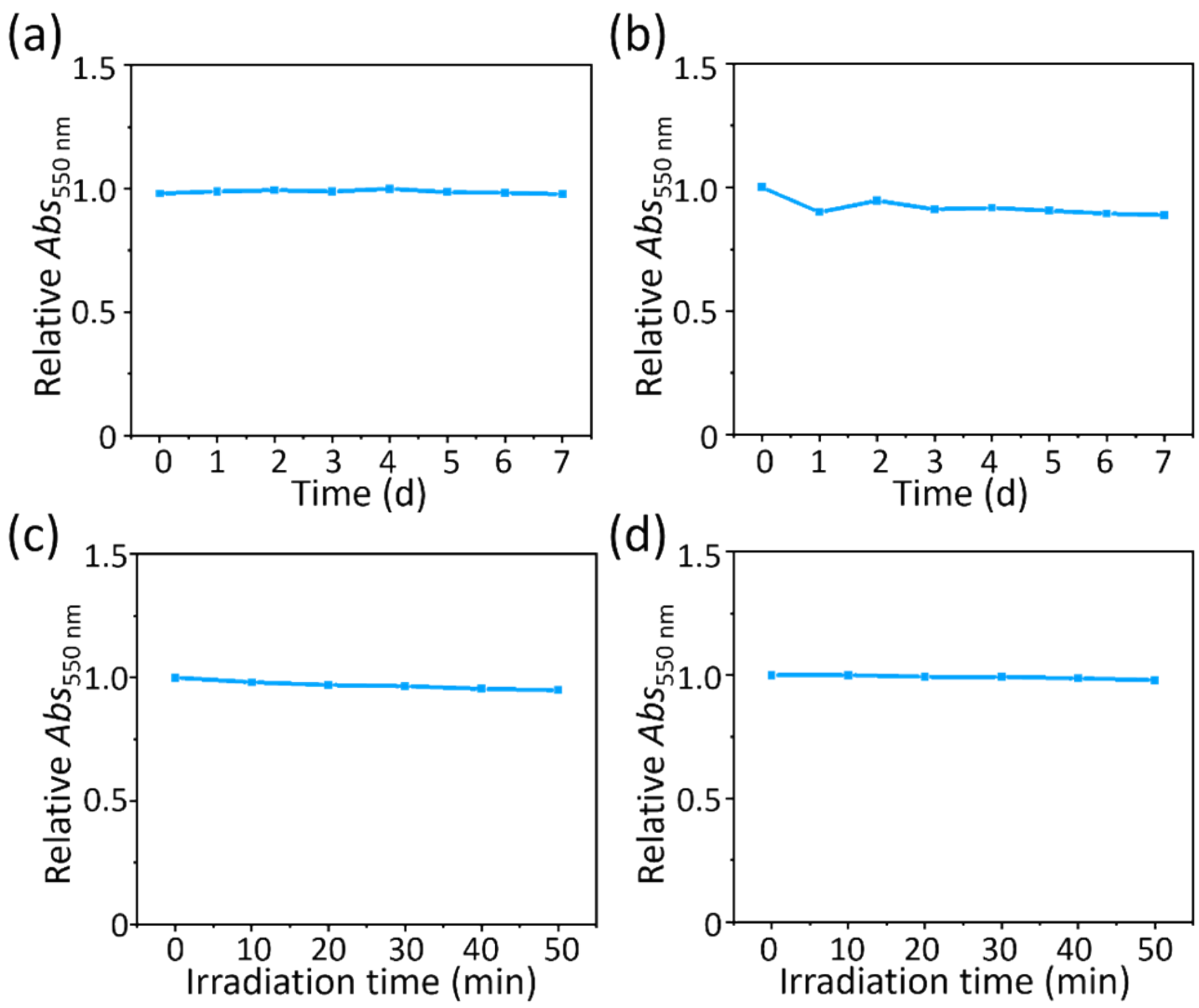

Figure S6. Plotting relative absorption intensity at 550 nm of (a) **TC1** and (b) **TC2** in PBS buffer for 7 days. Plotting relative absorption intensity at 550 nm of (c) **TC1** and (d) **TC2** in PBS buffer under 590 nm light irradiation within 50 min (P = 40 mW cm^{-2}).

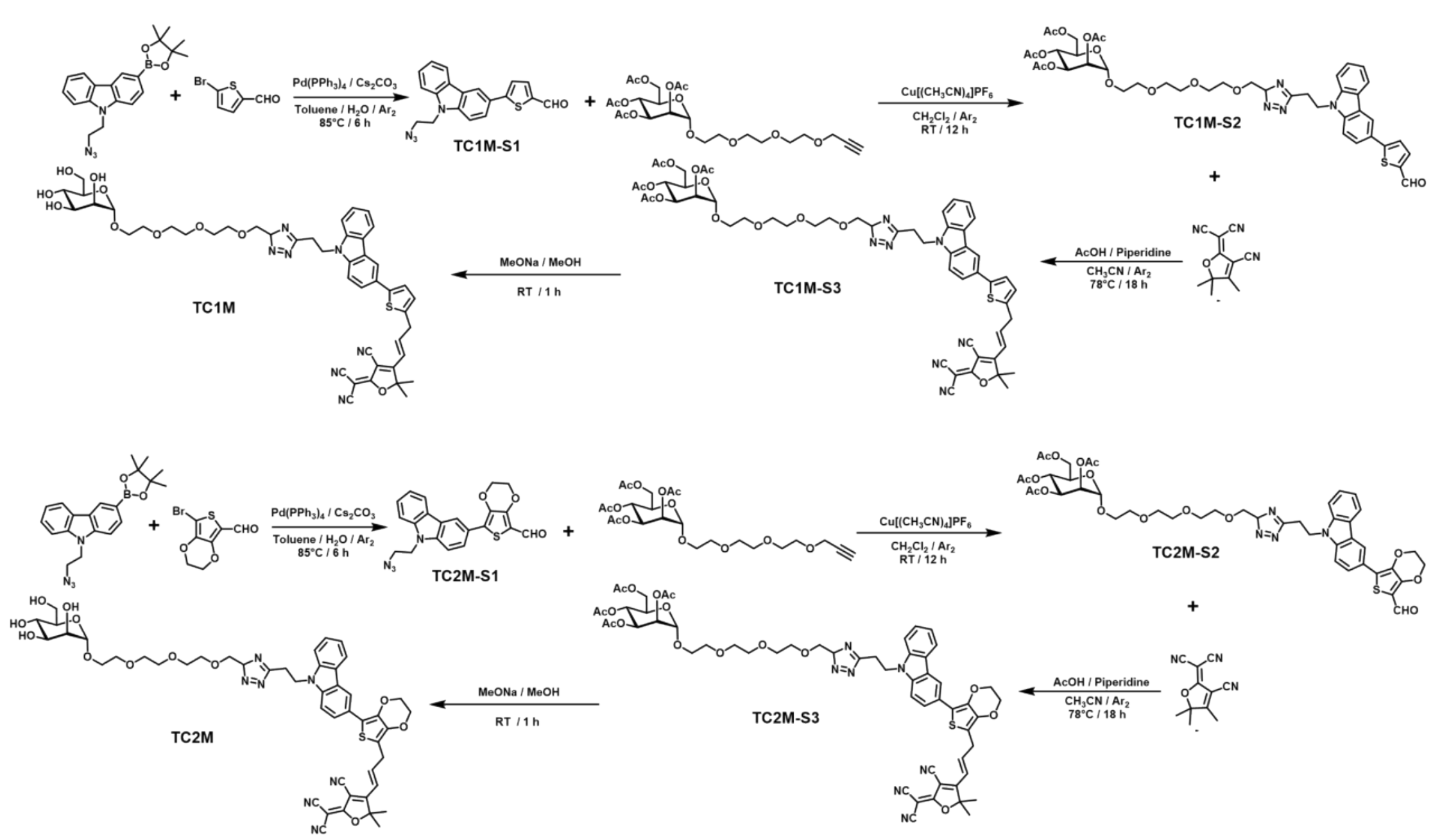

Scheme S2. Synthesis of PS **TC1M** and **TC2M**.

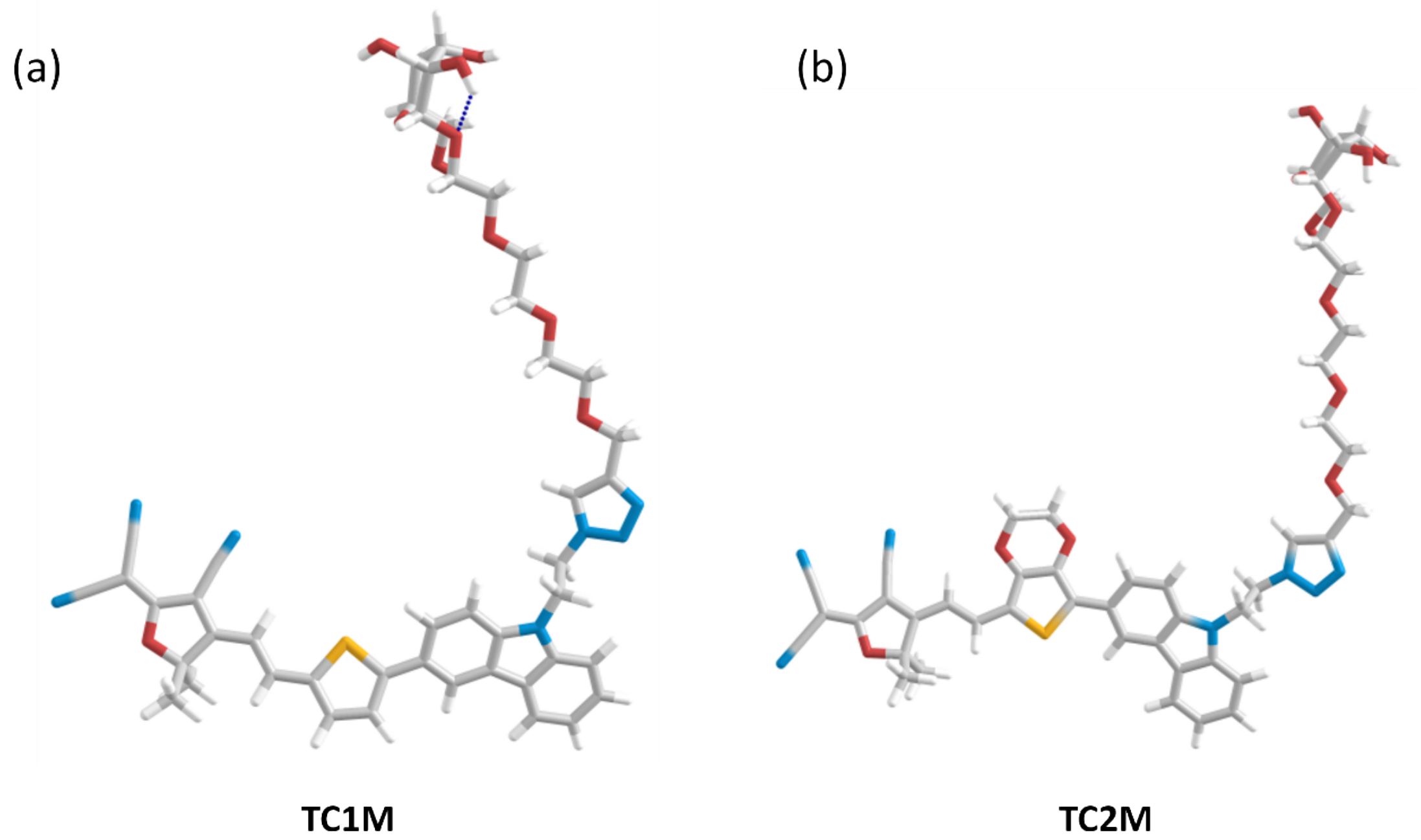

Figure S7. DFT calculated the optimal molecular conformations of glycol-PSs (a) **TC1M** and (b) **TC2M**.

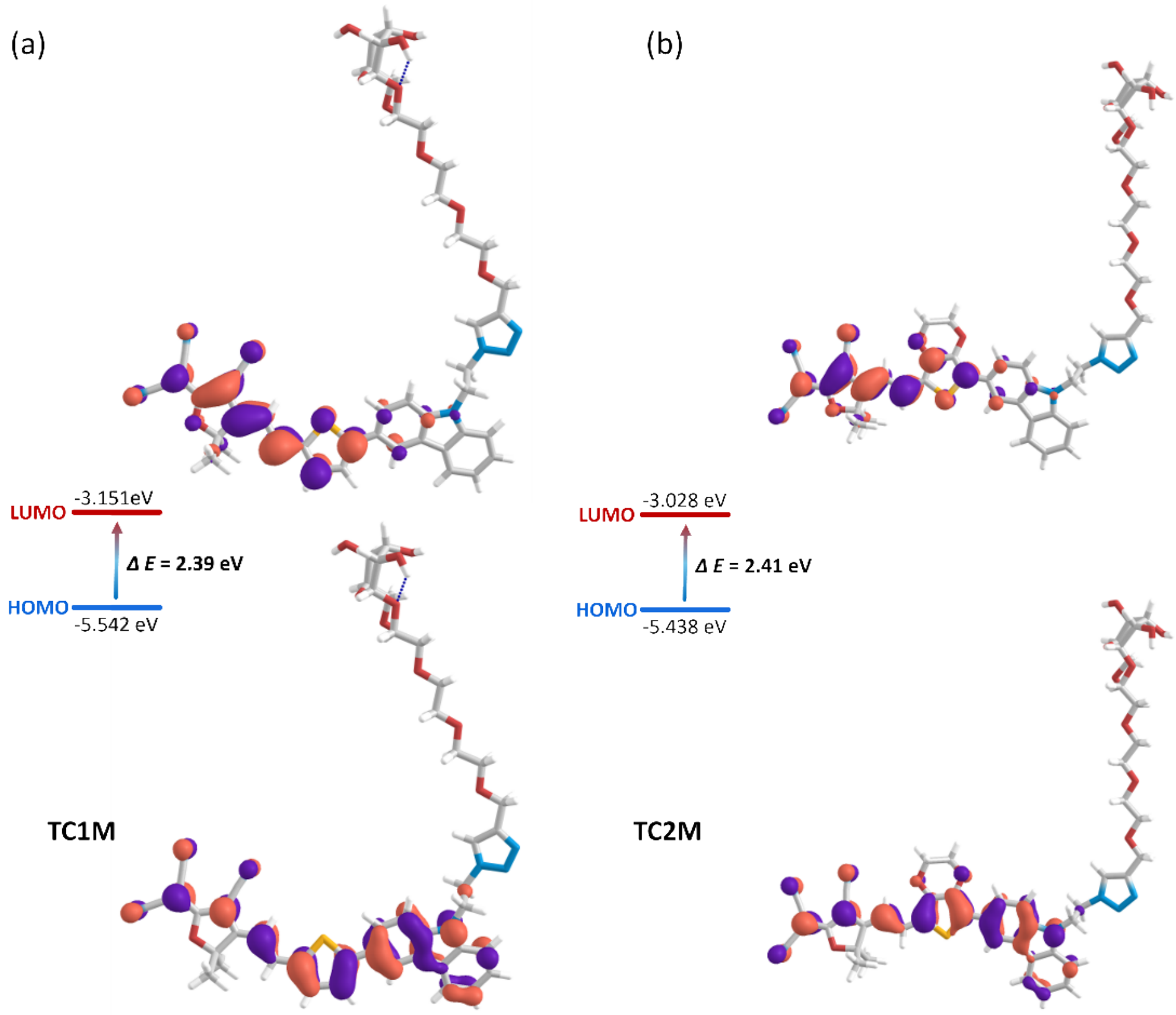

Figure S8. DFT calculated the HOMO-LUMO distribution of (a) **TC1M** and (b) **TC2M**.

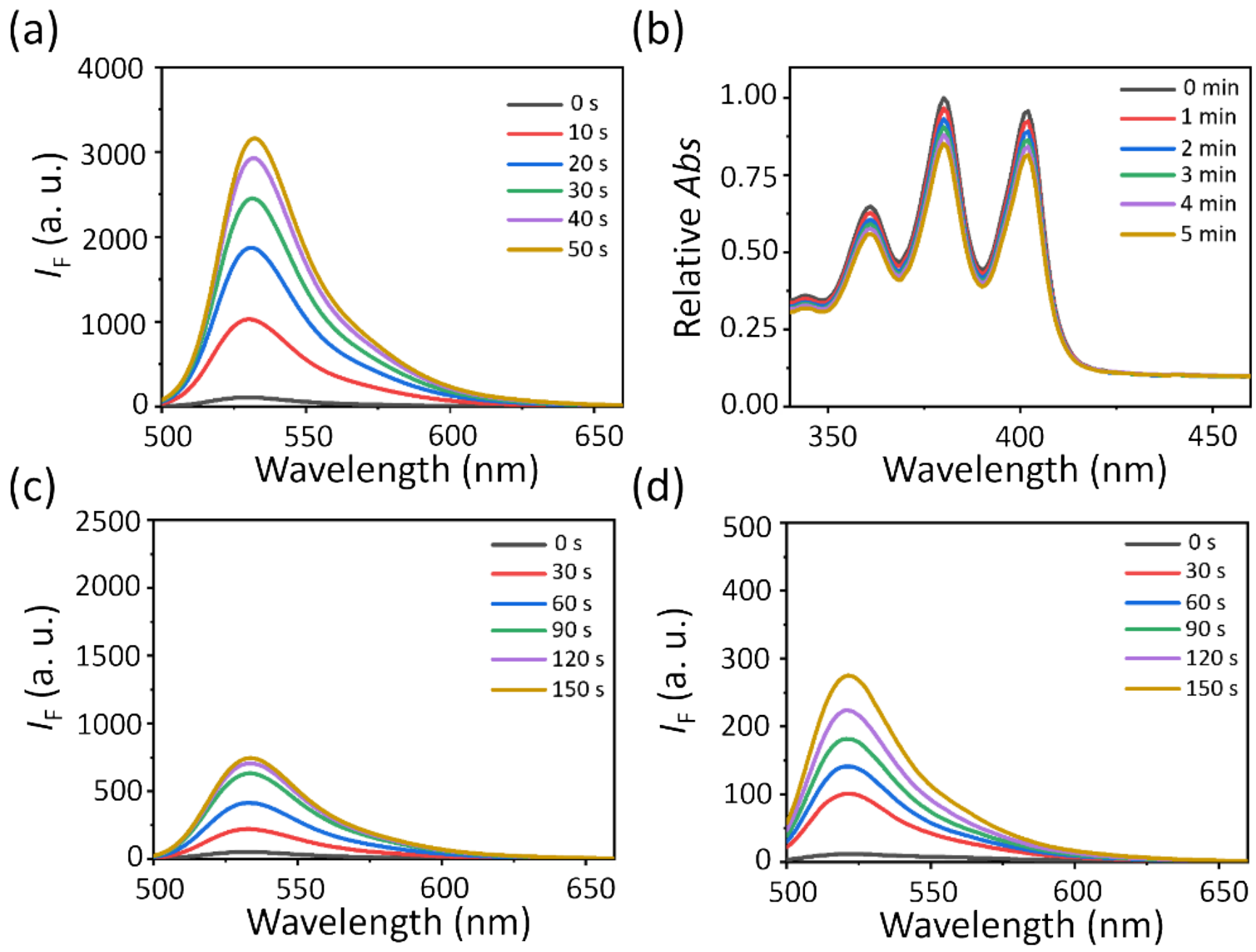

Figure S9. (a) Fluorescence spectra of DCFH containing **TC1M** (c = 10 μM) in PBS under 590 nm light irradiation for 50 s (interval 10 s). (b) Relative absorption of ABDA containing **TC1M** (c = 10 μM) in PBS under 590 nm light irradiation for 5 min (interval 1 min). (c) Fluorescence spectra of DHR123 containing **TC1M** (c = 10 μM) in PBS under 590 nm light irradiation for 150 s (interval 30 s). (d) Fluorescence spectra of HPF containing **TC1M** (c = 10 μM) in PBS under 590 nm light irradiation for 150 s (interval 30 s, Ex (DCFH, DHR123 and HPF) = 470 nm, *P* = 40 mW cm^{-2}).

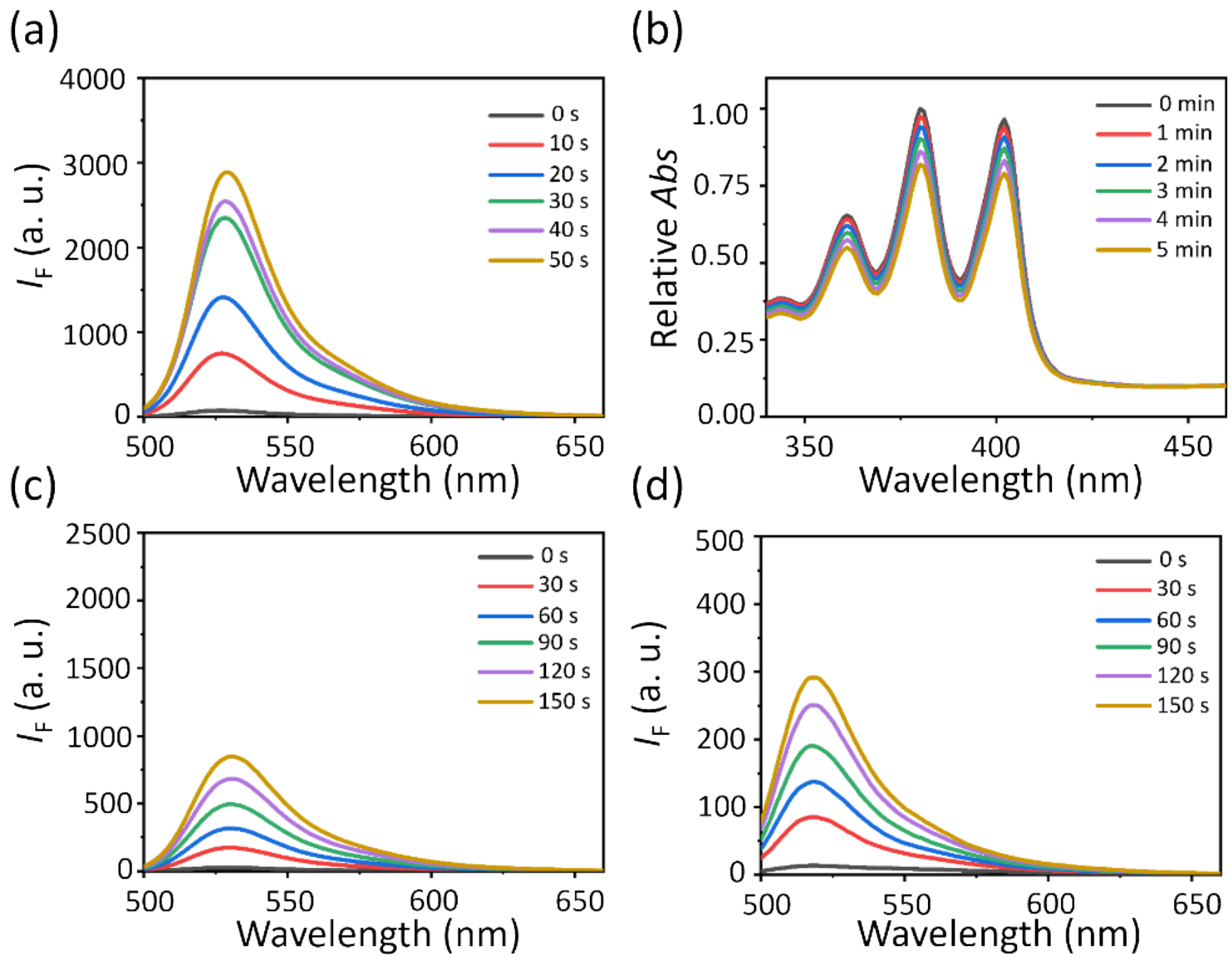

Figure S10. (a) Fluorescence spectra of DCFH containing **TC2M** (c = 10 μM) in PBS under 590 nm light irradiation for 50 s (interval 10 s). (b) Relative absorption of ABDA containing **TC2M** (c = 10 μM) in PBS under 590 nm light irradiation for 5 min (interval 1 min). (c) Fluorescence spectra of DHR123 containing **TC2M** (c = 10 μM) in PBS under 590 nm light irradiation for 150 s (interval 30 s). (d) Fluorescence spectra of HPF containing **TC2M** (c = 10 μM) in PBS under 590 nm light irradiation for 150 s (interval 30 s, Ex (DCFH, DHR123 and HPF) = 470 nm, P = 40 mW cm^{-2}).

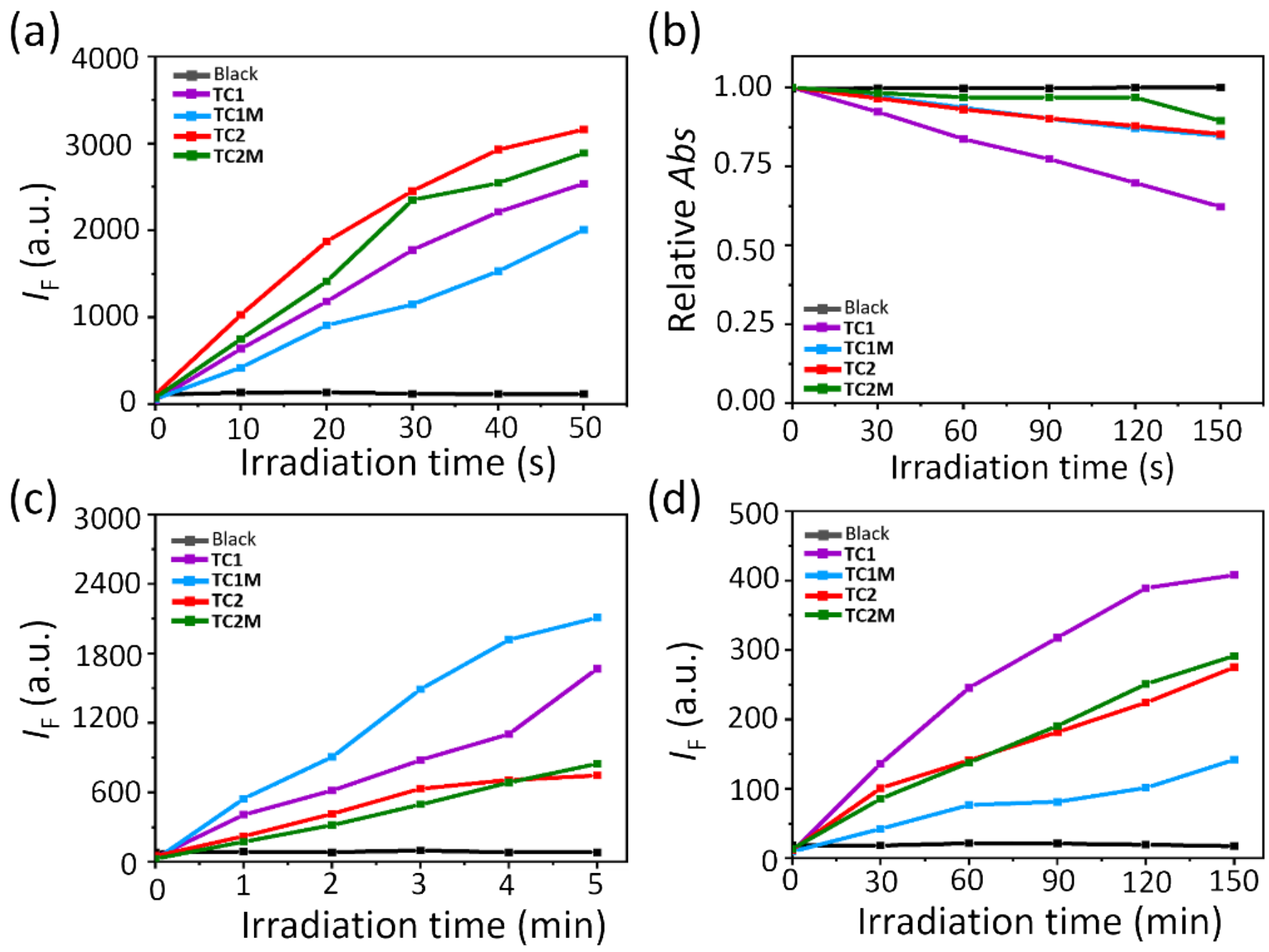

Figure S11. (a) Plotting fluorescence maxima of DCFH only or containing **TC1**, **TC2**, **TC1M** and **TC2M** (c = 10 μM) in PBS under 590 nm light irradiation for 50 s (interval 10 s). (b) Plotting relative absorption maxima of ABDA only or containing **TC1**, **TC2**, **TC1M** and **TC2M** (c = 10 μM) in PBS under 590 nm light irradiation for 5 min (interval 1 min). (c) Plotting fluorescence maxima of DHR123 only or containing **TC1**, **TC2**, **TC1M** and **TC2M** (c = 10 μM) in PBS under 590 nm light irradiation for 150 s (interval 30 s). (d) Plotting fluorescence maxima of HPF only or containing **TC1**, **TC2**, **TC1M** and **TC2M** (c = 10 μM) in PBS under 590 nm light irradiation for 150 s (interval 30 s, Ex (DCFH, DHR123 and HPF) = 470 nm, *P* = 40 mW cm^{-2}).

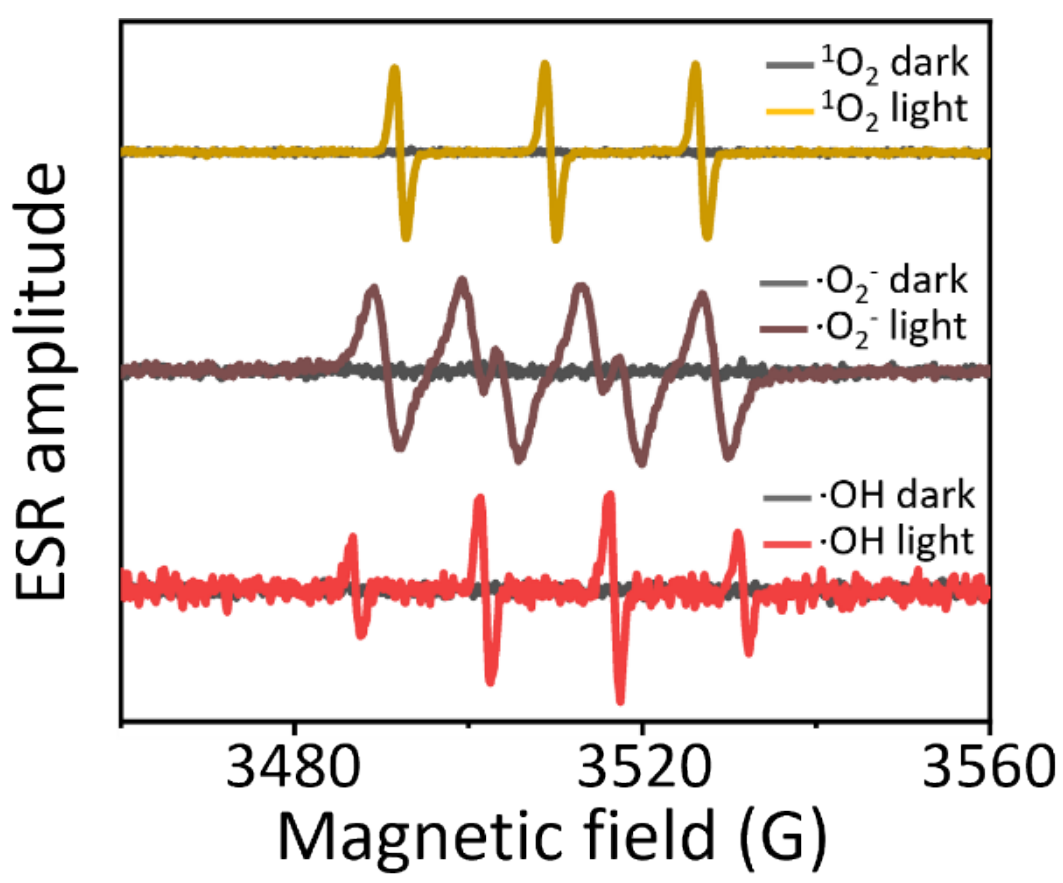

Figure S12. Electron spin resonance (ESR) analysis in the presence of **TC2M** and different spin-trap agents before and after white light irradiation (*P* = 40 mW cm^{-2}, 10 min).

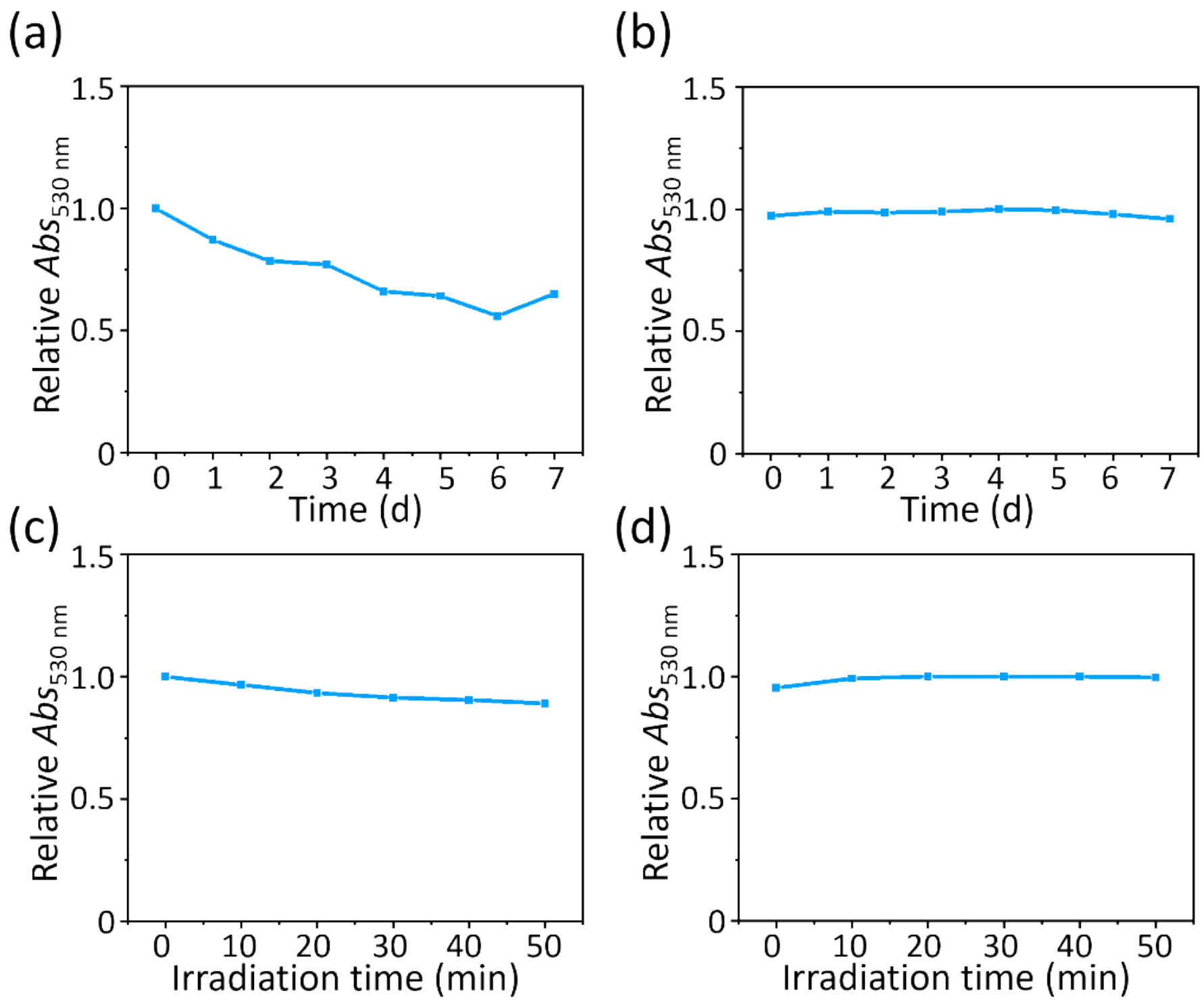

Figure S13. Plotting relative absorption intensity at 530 nm of (a) **TC1M** and (b) **TC2M** in PBS buffer for 7 days. Plotting relative absorption intensity at 530 nm of (c) **TC1M** and (d) **TC2M** in PBS buffer under 590 nm light irradiation within 50 min (P = 40 mW cm^{-2}).

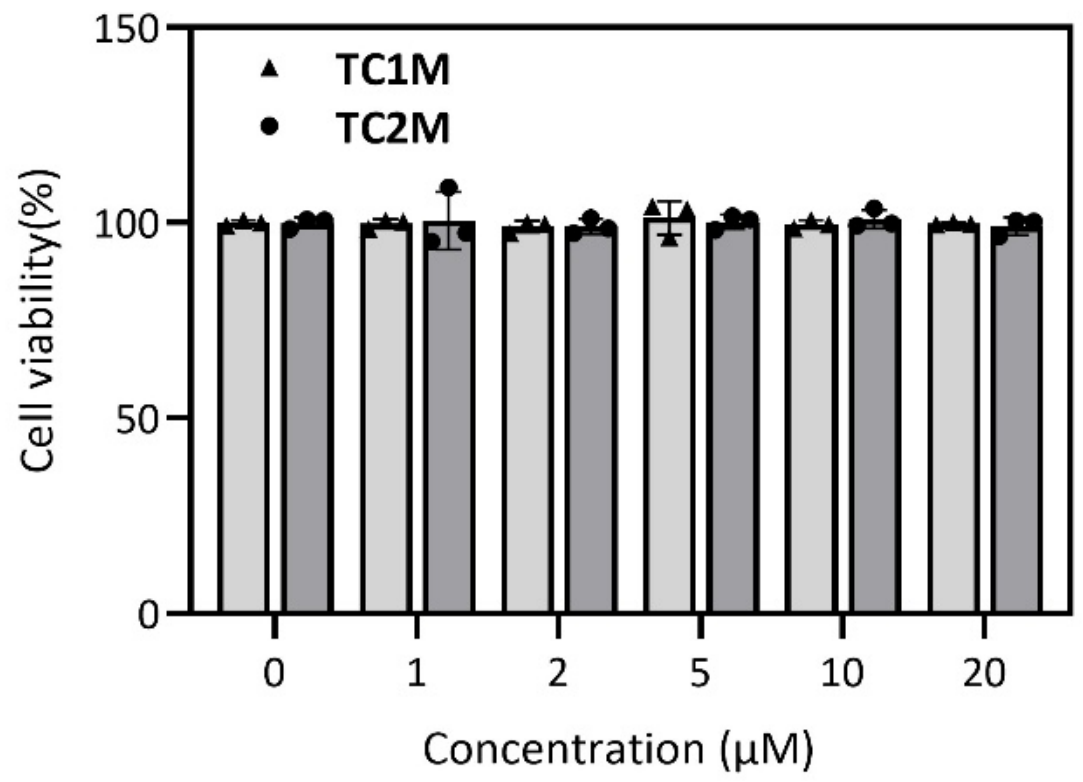

Figure S14. Cell viability of MDA-MB-231 cells with different concentrations (0-20 μM) of **TC1M** and **TC2M** for 24 h. Error bars: mean ± SD (n=3).

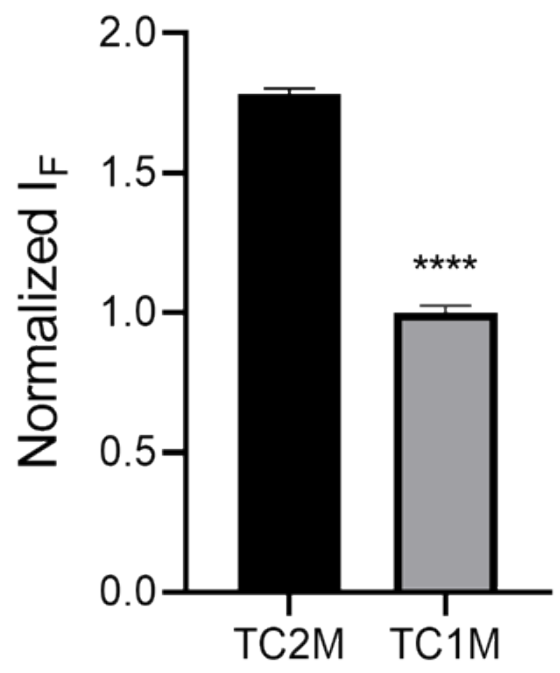

Figure S15. Fluorescence quantification of MDA-MB-231 cells incubated with **TC1M** and **TC2M** (1 μM) for 4 h. $\lambda_{ex}/\lambda_{em}$ = 561/650–750 nm for PSs. Data are represented as mean ± SD of three parallel experiments and analyzed by t-test. Statistical analysis: ****$p < 0.0001$.

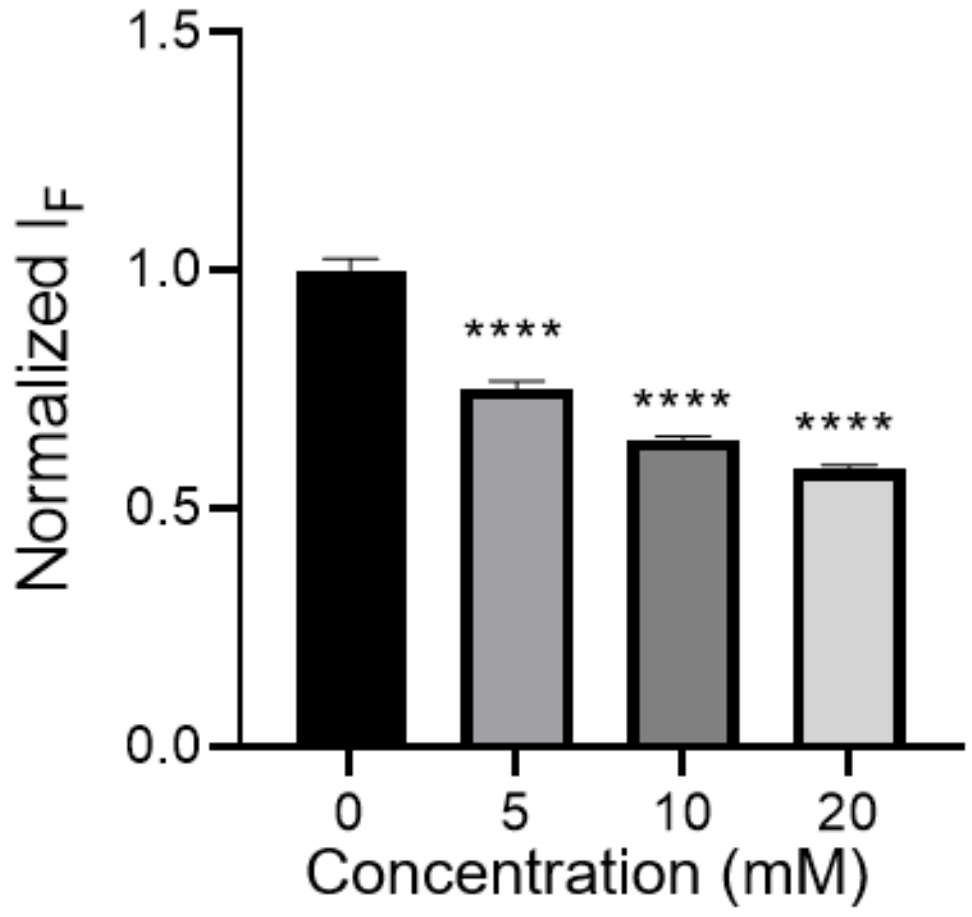

Figure S16. Fluorescence quantification of MDA-MB-231 cells co-incubating with **TC2M** (1 μM) and different concentrations (0, 5, 10 and 20 mM) of mannose for 4 h. $\lambda_{ex}/\lambda_{em}$ = 561/650–750 nm for PSs. Data are represented as mean ± SD of three parallel experiments. Statistical significance was assessed using one-way ANOVA followed by Dunnett's multiple-comparisons test, with each mannose-treated group compared with the 0 mM mannose group. ****$p < 0.0001$.

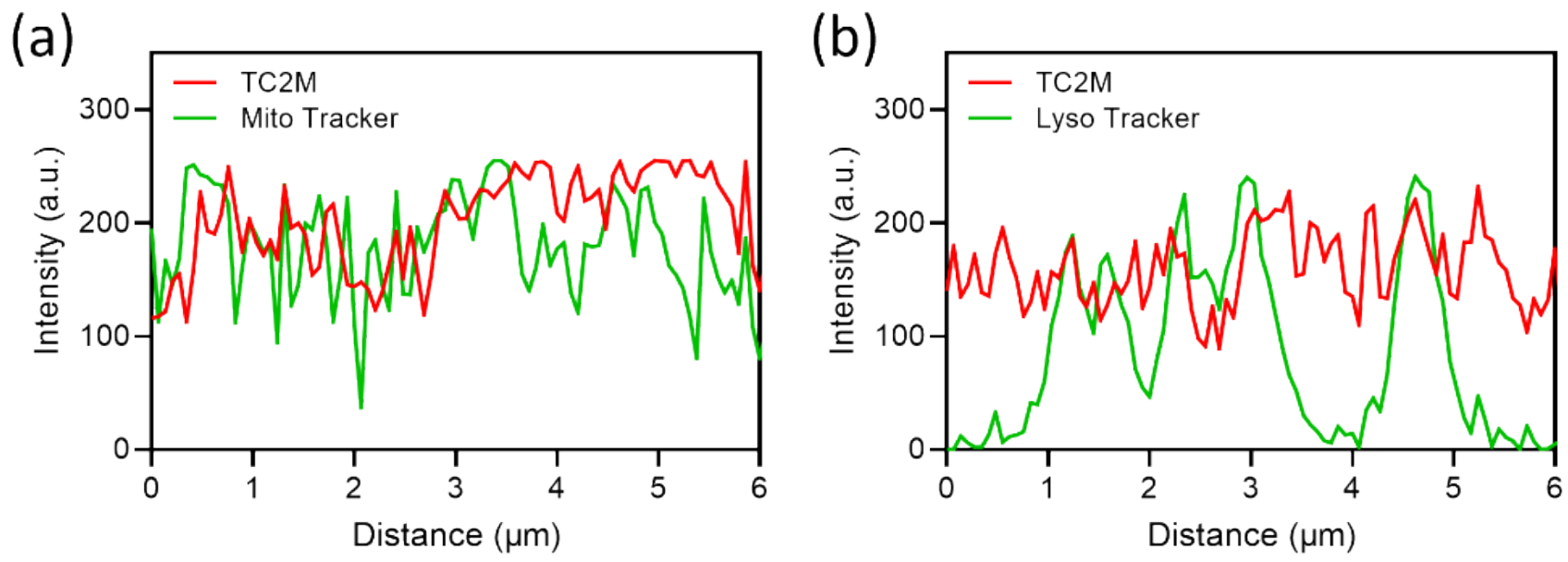

Figure S17. Fluorescence intensity profiles of **TC2M** and the organelle trackers along the white lines indicated in the corresponding merged images in Figure 7c: (a) **TC2M** and MitoTracker and (b) **TC2M** and LysoTracker. The red and green curves represent the fluorescence intensities of **TC2M** and the corresponding organelle tracker, respectively.

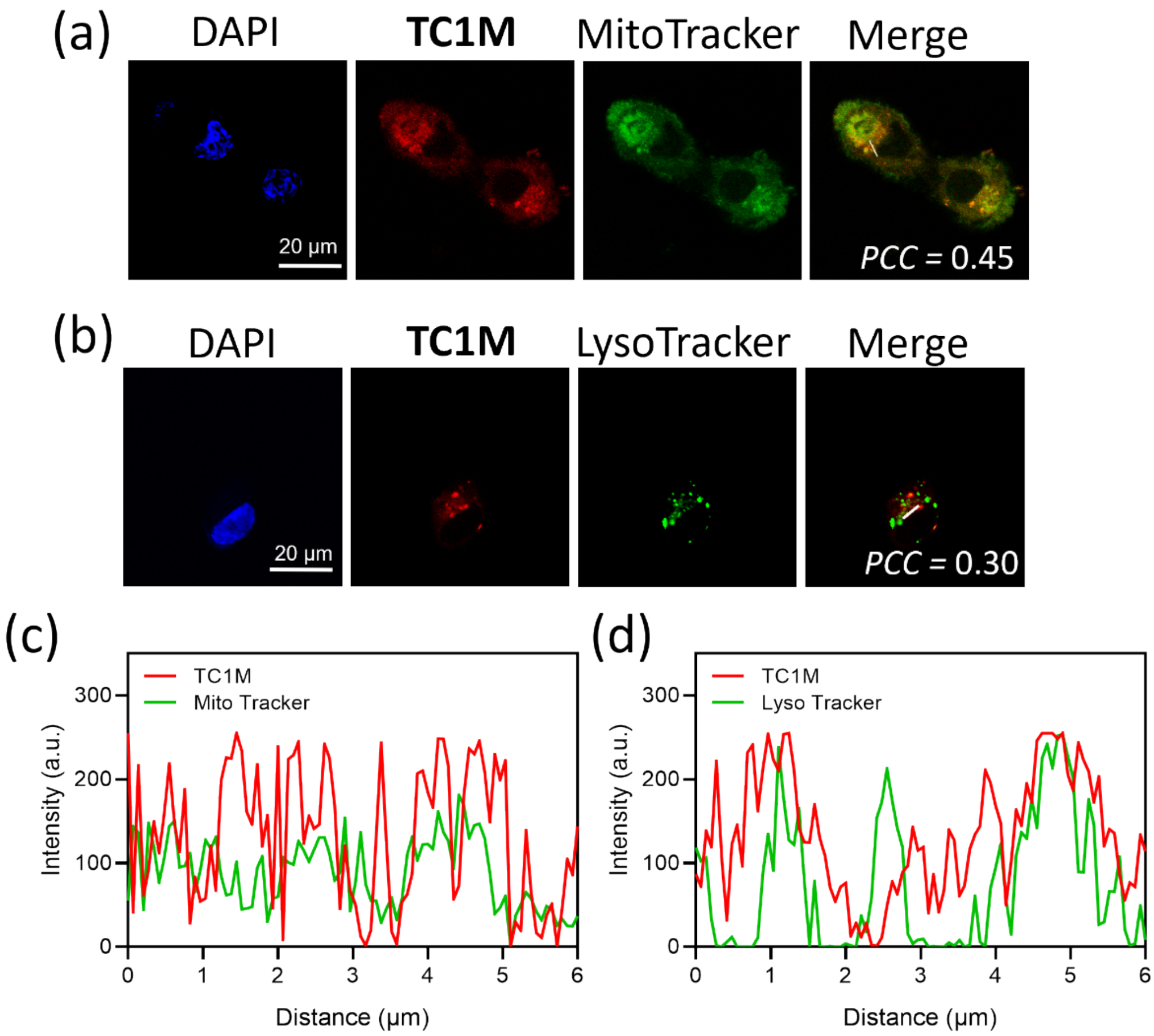

Figure S18. (a,b) Fluorescence colocalization imaging and (c, d) quantification of **TC1M** with (a, c) MitoTracker (a mitochondria tracker, 100 nM) and (b, d) LysoTracker (a lysosome tracker, 100 nM) in MDA-MB-231 cells.

2. Experimental Section

Materials and Reagents

The various reagents and organic solvents used in the experiments were of analytical grade and were purchased from inno-chem Ltd. All reagents and organic solvents required no further purification and were used directly unless otherwise stated. The water used in the experiments was double distilled water. The progress of the reaction was monitored by observing thin layer chromatography under an ultraviolet lamp. The thin layer chromatography used in the experiments and the silica gel (200-400 mesh) used in the column chromatography were purchased from Qingdao Ocean Chemicals. Absorption spectra were measured on UV-2550 UV/Vis spectrophotometer (Hitachi Japan), and fluorescence emission spectra were measured on F-4700fluorescence spectrophotometer (Hitachi Japan). ^{1}H NMR and ^{13}C NMR spectra were collected by Bruker spectrometer. Chemical shift (δ) values are in ppm and tetramethylsilane is used as an internal standard. High resolution mass spectra (HRMS) were measured by Agilent 6510 Q-TOF LC/MS instrument (Agilent Technologies, Palo Alto, CA) equipped with an electrospray ionization (ESI) source. Electron paramagnetic resonance (ESR) were measured by Bruker A300. Dynamic light scattering (DLS) investigations were carried out with a Malvern Zetasizer Pro. Transmission Electron Microscope (TEM) were recorded by Hitachi HT-7700. Irradiation was performed by using a LED light (white or 590 nm light, PLS-LED 100, Perfect Light, Beijing, China). Cell fluorescence images were collected by using Olympus FV3000 laser scanning confocal microscopy.

UV-Vis and fluorescence spectroscopy

The UV-visible spectra were analyzed after correcting for the solvent background by subtracting the spectra of pure solvent measured under identical conditions. The fluorescent measurements were carried out at room temperature. The excitation slit width was 2.5 nm, emission slit width was 5 nm, and PMT voltage was 700V. The relative absorption (*Abs*) and relative emission intensity (I_F) were normalized by setting

the maximum values of the spectra to 1, with all other values scaled proportionally within the [0,1] range.

Calculation of optimized geometries and HOMO-LUMO

Gaussian 16 program was used to perform density functional theory (DFT) calculations (B3LYP/6-31G(d,p) em=gd3bj) of **TC1** and **TC2** in the gas phase[1]. The high-performance computing server was provided by Beijing Super Cloud Computing Center, Beijing, China.

Calculation of hole-electron distribution in excited state

Gaussian 16 program calculated the hole-electron distributions in S_1 excited state by using density functional theory at B3LYP/6-31G(d,p) em=gd3bj level of theory in the water. The software program Multiwfn was used to analyze the calculation results and generate graphs of hole–electron distribution[2-4]. The high-performance computing server was provided by Beijing Super Cloud Computing Center, Beijing, China[1-4].

Dispersion of PSs in aqueous buffer

Compounds **TC1** and **TC2** (0.1 μmol) were well-dissolved in DMSO (20 μL). The resulting solution was added 9980 μL deionized (DI) water or PBS buffer (1×, pH 7.4) and then dispersed under ultrasound in ice bath for 10 min to form the resulting aqueous dispersion (100 μM) in water or PBS buffer, which was subsequently diluted to low concentration for spectral and ROS evaluations.

Preparation of glycol-nanoparticles

Compound **TC1M** and **TC2M** (0.1 μmol) were well-dissolved in DMSO (20 μL). The resulting solution was added 9980 μL deionized (DI) water or PBS buffer (1×, pH 7.4) and then dispersed under ultrasound in ice bath for 10 min to form the resulting aqueous dispersion (100 μM) in water or PBS buffer, which was subsequently diluted to low concentration for spectral and ROS evaluations.

Detection of ROS production by DCFH in solution

Commercial probe 2',7'-dichlorofluorescein (DCFH) was used as ROS detector. 10 μM of determinands were dissolved in 2 mL DMF containing 20 μM of DCFH. 10 μM of determinands (PSs or glycol-nanoparticles) was prepared in 2 mL PBS buffer (1×, pH 7.4) containing 10 μM of DCFH. The dispersion was then placed in a cuvette and irradiated with a 590 nm LED light at 40 mW cm^{-2} for 50 s. The fluorescence change of sample at 525 nm was recorded under excitation at 470 nm.

Detection of $^1O_2$ production in solution

Compound 9,10-anthracenediyl-bis(methylene)-dimalonic acid (ABDA) was used as indicator for detection of $^1O_2$ in solution. When $^1O_2$ is generated in the system, the ABDA will be oxidized, and the absorption decreases at 380 nm. 10 μM of determinands (PSs or glycol-nanoparticles) was prepared in 2 mL PBS buffer (1×, pH 7.4) containing 50 μM of ABDA. The dispersion was then placed in a cuvette and irradiated with a 590 nm LED light at 40 mW cm^{-2} for 5 min. The absorption decrease of sample at 380 nm was recorded by the UV-Vis absorption spectrophotometer.

Detection of $•O_2^-$ production with DHR123 in solution

Commercial probe dihydrorhodamine 123 (DHR123) was used as $•O_2^-$ detector. When $•O_2^-$ is generated in the system, DHR123 can be oxidized to emit bright fluorescence at *ca.* 530 nm. 10 μM of determinands (PSs or glycol-nanoparticles) was prepared in 2 mL PBS buffer (1×, pH 7.4) containing 10 μM of DHR123. The mixture was then placed in a cuvette and irradiated with a 590 nm LED light at 40 mW cm^{-2} for 150 s. The fluorescence change of sample at 530 nm was recorded under excitation at 470 nm.

Detection of •OH production with HPF in solution

Commercial probe hydroxyphenyl fluorescein (HPF) was used as •OH detector. When •OH is generated in the system, HPF can be oxidized to emit bright fluorescence at *ca.* 520 nm. 10 μM of determinand (PSs or glycol-nanoparticles) was prepared in 2 mL PBS buffer (1×, pH 7.4) containing 10 μM of HPF. The mixture was then placed in a

cuvette and irradiated with a 590 nm LED light at 40 mW cm^{-2} for 150s. The fluorescence change of sample at 520 nm was recorded under excitation at 470 nm.

Stability of glycol-nanoparticles at room temperature

The prefabricated PSs or glycol-nanoparticles (200 μL, 100 μM) were diluted in PBS buffer (pH 7.4, 1800 μL) to afford the diluted concentration (10 μM), and placed at room temperature. Absorption spectra were determined within 7 days (interval 1 d).

Photostability of glycol-nanoparticles

The dispersion of PSs or glycol-nanoparticles (200 μL, 100 μM) was diluted in PBS buffer (pH 7.4, 1800 μL) to afford the diluted concentration (10 μM). The resulting diluted PSs dispersion in PBS buffer was exposed under 590 nm LED light irradiation (P = 40 mW cm^{-2}) during 50 min. Absorption spectra were collected within 50 min (interval 10 min).

In vitro cytotoxicity assay

MDA-MB-231 cells were cultured in high-glucose DMEM supplemented with 10% fetal bovine serum (FBS) and seeded into 96-well plates at a density of 1 × 10^4 cells per well. After 24 h of incubation, the medium was replaced with 100 μL of fresh DMEM containing 1% DMSO and various concentrations (0-20 μM) of **TC1M** or **TC2M**. Following another 24 h of incubation, cell viability was assessed using the Cell Counting Kit-8 (CCK-8) assay. All experiments were performed in triplicate, and representative data are shown.

Cellular uptake and imaging

MDA-MB-231 and bEnd.3 cells were seeded into 24-well plates and cultured overnight at 37 °C in a 5% CO_2 incubator. To evaluate the cellular uptake of **TC1M** and **TC2M**, the cells were incubated with **TC1M (**1 μM**)** or **TC2M (**1 μM**)** for 4 h. After washing three times with PBS, the cells were imaged using an Olympus FV3000 confocal laser scanning microscope. The fluorescence signals of the probes were collected under the

following conditions: excitation wavelength of 561 nm and emission wavelength range of 650–750 nm.

In vitro dark cytotoxicity and phototoxicity

MDA-MB-231 cells were seeded into 96-well plates at a density of 1.2 × 10^4 cells per well and cultured for 24 h. The medium was then replaced with 100 µL of fresh DMEM containing various concentrations (0-20 µM) of **TC1M** or **TC2M**. After 4 h of incubation, the cells were washed three times with PBS, replenished with fresh medium, and exposed to white LED light irradiation (80 mW cm^{-2}) for 40 min. Cell viability was subsequently assessed using the CCK-8 assay. All experiments were performed in triplicate, and representative data are shown. For dark cytotoxicity evaluation, the same procedure was followed without light irradiation.

Intracellular reactive oxygen species (ROS) detection

Intracellular ROS generation was detected using DCFH-DA as a fluorescent probe. MDA-MB-231 cells were seeded into 24-well plates and cultured for 24 h. Then, the cells were incubated with **TC2M** (2 µM) for 4 h, followed by replacement with fresh medium containing DCFH-DA (5 µM) and further incubation for 30 min. After exposure to white LED light irradiation (80 mW cm^{-2}, 40 min), the cells were washed three times with PBS and imaged using an inverted fluorescence microscope.

Calcein-AM/PI co-staining assay

MDA-MB-231 cells were seeded into 24-well plates and cultured for 24 h. The cells were then treated with **TC2M** (2 µM) for 4 h, followed by exposure to white LED light irradiation (80 mW cm^{-2}) for 40 min. Subsequently, the cells were stained with a solution containing Calcein-AM (2 µM) and propidium iodide (PI, 1 µg/mL) for 30 min. After washing with PBS, fluorescence images were acquired using a confocal laser scanning microscope. Calcein-AM fluorescence was excited at 488 nm and collected in the range of 510-560 nm, while PI fluorescence was excited at 561 nm and collected in the range of 620-670 nm.

3. Synthesis and characterization of probes

General synthesis procedures

Procedure A: To a solution of bromothiophene derivatives (1 eq.) and carbazolyl boronic acid (1.3 eq.) in toluene (20-25 mL) was added $Pd(PPh_3)_4$ (0.02 eq.), cesium carbonate (3 eq.) and deionized water (5 mL). The resulting mixture was bubbled under Ar during 10 min, and then heated at 85°C during 6 h under Ar. After cooling to room temperature (RT), the reaction was filtered through diatomite pad, diluted with EtOAc (50 mL), washed with brine (40 mL×3). The combined organic layer was dried (Na_2SO_4), concentrated and purified with silica gel column chromatography to afford the resulting compounds.

Procedure B: To a solution of aldehyde derivatives (1 eq.) and tricyanofuran (TCF, 1.2 eq.) in CH_3CN (15-20 mL) was added piperidine (0.5 mL) and AcOH (0.5 mL). The mixture was refluxed until TLCmonitored the disappearance of starting materials. The reaction was diluted with EtOAc (50 mL), washed with HCl aqueous solution (2M, 40 mL) and brine (40 mL×3). The combined organic layer was dried (Na_2SO_4), concentrated and purified with silica gel column chromatography to afford the resulting products.

Procedure C: To a solution of azidocarbazoyl thiophene derivatives (1 eq.) and acetyl protected alkyl-PEG-mannose (1.5 eq.) in CH_2Cl_2 (20 mL) was added tetrakis(acetonitrile)copper(I) hexa-fluorophosphate ($Cu[(CH_3CN)_4]PF_6$, 0.5. eq). The resulting mixture was stirred under Ar at room temperature over 18 h. After TLCmonitoring the reaction, the reaction was diluted with CH_2Cl_2 (50 mL), washed with saturated EDTAsolution (40 mL×2) and brine (40 mL×2). The combined organic layer was dried (Na_2SO_4), concentrated and purified with silica gel column chromatography to afford the resulting products.

Procedure D: To the solution of acetyl mannose (1 eq.) decorated PSs in MeOH (20

mL) with traced THF were added MeONa (8 eq.) dissolved in DI water (2-3 mL). The resulting solution was stirred at room temperature under TLC monitoring the disappearance of all starting materials. The reaction was poured into HCl (2M, 30 mL), and extracted with CH_2Cl_2 (50 mL×4). The CH_2Cl_2 layers were combined, dried (Na_2SO_4), concentrated and purified with silica gel column chromatography to afford the final products.

Compounds synthesis

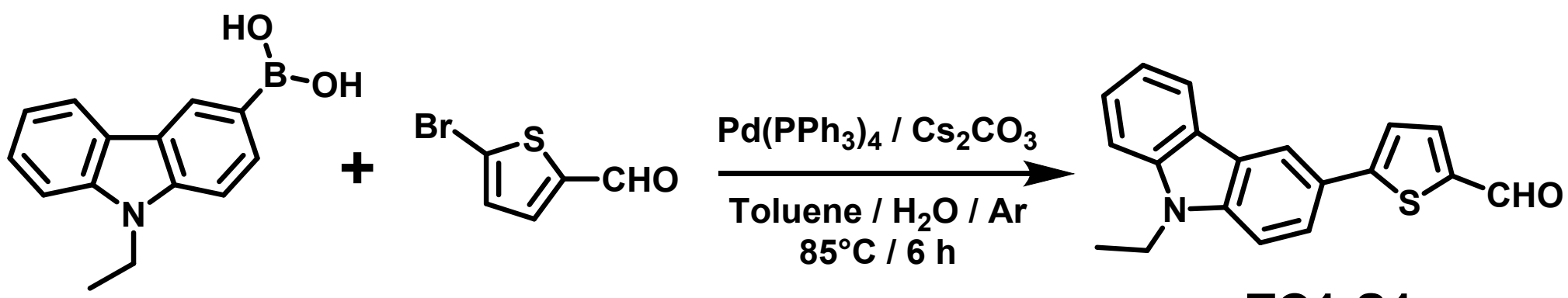

TC1-S1

*Synthesis of **TC1-S1***. Prepared according to the general procedure **A** from 5-bromothiophene-2-carbaldehyde (800 mg, 4.19 mmol, 1 eq.), *N*-ethyl-carbazolyl boronic acid (1.3 g, 5.44 mmol, 1.3 eq.), $Pd(PPh_3)_4$ (97 mg, 0.084 mmol, 0.02 eq.) and Cs_2CO_3 (4.09 g, 12.56 mmol, 3 eq.) affording compound **TC1-S1** (923 mg, 72%) as an amorphous yellow solid after chromatography purification (PE:EtOAc = 4:1, v/v).

^{1}H NMR (600 MHz, $CDCl_3$): δ (ppm) 9.88 (s, 1H), 8.39 (s, 1H), 8.14 (d, *J* = 7.7 Hz, 1H), 7.77 (d, *J* = 8.6 Hz, 1H), 7.75 (d, *J* = 3.9 Hz, 1H), 7.53 – 7.50 (m, 1H), 7.44 (s, 1H), 7.44 – 7.40 (m, 2H), 7.29 (t, *J* = 7.4 Hz, 1H), 4.37 (q, *J* = 7.3 Hz, 2H), 1.45 (s, 3H).

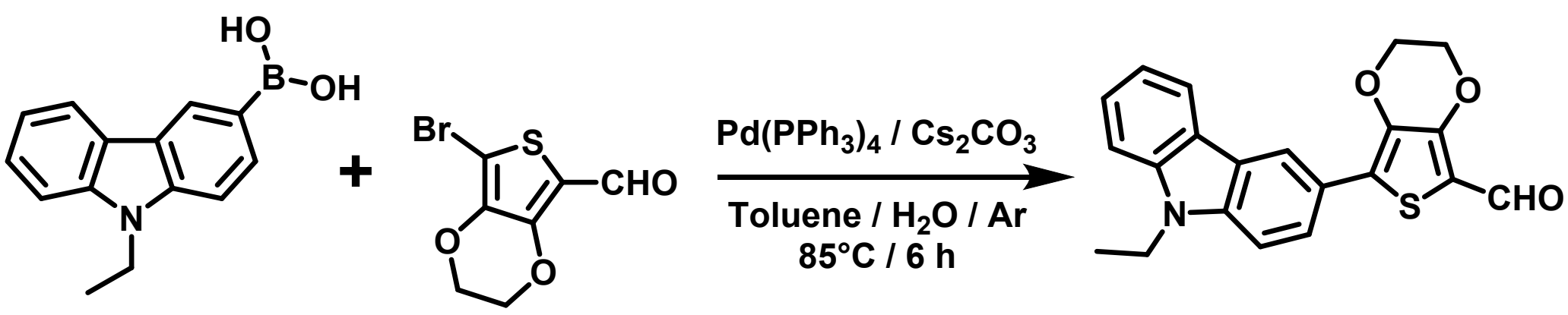

TC2-S1

*Synthesis of **TC2-S1***. Prepared according to the general procedure **A** from 7-bromo-2,3-dihydrothieno[3,4-*b*][1,4]dioxine-5-carbaldehyde (600 mg, 2.41 mmol, 1 eq.), *N*-ethyl-

carbazolyl boronic acid (749 mg, 3.13 mmol, 1.3 eq.), $Pd(PPh_3)_4$ (56 mg, 0.048 mmol, 0.02 eq.) and Cs_2CO_3 (2.35 g, 7.23 mmol, 3 eq.) affording compound **TC2-S1** (614 mg, 70%) as an amorphous yellow solid after chromatography purification (PE:EtOAc = 3:1, v/v).

^{1}H NMR (600 MHz, $CDCl_3$): δ (ppm) 9.95 (d, J = 10.8 Hz, 1H), 8.56 (d, J = 8.5 Hz, 1H), 8.15 (d, J = 8.4 Hz, 1H), 7.90 (d, J = 8.9 Hz, 1H), 7.51 (t, J = 8.2 Hz, 1H), 7.43 (dd, J = 11.3, 5.2 Hz, 2H), 7.28 (t, J = 7.1 Hz, 1H), 4.46 (s, 4H), 4.39 (t, J = 7.6 Hz, 2H), 1.45 (t, J = 7.5 Hz, 3H).

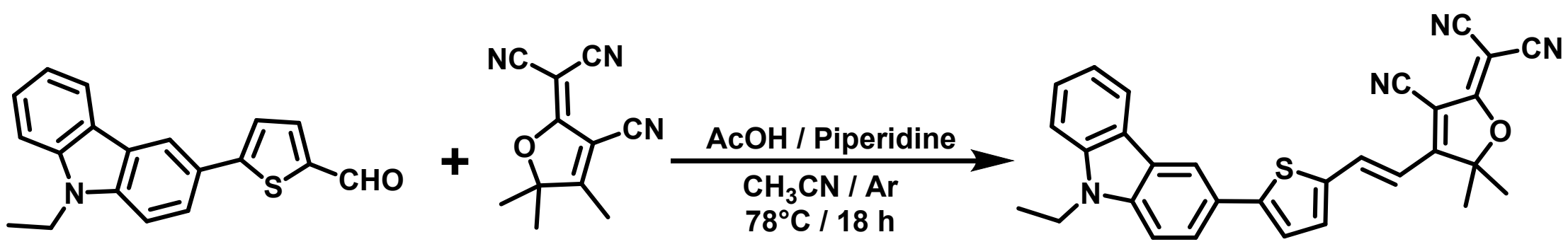

TC1-S1 TC1

*Synthesis of **TC1***. Prepared according to the general procedure **B** from **TC1-S1** (400 mg, 1.31 mmol, 1 eq.) and 2-(3-cyano-4,5,5-trimethylfuran-2(5H)-ylidene)malononitrile (TCF, 313 mg, 1.57 mmol, 1.2 eq.) affording compound **TC1** (325 mg, 51%) as an amorphous dark blue solid after chromatography purification (PE:EtOAc = 2:1, v/v).

^{1}H NMR (600 MHz, $CDCl_3$): δ (ppm) 8.41 (s, 1H), 8.16 (d, J = 7.8 Hz, 1H), 7.84 (d, J = 15.7 Hz, 1H), 7.79 (d, J = 8.5 Hz, 1H), 7.54 (t, J = 7.8 Hz, 1H), 7.50 (d, J = 4.0 Hz, 1H), 7.48 – 7.42 (m, 3H), 7.31 (t, J = 7.4 Hz, 1H), 6.68 (d, J = 15.7 Hz, 1H), 4.41 (q, J = 7.3 Hz, 2H), 1.78 (s, 6H), 1.48 (t, J = 7.3 Hz, 3H). ^{13}C NMR (150 MHz, DMSO-d_6): δ (ppm) 177.3, 175.2, 154.7, 141.2, 140.75, 140.67, 139.1, 138.5, 130.1, 127.0, 125.4, 124.7, 124.0, 123.4, 122.6, 121.4, 120.0, 119.1, 113.4, 112.7, 112.5, 111.6, 110.5, 110.1, 99.2, 97.4, 38.1, 27.0, 26.0, 14.2. HR-ESI-MS m/z: calcd. for $C_{30}H_{23}N_4OS^+$ $[M+H]^+$ 487.1587 found 487.1579.

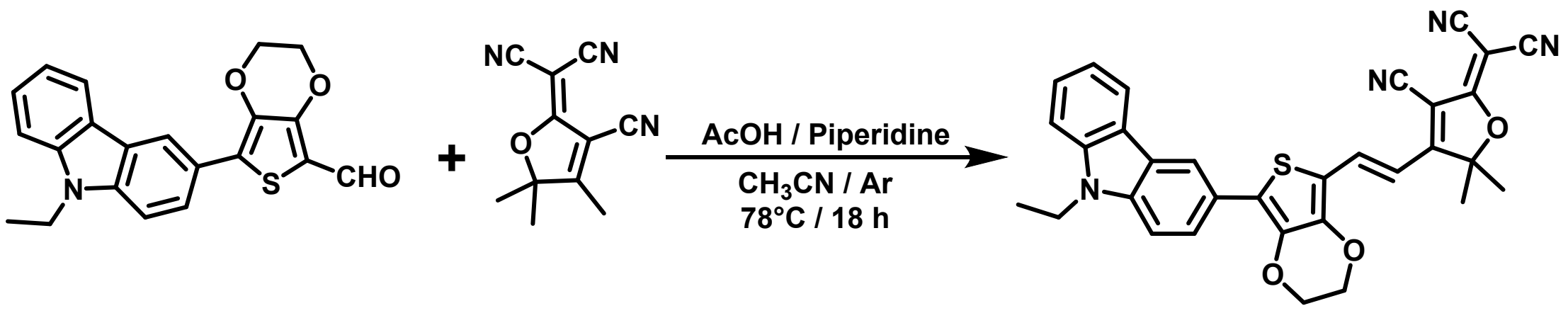

*Synthesis of **TC2***. Prepared according to the general procedure **B** from **TC2-S1** (300 mg, 0.83 mmol, 1 eq.) and 2-(3-cyano-4,5,5-trimethylfuran-2(5H)-ylidene)malononitrile (TCF, 197 mg, 0.99 mmol, 1.2 eq.) affording compound **TC2** (214 mg, 48%) as an amorphous dark blue solid after chromatography purification (PE:EtOAc = 1:1, v/v).

^{1}H NMR (600 MHz, $CDCl_3$): δ (ppm) 8.60 (s, 1H), 8.20 (d, *J* = 7.6 Hz, 1H), 7.95 (d, *J* = 8.7 Hz, 1H), 7.83 – 7.76 (m, 1H), 7.55 (s, 1H), 7.47 (d, *J* = 9.0 Hz, 2H), 7.11 (s, 1H), 7.00 (s, 1H), 5.37 (s, 2H), 4.51 (d, *J* = 21.5 Hz, 2H), 4.43 (d, *J* = 7.4 Hz, 2H), 1.80 (s, 3H), 1.27 (s, 6H). Due to the poor solubility in common deuterium reagents, we cannot provide the ^{13}C NMR spectral of **TC2**. HR-ESI-MS *m/z*: calcd. for $C_{32}H_{25}N_4O_3S^+$ $[M+H]^+$ 545.1641 found 545.1635.

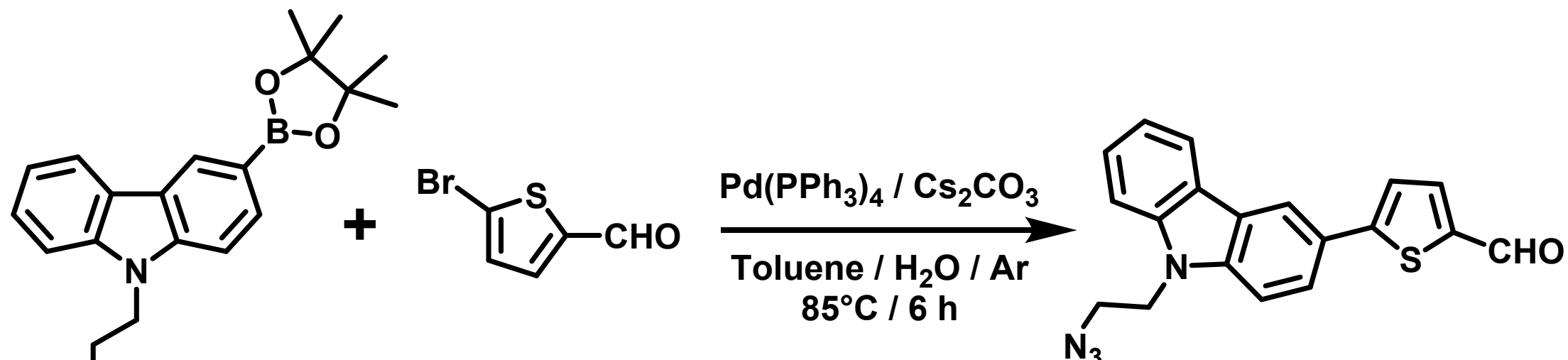

*Synthesis of **TC1M-S1***. Prepared according to the general procedure **A** from 5-bromothiophene-2-carbaldehyde (500 mg, 2.62 mmol, 1 eq.), 9-(2-azidoethyl)-3-(4,4,5,5-tetramethyl-1,3,2-dioxaborolan-2-yl)-9H-carbazole (1.23 g, 3.40 mmol, 1.3 eq.), $Pd(PPh_3)_4$ (60 mg, 0.052 mmol, 0.02 eq.) and Cs_2CO_3 (2.56 g, 7.85 mmol, 3 eq.) affording compound **TC1M-S1** (633 mg, 70%) as an amorphous yellow solid after chromatography purification (PE:EtOAc = 4:1, v/v).

^{1}H NMR (600 MHz, DMSO-d_6): δ (ppm) ^{1}H NMR (600 MHz, DMSO-d_6) δ 9.90 (s,

1H), 8.67 (s, 1H), 8.29 (d, *J* = 8.8 Hz, 1H), 8.06 (d, *J* = 3.9 Hz, 1H), 7.90 (d, *J* = 8.6 Hz, 1H), 7.79 (d, *J* = 3.9 Hz, 1H), 7.77 (d, *J* = 8.6 Hz, 1H), 7.70 (d, *J* = 8.2 Hz, 1H), 7.53 – 7.48 (m, 1H), 7.28 (t, *J* = 7.4 Hz, 1H), 4.83 (t, *J* = 5.9 Hz, 2H), 4.08 (t, *J* = 5.9 Hz, 2H).

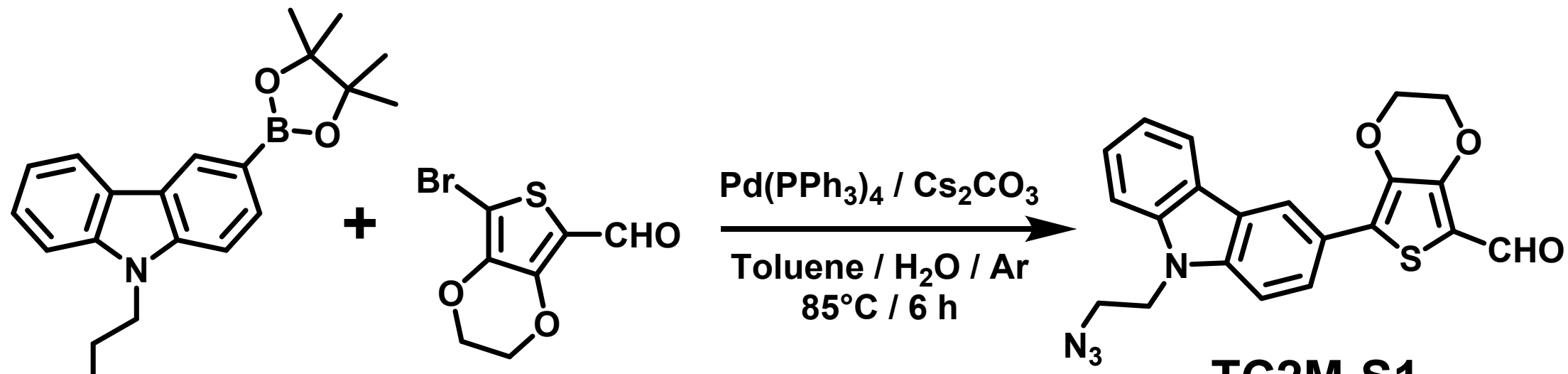

TC2M-S1

Synthesis of ***TC2M-S1***. Prepared according to the general procedure **A** from 7-bromo-2,3-dihydrothieno[3,4-*b*][1,4]dioxine-5-carbaldehyde (500 mg, 2.01 mmol, 1 eq.), 9-(2-azidoethyl)-3-(4,4,5,5-tetramethyl-1,3,2-dioxaborolan-2-yl)-9H-carbazole (945 mg, 2.61 mmol, 1.3 eq.), $Pd(PPh_3)_4$ (46 mg, 0.040 mmol, 0.02 eq.) and Cs_2CO_3 (1.96 g, 6.02 mmol, 3 eq.) affording compound **TC2M-S1** (512 mg, 63%) as an amorphous yellow solid after chromatography purification (PE:EtOAc = 3:1, v/v).

^{1}H NMR (600 MHz, $CDCl_3$): δ (ppm) 9.85 (s, 1H), 8.48 (s, 1H), 8.14 – 8.05 (m, 1H), 7.86 (d, *J* = 7.2 Hz, 1H), 7.47 (d, *J* = 7.7 Hz, 1H), 7.43 – 7.38 (m, 2H), 7.27 (d, *J* = 8.0 Hz, 1H), 4.59 (d, *J* = 4.4 Hz, 2H), 4.47 – 4.35 (m, 2H), 3.84 (d, *J* = 4.6 Hz, 2H), 3.35 (s, 2H).

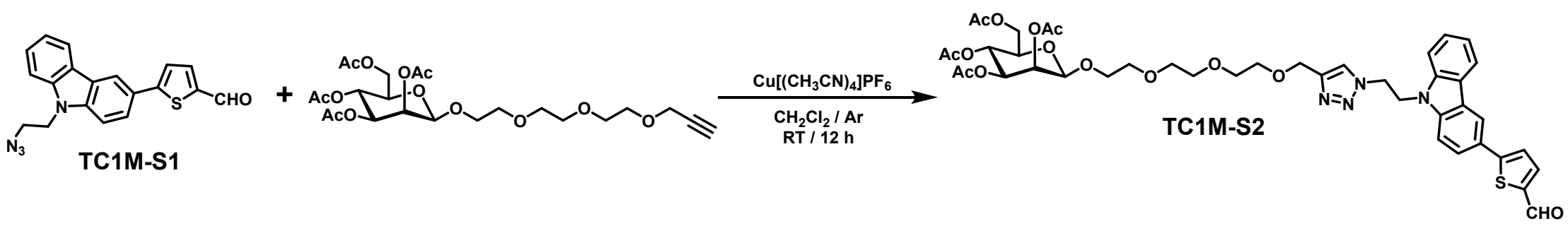

Synthesis of ***TC1M-S2***. Prepared according to the general procedure **C** from **TC1M-S1** (300 mg, 0.87 mmol, 1 eq.), acetyl protected alkyl-PEG-mannose (674 mg, 1.30 mmol, 1.3 eq.) and $Cu[(CH_3CN)_4]PF_6$ (161 mg, 0.43 mmol, 0.5 eq.) affording compound **TC1M-S2** (452 mg, 60%) as an amorphous yellow solid after chromatography purification (CH_2Cl_2:MeOH = 60:1, v/v).

^{1}H NMR (600 MHz, $CDCl_3$): δ (ppm) 9.96 (s, 1H), 8.42 (s, 1H), 8.18 (d, *J* = 7.7 Hz, 1H), 7.85 (d, *J* = 3.8 Hz, 1H), 7.77 (d, *J* = 8.3 Hz, 1H), 7.56 – 7.50 (m, 2H), 7.36 (t, *J* = 7.4 Hz, 1H), 7.29 (d, *J* = 8.2 Hz, 1H), 7.21 (d, *J* = 8.4 Hz, 1H), 6.99 (s, 1H), 5.26 (t, *J* = 9.5 Hz, 1H), 5.14 (t, *J* = 9.7 Hz, 1H), 5.05 (t, *J* = 8.8 Hz, 1H), 4.91 (s, 4H), 4.64 (d, *J* = 8.0 Hz, 1H), 4.31 (dd, *J* = 12.4, 4.6 Hz, 2H), 4.18 (dd, *J* = 12.5, 2.3 Hz, 1H), 3.97 (dd, *J* = 10.5, 5.1 Hz, 1H), 3.76 (dd, *J* = 11.2, 4.1 Hz, 2H), 3.63 (d, *J* = 4.5 Hz, 2H), 3.55 (d, *J* = 5.0 Hz, 2H), 3.50 – 3.42 (m, 4H), 3.31 (d, *J* = 4.6 Hz, 2H), 2.13 (s, 3H), 2.08 (s, 6H), 2.07 (s, 3H).

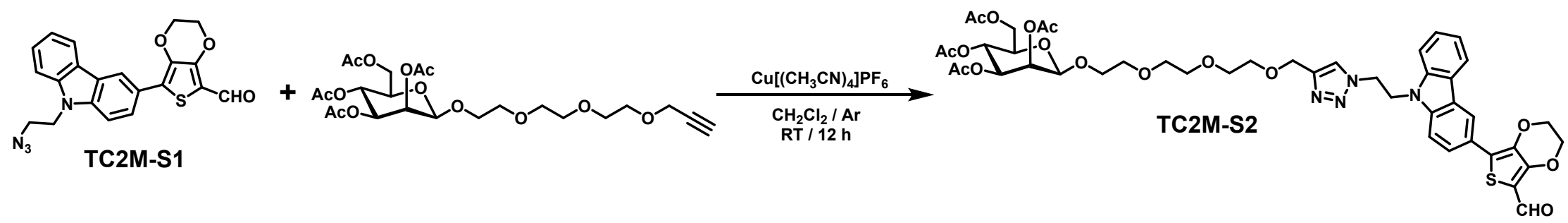

Synthesis of ***TC2M-S2***. Prepared according to the general procedure **C** from **TC2M-S1** (300 mg, 0.74 mmol, 1 eq.), acetyl protected alkyl-PEG-mannose (500 mg, 0.96 mmol, 1.3 eq.) and $Cu[(CH_3CN)_4]PF_6$ (138 mg, 0.37 mmol, 0.5 eq.) affording compound **TC2M-S2** (421 mg, 61%) as an amorphous yellow solid after chromatography purification (CH_2Cl_2:MeOH = 50:1, v/v).

^{1}H NMR (600 MHz, $CDCl_3$): δ (ppm) 9.94 (s, 1H), 8.47 (s, 1H), 8.11 (d, *J* = 7.7 Hz, 1H), 7.82 (d, *J* = 8.5 Hz, 1H), 7.45 (t, *J* = 7.7 Hz, 1H), 7.28 (d, *J* = 14.8 Hz, 1H), 7.19 (d, *J* = 8.1 Hz, 1H), 7.12 (d, *J* = 8.5 Hz, 1H), 6.89 (s, 1H), 5.33 (td, *J* = 9.4, 8.8, 3.7 Hz, 2H), 5.28 – 5.25 (m, 1H), 4.85 (s, 1H), 4.82 (s, 4H), 4.47 (d, *J* = 6.8 Hz, 4H), 4.43 (s, 2H), 4.29 (dd, *J* = 12.1, 4.8 Hz, 1H), 4.10 – 4.01 (m, 2H), 3.78 (dt, *J* = 9.7, 4.5 Hz, 1H), 3.68 (d, *J* = 8.3 Hz, 1H), 3.60 (d, *J* = 4.3 Hz, 2H), 3.52 (d, *J* = 4.8 Hz, 2H), 3.48 (d, *J* = 4.6 Hz, 2H), 3.43 (d, *J* = 4.6 Hz, 2H), 3.24 (d, *J* = 4.7 Hz, 2H), 2.16 (s, 3H), 2.10 (s, 3H), 2.04 (s, 3H), 1.99 (s, 3H).

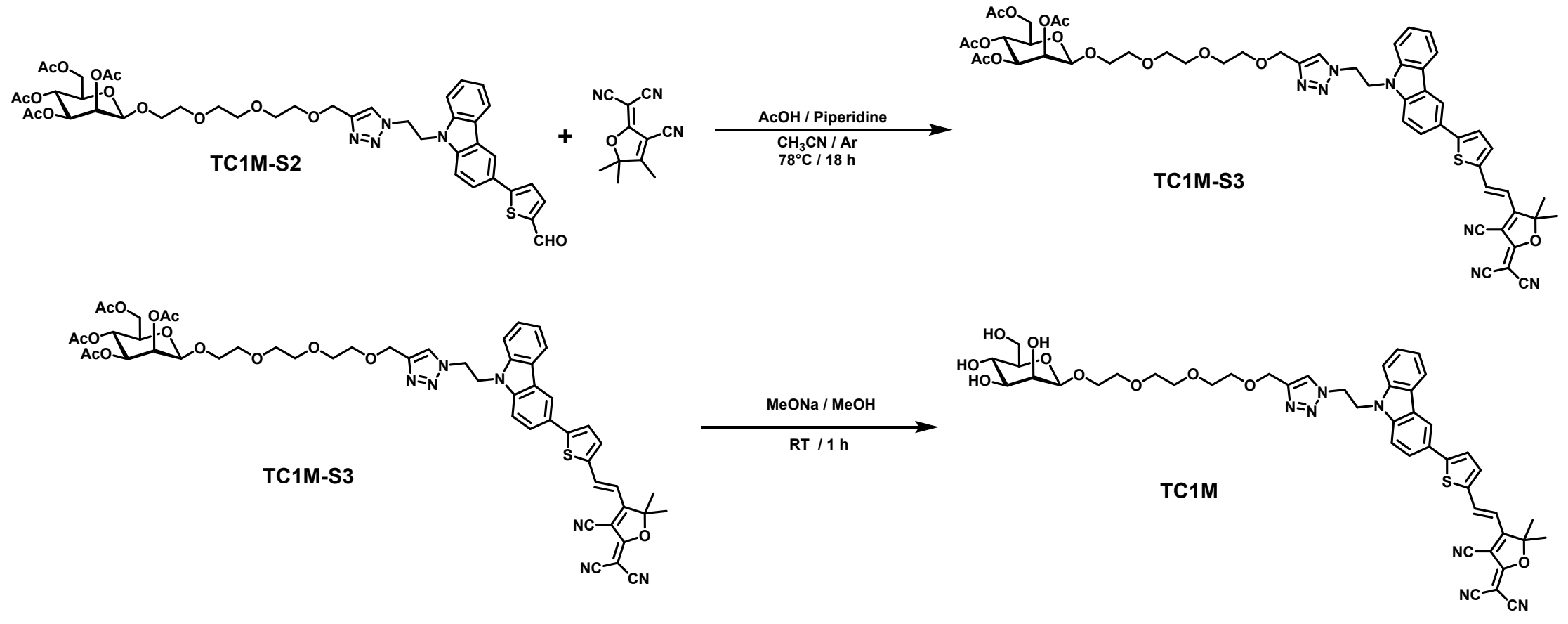

Synthesis of ***TC1M***. Prepared according to the general procedure **B** from **TC1M-S2** (300 mg, 0.35 mmol, 1 eq.) and TCF (83 mg, 0.42 mmol, 1.2 eq.) affording crude compound **TC1M-S3** (185 mg, 50%) without purification for next deprotection step. Prepared according to the general procedure **D** from **TC1M-S3** (150 mg, 0.14 mmol, 1 eq.) and MeONa (61 mg, 1.13 mmol, 8 eq.) affording compound **TC1M** (85 mg, 67%) as an amorphous dark blue solid after chromatography purification (CH_2Cl_2:MeOH = 10:1, v/v).

^{1}H NMR (600 MHz, DMSO-d_6): 8.65 (d, J = 1.9 Hz, 1H), 8.27 (d, J = 7.7 Hz, 1H), 8.18 (d, J = 15.9 Hz, 1H), 7.91 (s, 1H), 7.89 (d, J = 4.1 Hz, 1H), 7.85 (dd, J = 8.6, 1.9 Hz, 1H), 7.81 (d, J = 4.0 Hz, 1H), 7.44 (d, J = 8.5 Hz, 3H), 7.28 – 7.22 (m, 1H), 6.79 (d, J = 15.9 Hz, 1H), 4.92 (t, J = 5.7 Hz, 2H), 4.84 (t, J = 5.7 Hz, 2H), 4.73 – 4.68 (m, 2H), 4.62 (s, 1H), 4.55 (s, 1H), 4.43 (s, 1H), 4.37 (s, 2H), 3.69 – 3.62 (m, 2H), 3.59 (s, 1H), 3.52 (q, J = 4.1, 3.6 Hz, 2H), 3.48 – 3.47 (m, 3H), 3.45 (d, J = 4.9 Hz, 6H), 3.41 (t, J = 3.2 Hz, 4H), 3.27 (t, J = 4.8 Hz, 2H). ^{13}C NMR (150 MHz, DMSO-d_6): δ (ppm) 177.4, 175.3, 154.4, 144.4, 141.3, 141.1, 140.9, 139.5, 138.6, 127.0, 125.5, 125.0, 124.6, 124.3, 123.4, 122.6, 121.3, 120.3, 118.9, 113.4, 112.7, 112.5, 111.6, 110.3, 109.9, 103.4, 99.3, 97.5, 77.3, 77.2, 73.8, 70.5, 70.2, 70.1, 70.1, 68.8, 68.2, 63.6, 61.5, 55.4, 53.6, 48.8, 43.5, 26.0. HR-ESI-MS *m/z*: calcd. for $C_{45}H_{48}N_7O_{10}S^+$ $[M+H]^+$ 878.3178 found 878.3173.

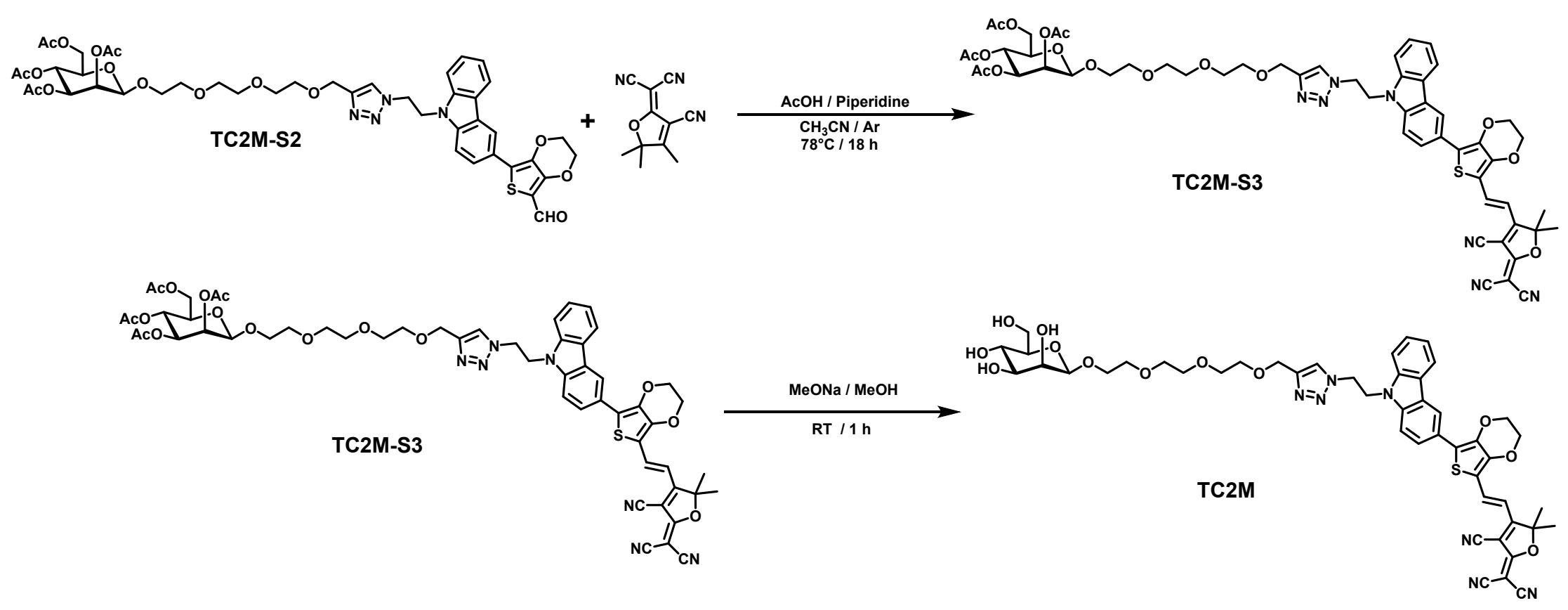

*Synthesis of **TC2M***. Prepared according to the general procedure **B** from **TC2M-S2** (360 mg, 0.38 mmol, 1 eq.) and TCF (91 mg, 0.46 mmol, 1.2 eq.) affording crude compound **TC2M-S3** (205 mg, 48%) without purification for next deprotection step. Prepared according to the general procedure **D** from **TC2M-S3** (150 mg, 0.13 mmol, 1 eq.) and MeONa (58 mg, 1.07 mmol, 8 eq.) affording compound **TC2M** (74 mg, 58%) as an amorphous dark blue solid after chromatography purification (CH_2Cl_2:MeOH = 10:1, v/v).

^{1}H NMR (600 MHz, DMSO-d_6): δ (ppm) 8.57 (s, 1H), 8.20 (d, *J* = 7.7 Hz, 1H), 8.03 (d, *J* = 15.5 Hz, 1H), 7.91 (s, 1H), 7.85 (d, *J* = 8.7 Hz, 1H), 7.48 – 7.41 (m, 3H), 7.25 (t, *J* = 7.2 Hz, 1H), 6.78 (d, *J* = 15.4 Hz, 1H), 4.92 (t, *J* = 5.8 Hz, 2H), 4.85 (d, *J* = 5.6 Hz, 2H), 4.73 (d, *J* = 5.3 Hz, 1H), 4.71 (d, *J* = 4.4 Hz, 1H), 4.63 (s, 1H), 4.60 (s, 2H), 4.56 (d, *J* = 6.0 Hz, 1H), 4.52 (s, 2H), 4.43 (t, *J* = 6.0 Hz, 1H), 4.37 (s, 2H), 3.69 – 3.63 (m, 2H), 3.59 (s, 1H), 3.53 – 3.51 (m, 2H), 3.50 – 3.47 (m, 3H), 3.45 (d, *J* = 5.6 Hz, 4H), 3.42 (t, *J* = 5.0 Hz, 2H), 3.28 (t, *J* = 4.9 Hz, 2H), 1.75 (s, 6H). ^{13}C NMR (150 MHz, DMSO-d_6): δ (ppm) 177.6, 175.1, 148.9, 144.4, 140.9, 140.6, 138.7, 129.9, 127.0, 125.1, 125.0, 123.2, 123.1, 122.5, 121.1, 120.4, 119.1, 113.8, 113.2, 113.0, 112.4, 110.1, 109.9, 100.4, 98.7, 93.8, 74.4, 71.4, 70.7, 70.2, 70.2, 70.1, 70.0, 68.8, 67.4, 66.3, 66.1, 65.2, 63.6, 61.7, 52.3, 48.7, 43.5, 25.0. HR-ESI-MS *m*/*z*: calcd. for $C_{47}H_{50}N_7O_{12}S^+$ $[M+H]^+$ 936.3233, found 936.3222.

4. Original spectra of new compounds

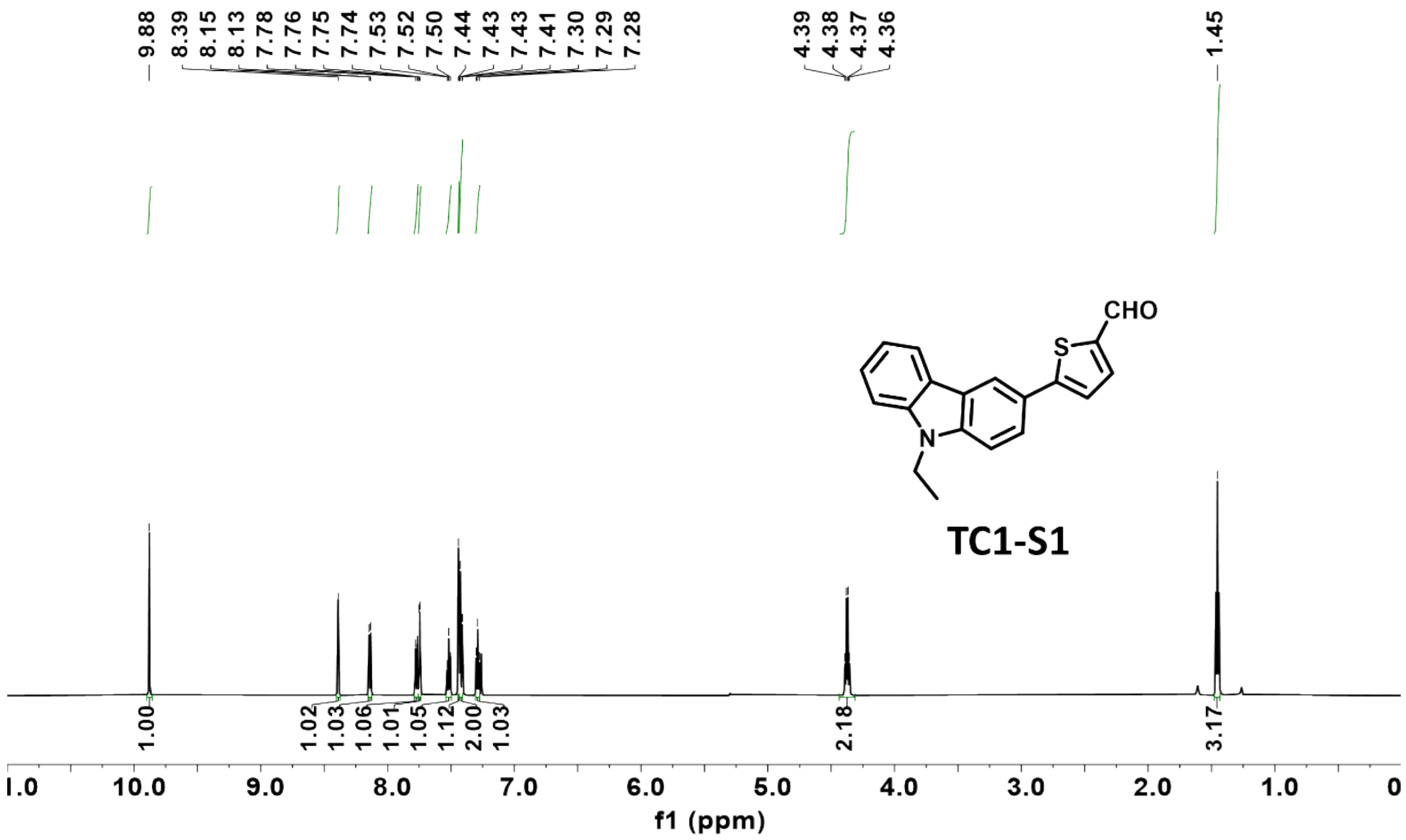

Figure S19. ^1H NMR of compound **TC1-S1**.

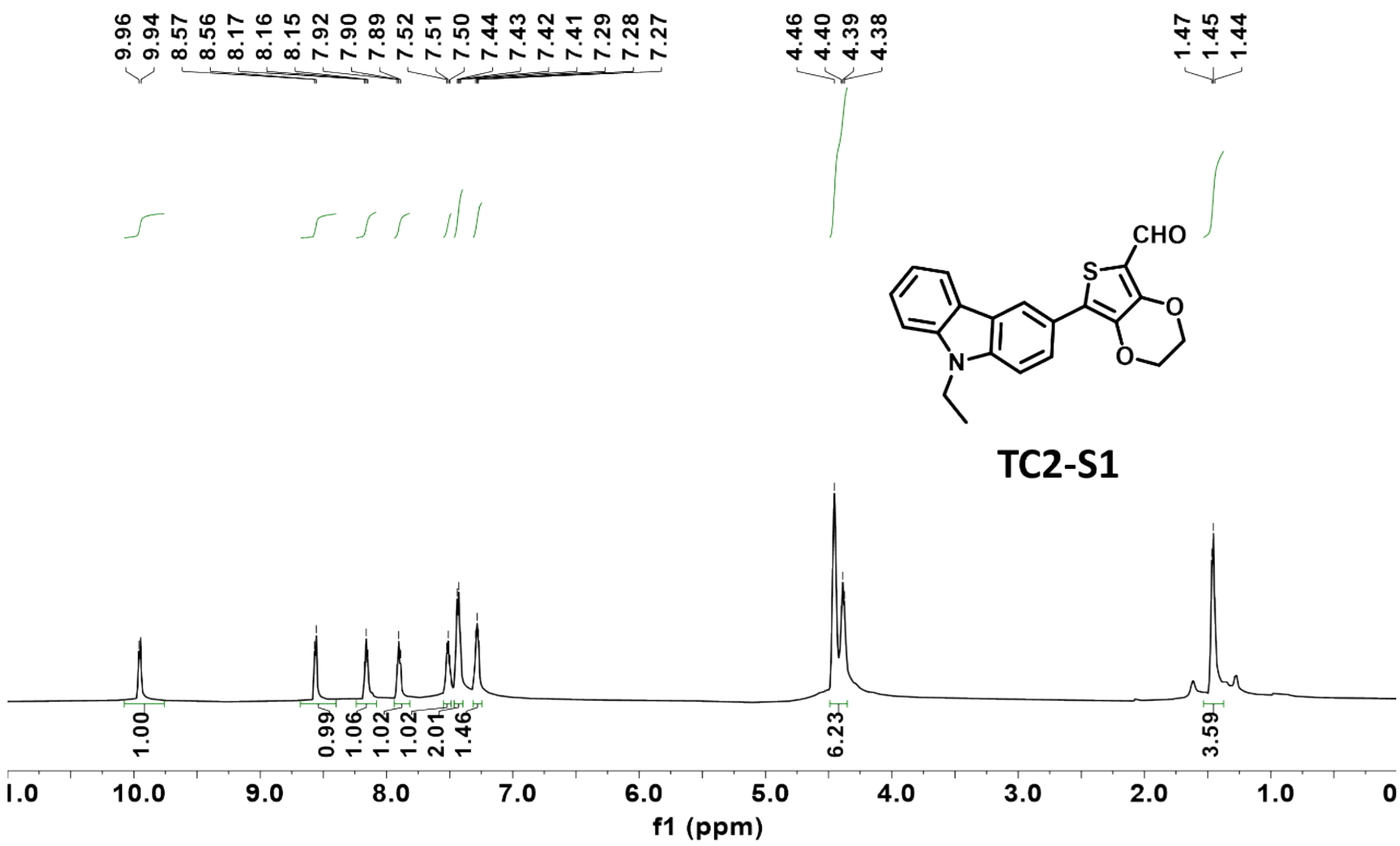

Figure S20. ^1H NMR of compound **TC2-S1**.

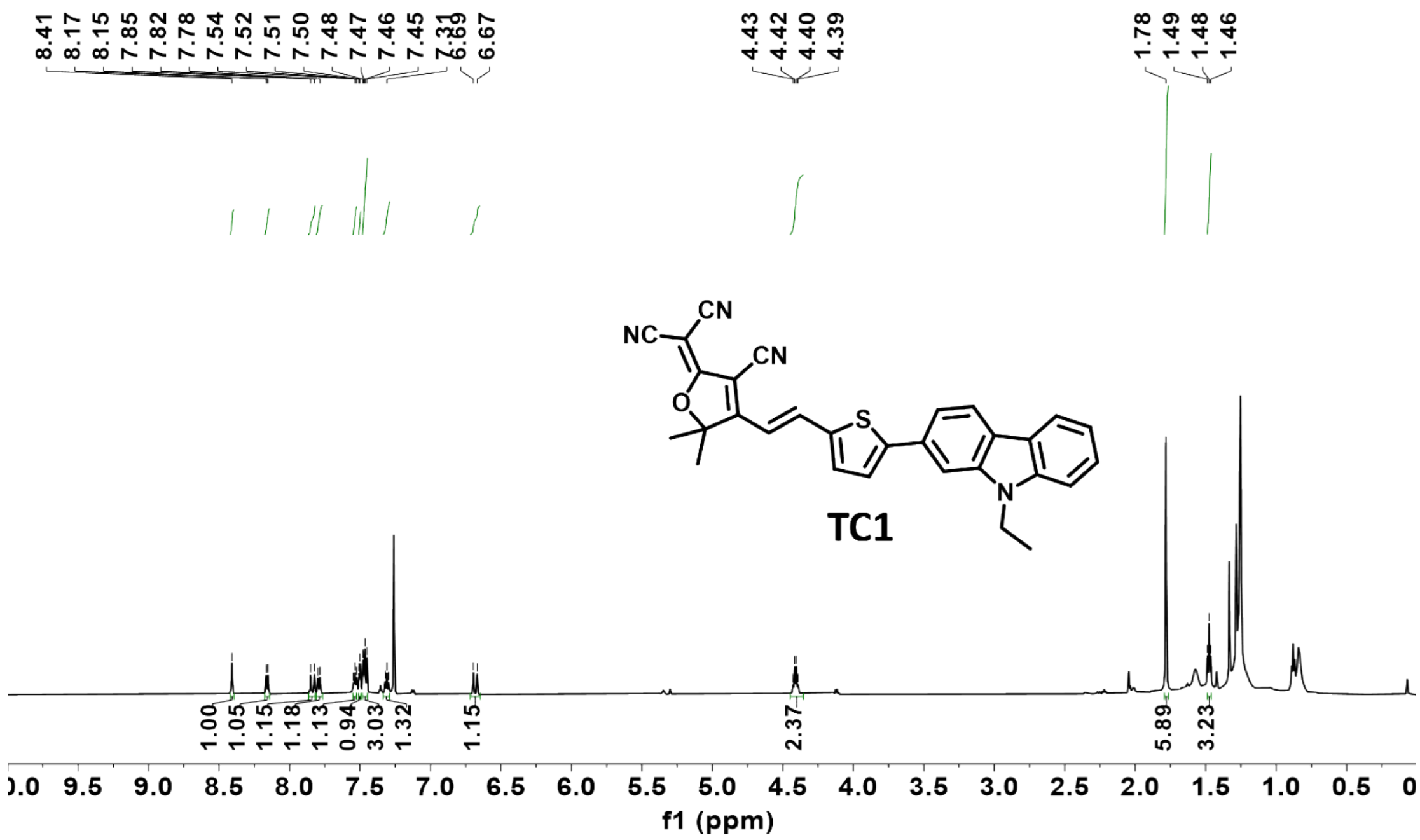

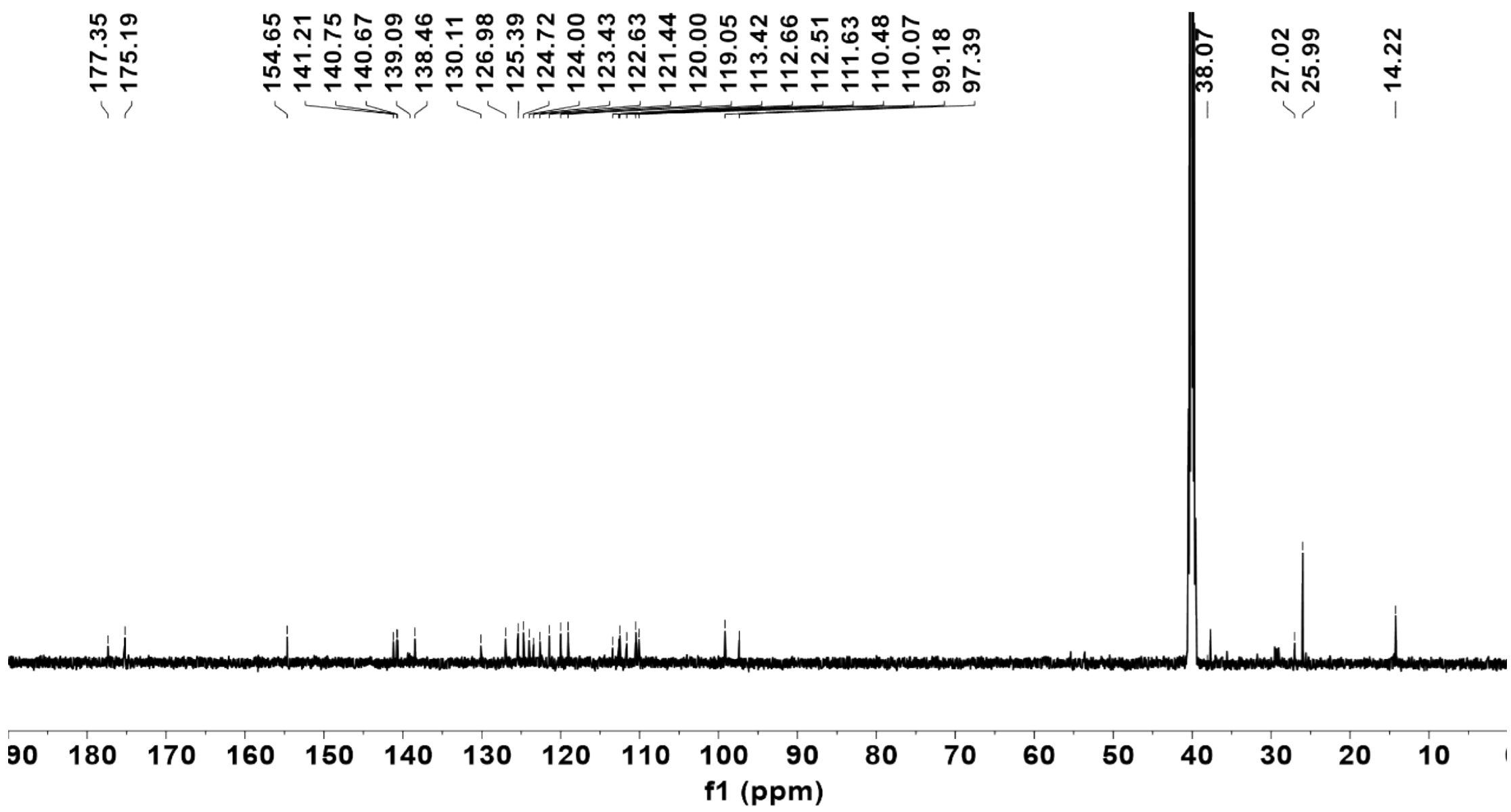

Figure S21. ^{1}H NMR and ^{13}C NMR of compound **TC1**.

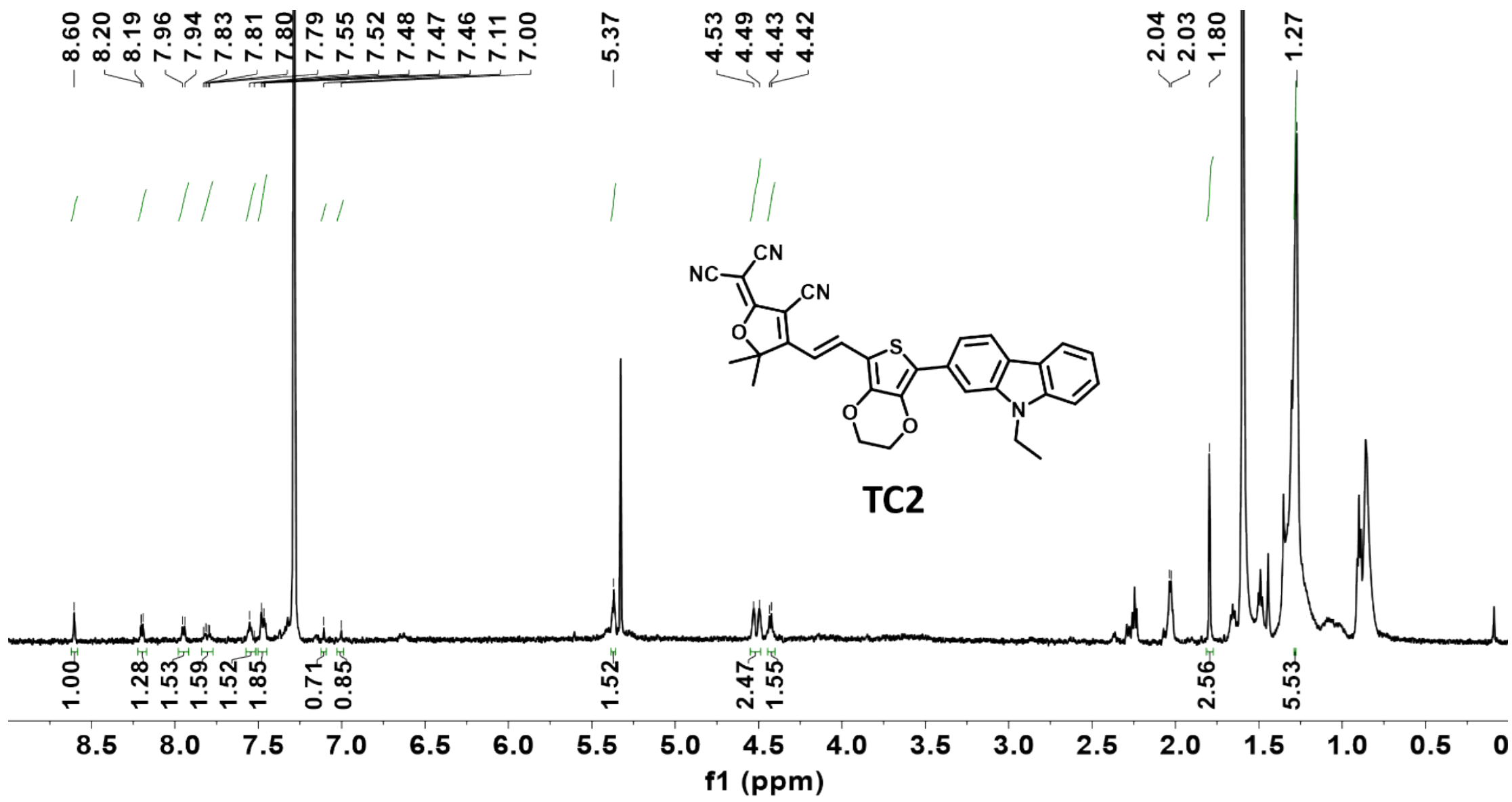

Figure S22. ^{1}H NMR of compound **TC2**.

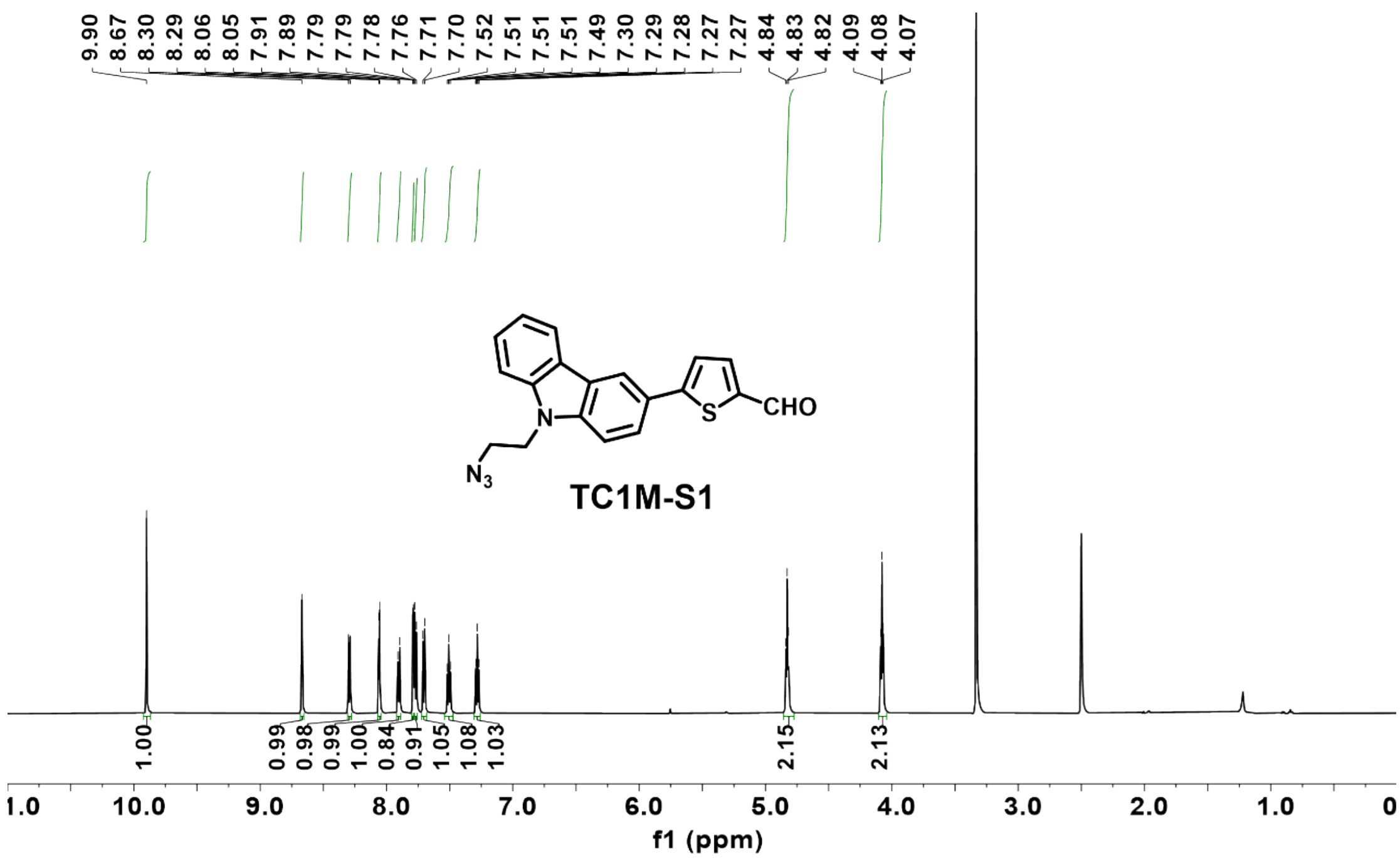

Figure S23. ^{1}H NMR of compound **TC1M-S1**.

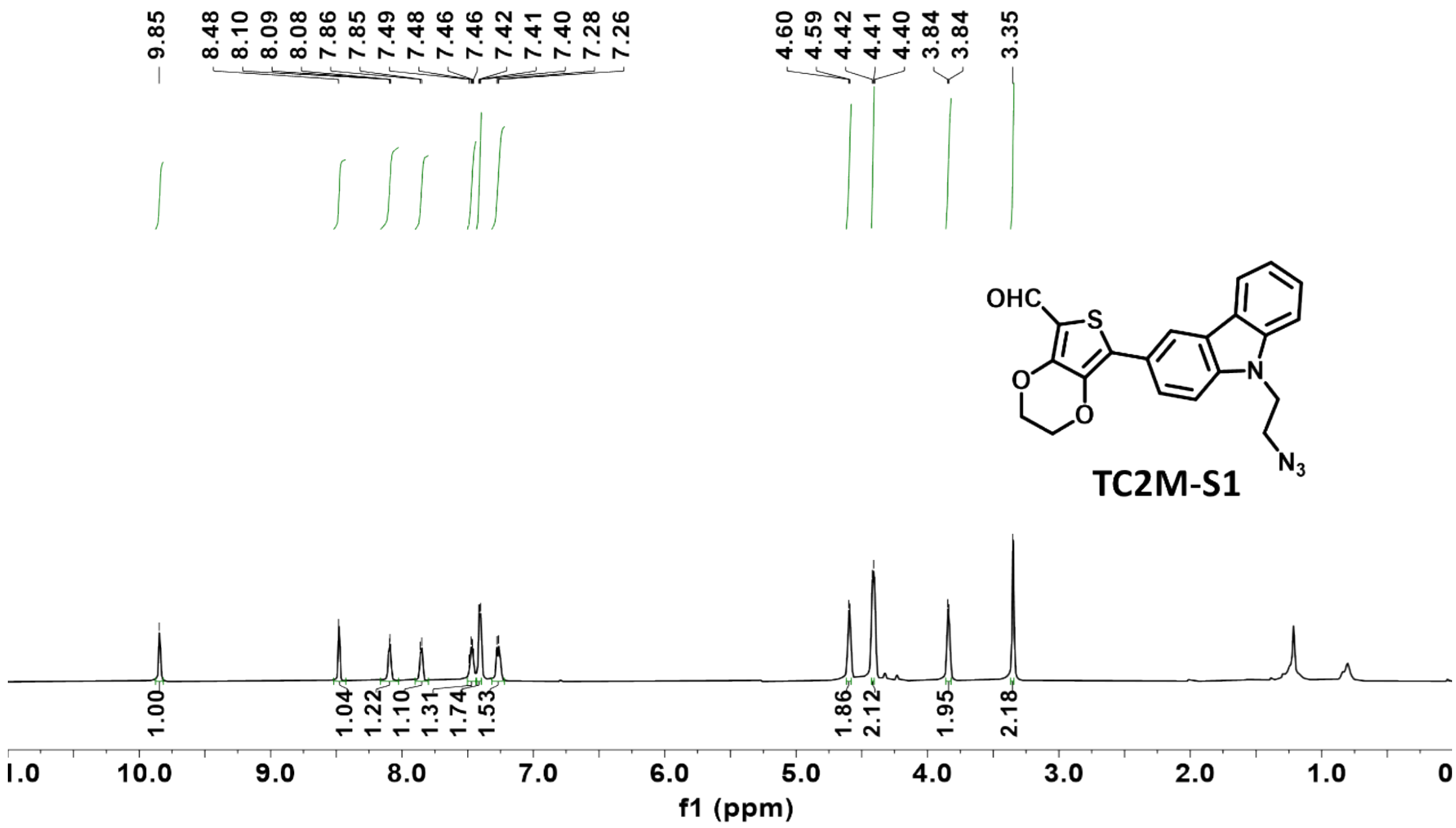

Figure S24. ^{1}H NMR of compound **TC2M-S1**.

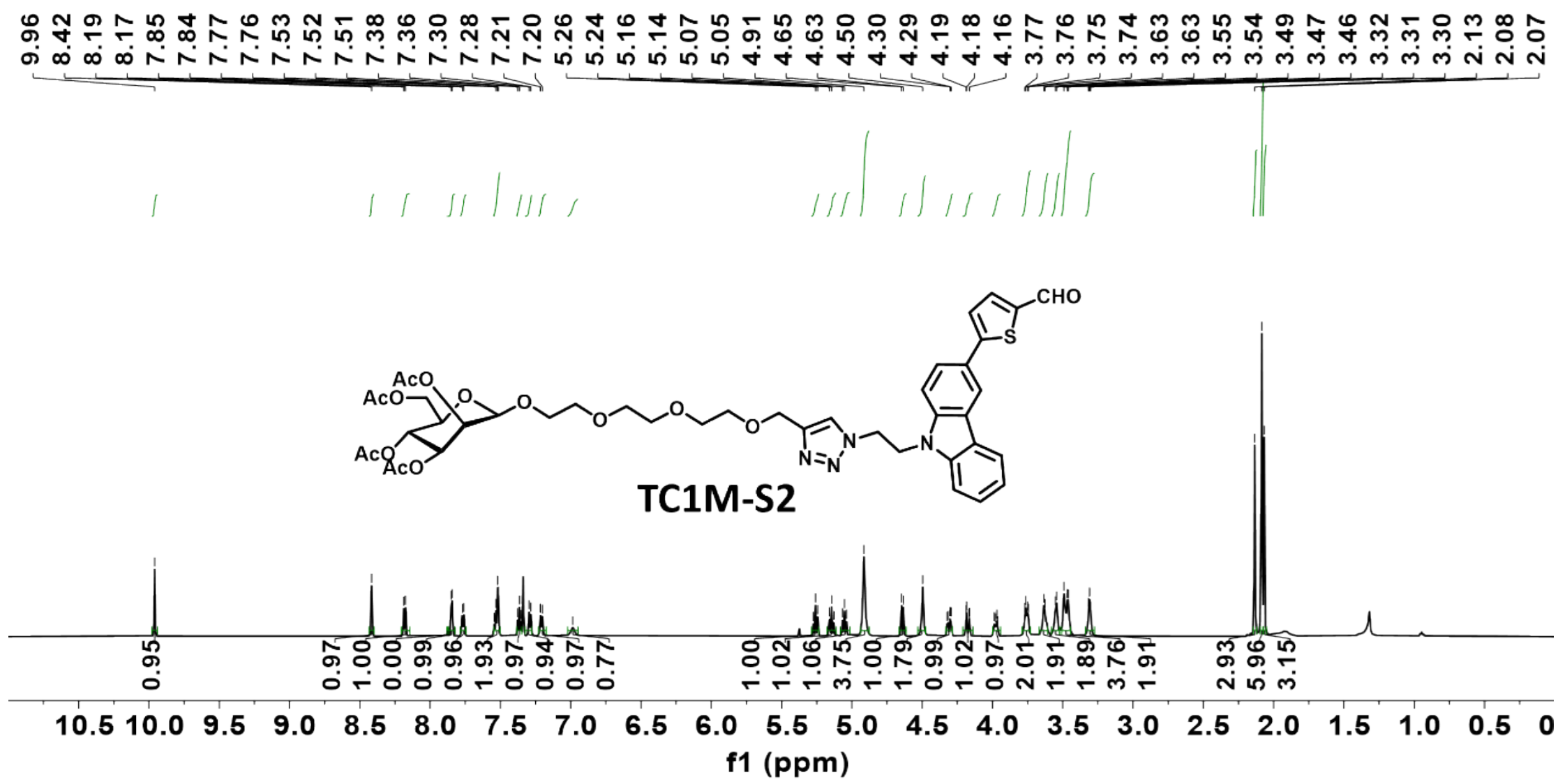

Figure S25. ^{1}H NMR of compound **TC1M-S2**.

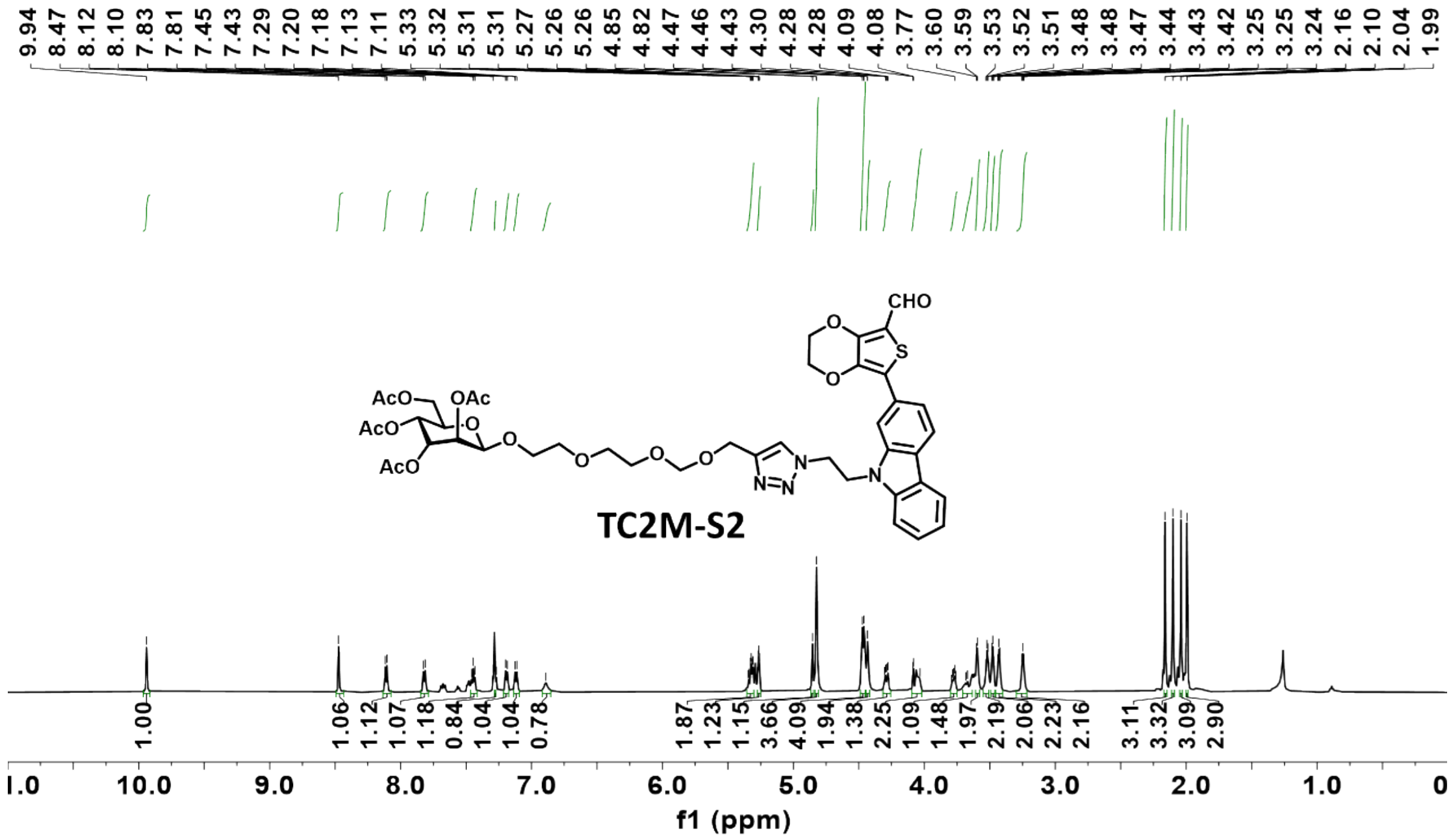

Figure S26. ^{1}H NMR of compound **TC2M-S2**.

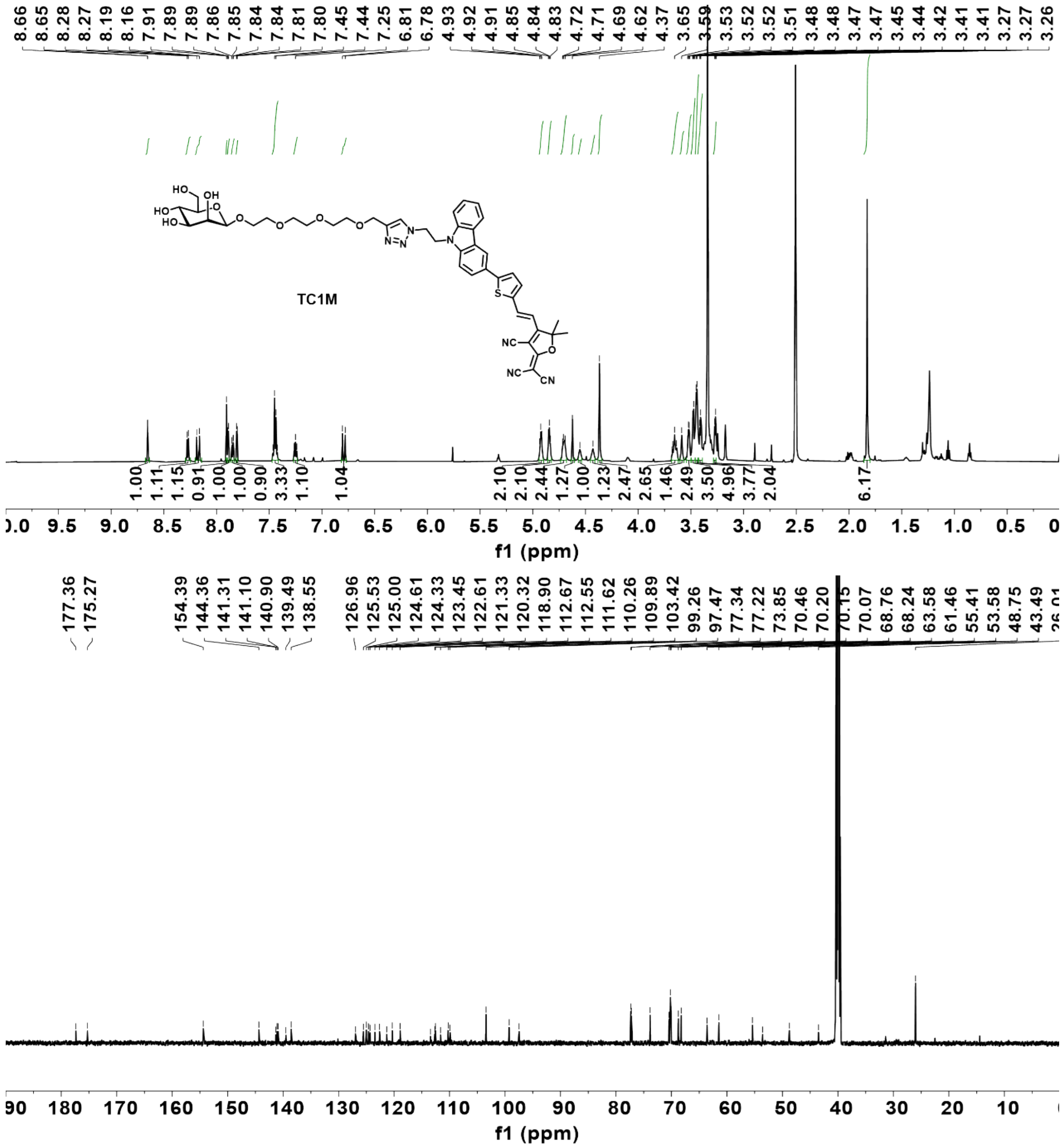

Figure S27. ^{1}H NMR and ^{13}C NMR of compound **TC1M**.

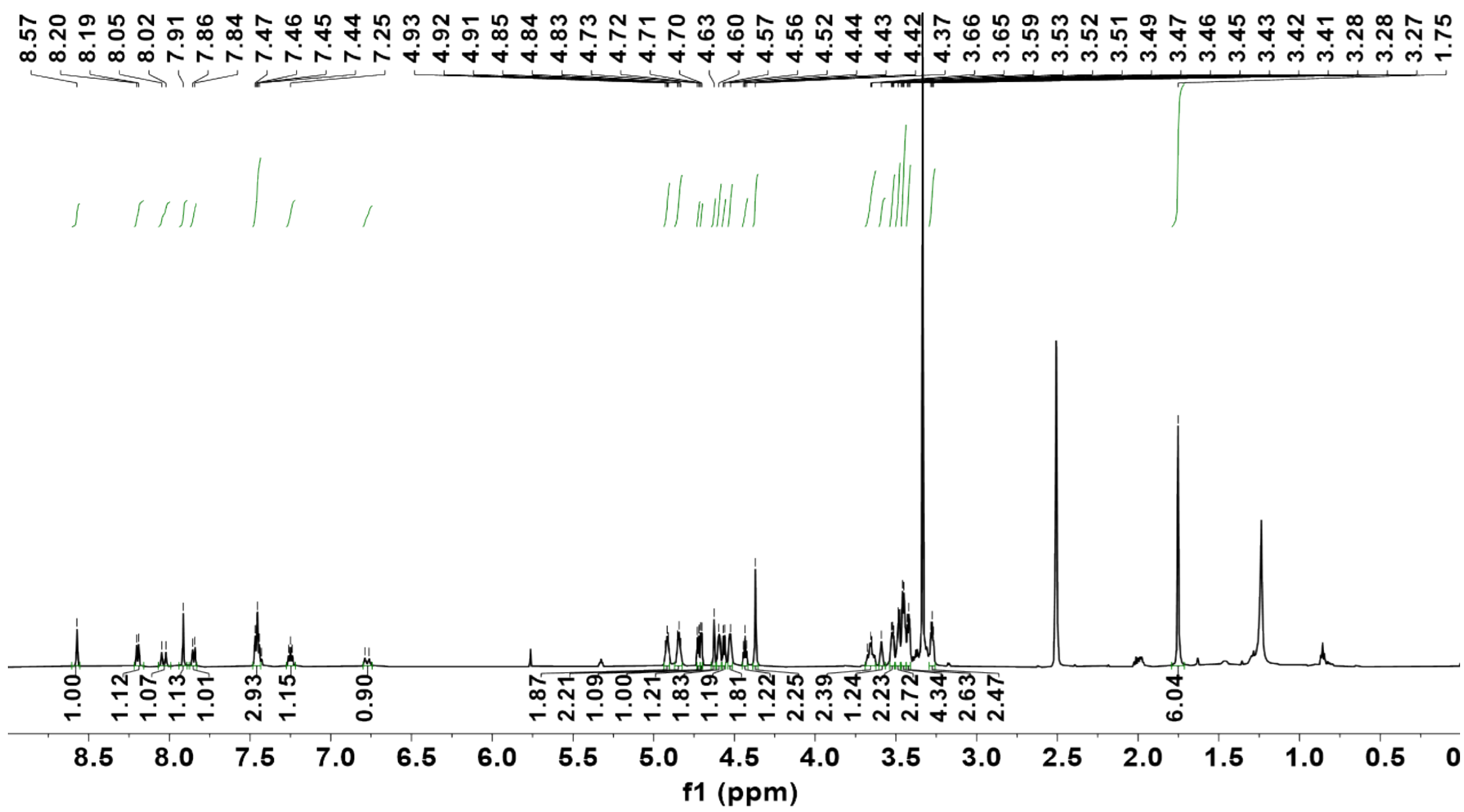

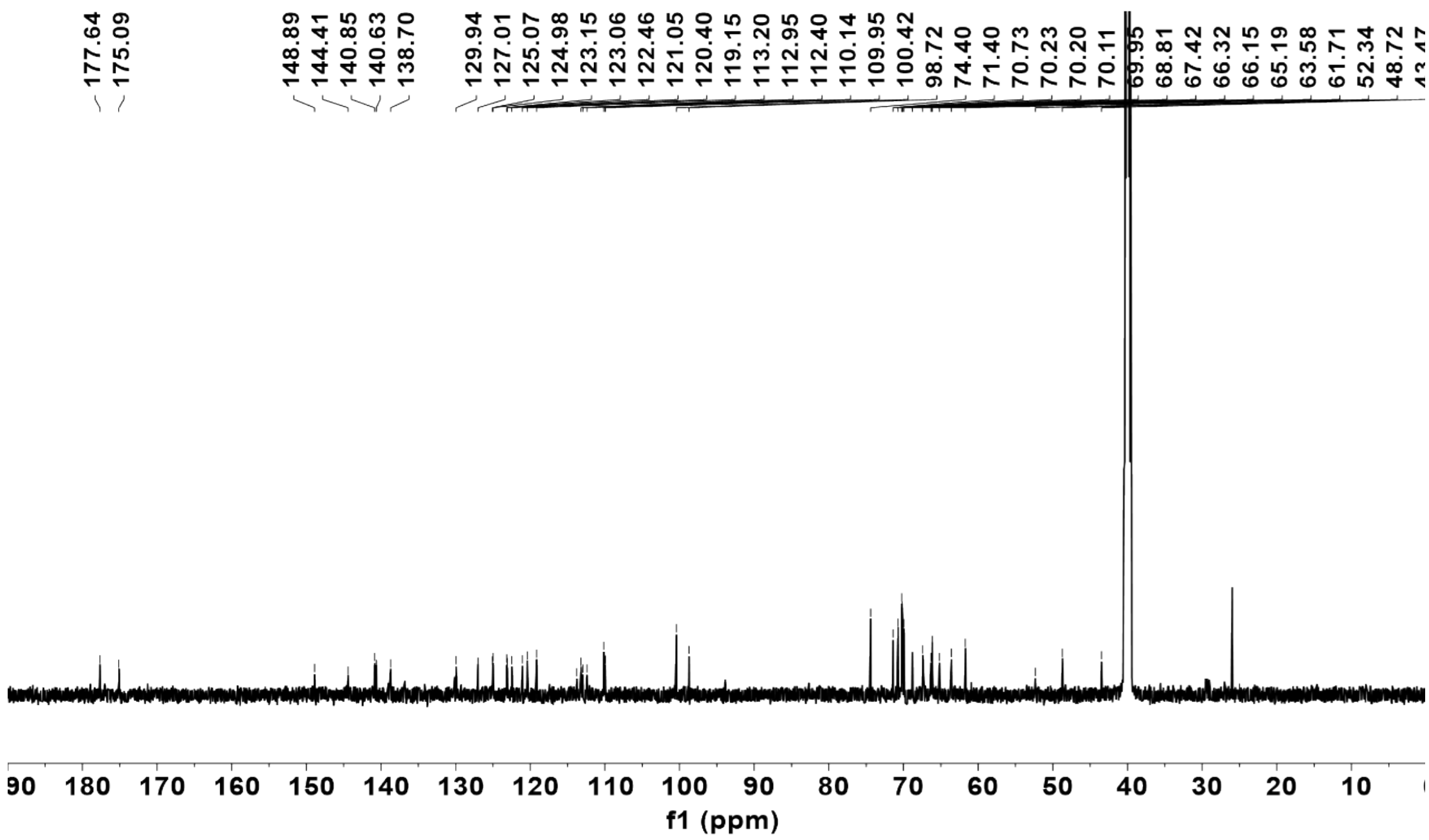

Figure S28. ^{1}H NMR and ^{13}C NMR of compound **TC2M**.

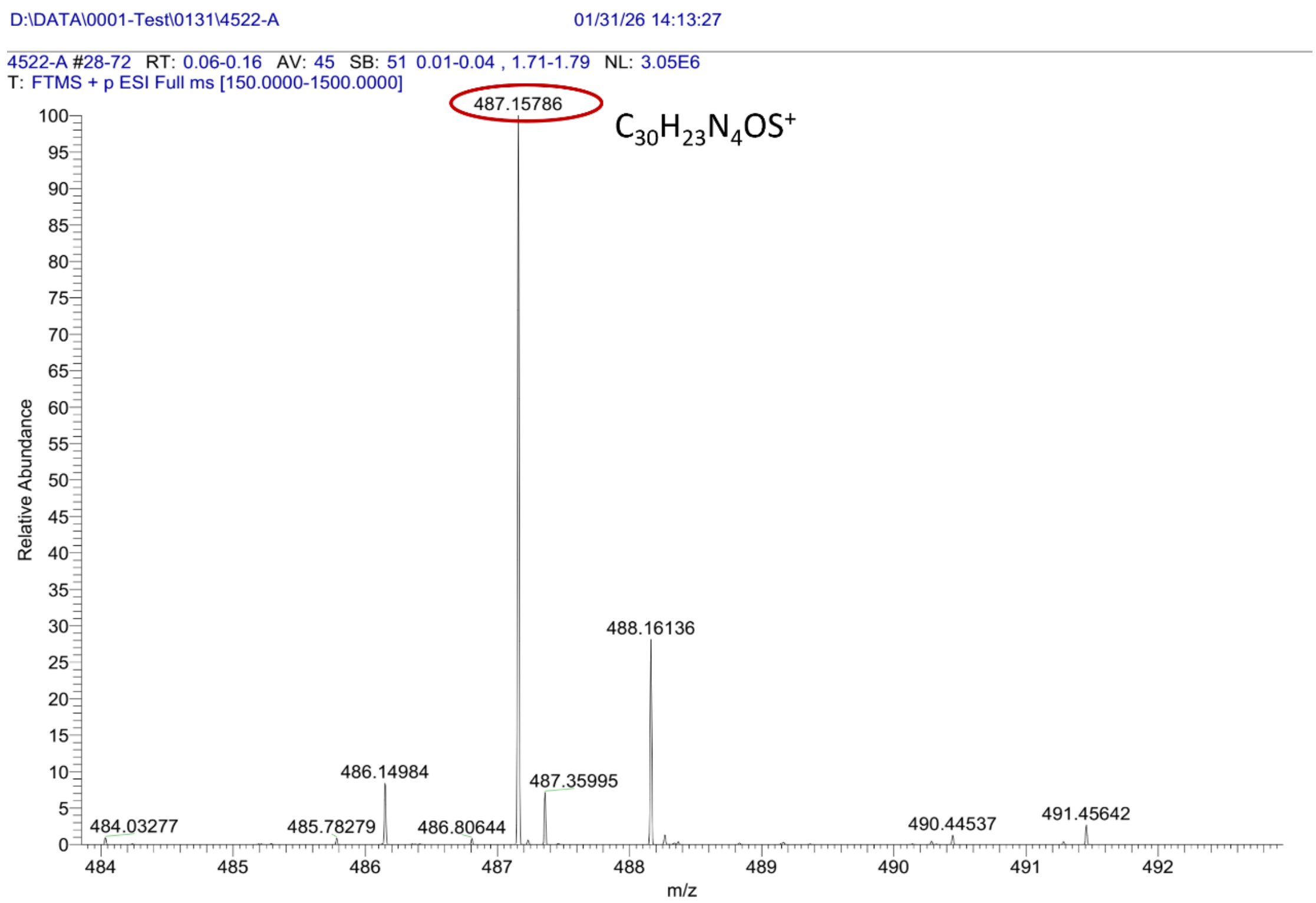

Figure S29. HRMS spectra of compound **TC1**.

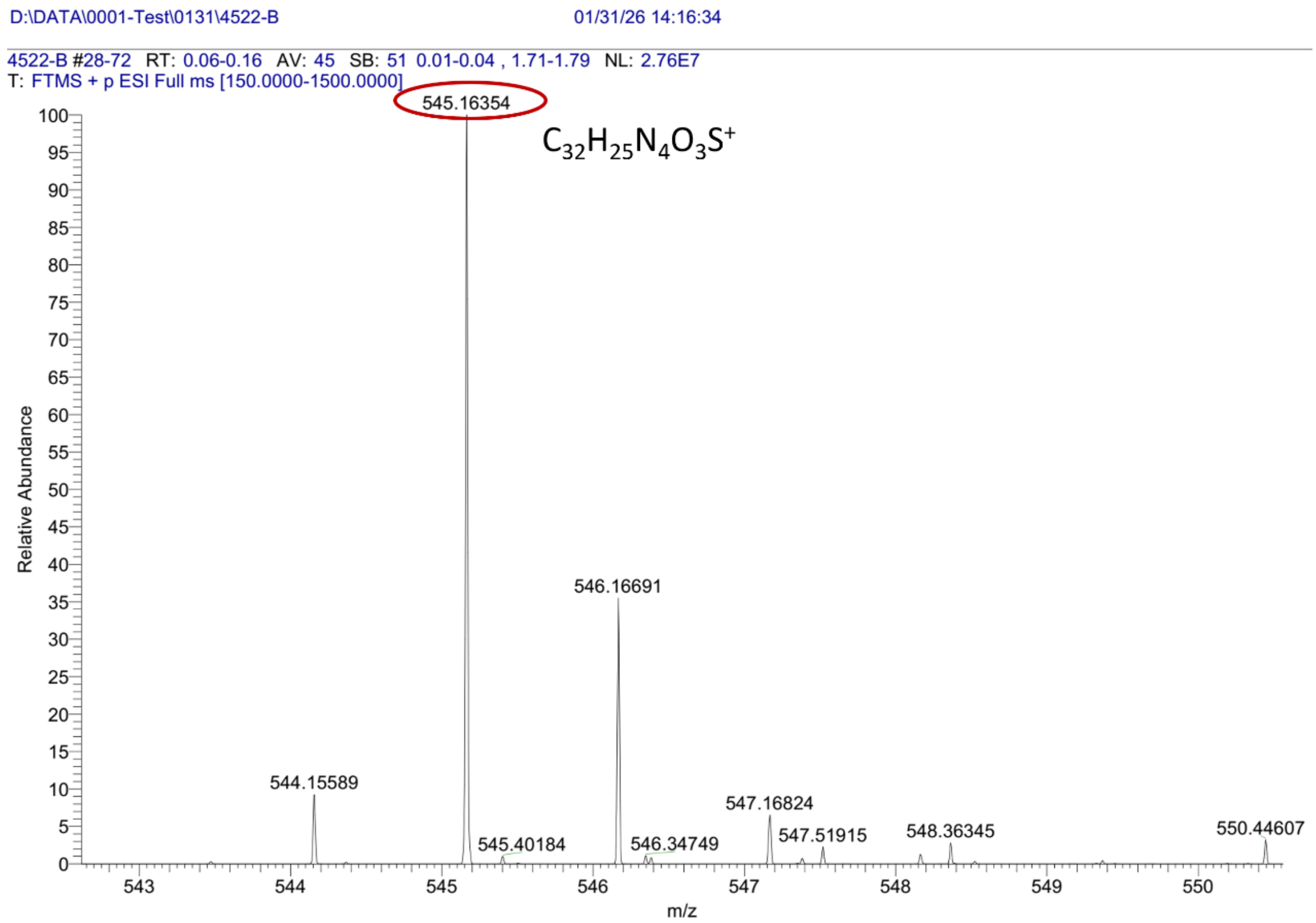

Figure S30. HRMS spectra of compound **TC2**.

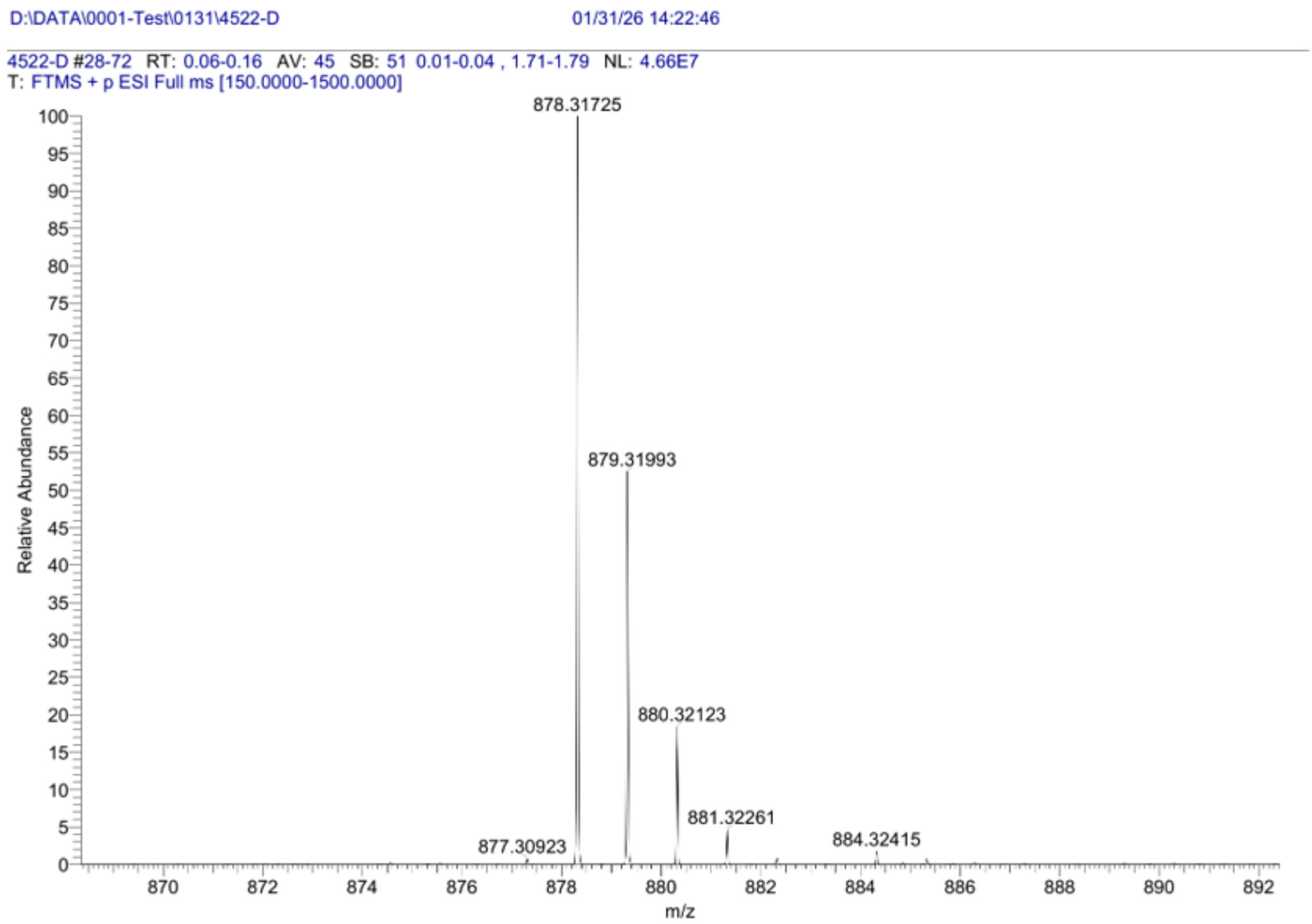

Figure S31. HRMS spectra of compound **TC1M**.

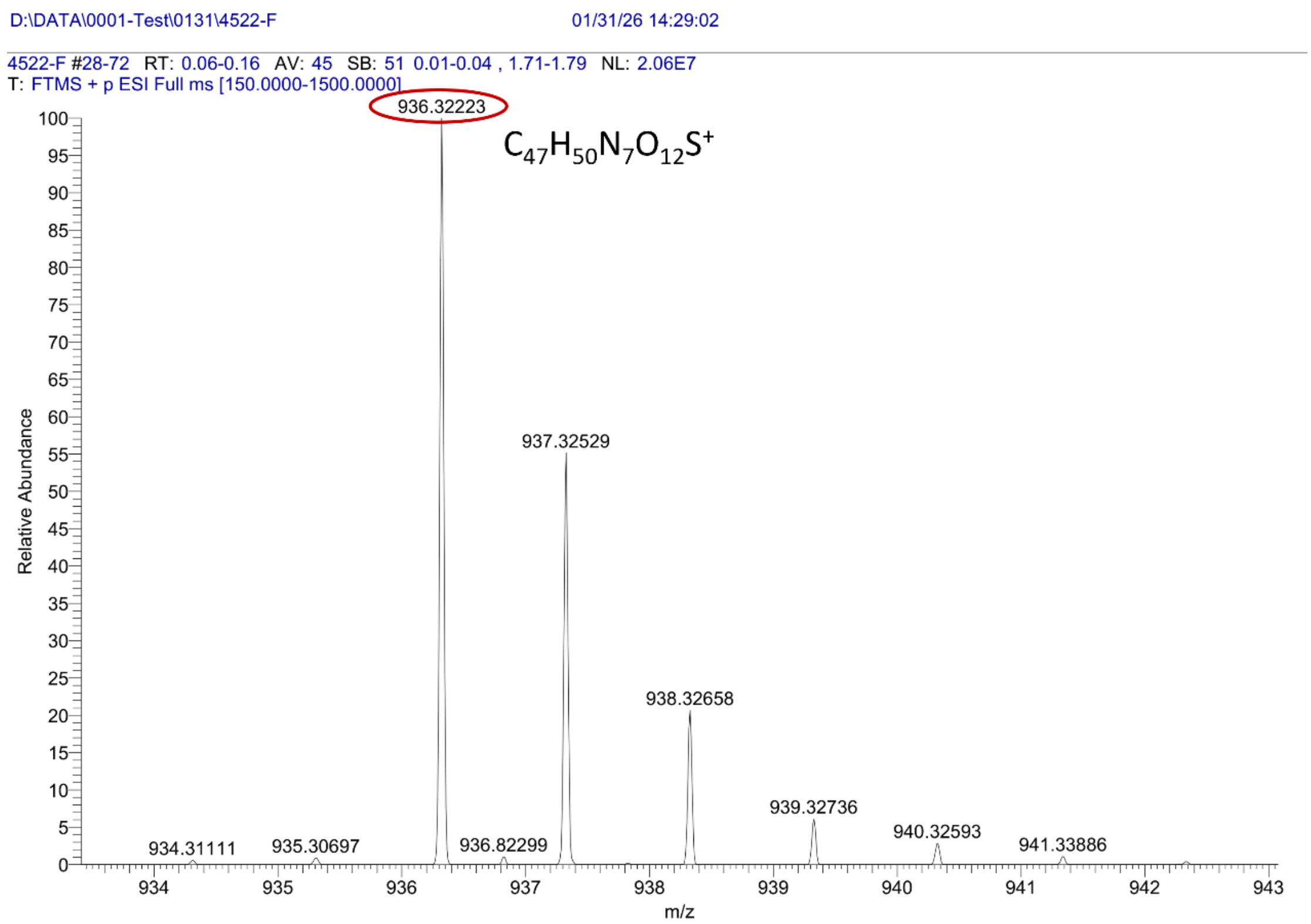

Figure S32. HRMS spectra of compound **TC2M**.

Reference

(1) Gaussian 16, R. B., M. J. Frisch, G. W. Trucks, H. B. Schlegel, G. E. Scuseria, M. A. Robb, J. R. Cheeseman, G. Scalmani, V. Barone, G. A. Petersson, H. Nakatsuji, X. Li, M. Caricato, A. V. Marenich, J. Bloino, B. G. Janesko, R. Gomperts, B. Mennucci, H. P. Hratchian, J. V. Ortiz, A. F. Izmaylov, J. L. Sonnenberg, D. Williams-Young, F. Ding, F. Lipparini, F. Egidi, J. Goings, B. Peng, A. Petrone, T. Henderson, D. Ranasinghe, V. G. Zakrzewski, J. Gao, N. Rega, G. Zheng, W. Liang, M. Hada, M. Ehara, K. Toyota, R. Fukuda, J. Hasegawa, M. Ishida, T. Nakajima, Y. Honda, O. Kitao, H. Nakai, T. Vreven, K. Throssell, J. A. Montgomery, Jr., J. E. Peralta, F. Ogliaro, M. J. Bearpark, J. J. Heyd, E. N. Brothers, K. N. Kudin, V. N. Staroverov, T. A. Keith, R. Kobayashi, J. Normand, K. Raghavachari, A. P. Rendell, J. C. Burant, S. S. Iyengar, J. Tomasi, M. Cossi, J. M. Millam, M. Klene, C. Adamo, R. Cammi, J. W. Ochterski, R. L. Martin, K. Morokuma, O. Farkas, J. B. Foresman, and D. J. Fox, Gaussian, Inc., Wallingford CT, 2016.

(2) Lu, T. A comprehensive electron wavefunction analysis toolbox for chemists, Multiwfn. *J. Chem. Phys.* **2024**, *161*, 802503.

(3) Lu, T.; Chen, F. Multiwfn: A multifunctional wavefunction analyzer. *J. Comput. Chem.* **2012** , *33*, 580-592.

(4) Liu, Z.; Lu, T.; Chen, Q. An sp-hybridized all-carboatomic ring, cyclo[18]carbon: Electronic structure, electronic spectrum, and optical nonlinearity. *Carbon* **2020**, *165*, 461-467.
